# ATM1, an essential conserved transporter in Apicomplexa, bridges mitochondrial and cytosolic [Fe-S] biogenesis

**DOI:** 10.1371/journal.ppat.1012593

**Published:** 2024-09-30

**Authors:** Deepti Shrivastava, Ernest Abboud, Jadhav Prasad Ramchandra, Akanksha Jha, Jean-Baptiste Marq, Animesh Chaurasia, Kalyan Mitra, Mohammad Sadik, Mohammad Imran Siddiqi, Dominique Soldati-Favre, Joachim Kloehn, Saman Habib

**Affiliations:** 1 Division of Biochemistry and Structural Biology, CSIR-Central Drug Research Institute, Lucknow, India; 2 Academy of Scientific and Innovative Research (AcSIR), Ghaziabad, India; 3 Department of Microbiology and Molecular Medicine, CMU, University of Geneva, Geneva, Switzerland; 4 Sophisticated Analytical Instrument Facility and Research Division, CSIR-Central Drug Research Institute, Lucknow, India; Drexel University College of Medicine, UNITED STATES OF AMERICA

## Abstract

The Apicomplexa phylum encompasses numerous obligate intracellular parasites, some associated with severe implications for human health, including *Plasmodium*, *Cryptosporidium*, and *Toxoplasma gondii*. The iron-sulfur cluster [Fe-S] biogenesis ISC pathway, localized within the mitochondrion or mitosome of these parasites, is vital for parasite survival and development. Previous work on *T*. *gondii* and *Plasmodium falciparum* provided insights into the mechanisms of [Fe-S] biogenesis within this phylum, while the transporter linking mitochondria-generated [Fe-S] with the cytosolic [Fe-S] assembly (CIA) pathway remained elusive. This critical step is catalyzed by a well-conserved ABC transporter, termed ATM1 in yeast, ATM3 in plants and ABCB7 in mammals. Here, we identify and characterize this transporter in two clinically relevant Apicomplexa. We demonstrate that depletion of *Tg*ATM1 does not specifically impair mitochondrial metabolism. Instead, proteomic analyses reveal that *Tg*ATM1 expression levels inversely correlate with the abundance of proteins that participate in the transfer of [Fe-S] to cytosolic proteins at the outer mitochondrial membrane. Further insights into the role of *Tg*ATM1 are gained through functional complementation with the well-characterized yeast homolog. Biochemical characterization of *Pf*ATM1 confirms its role as a functional ABC transporter, modulated by oxidized glutathione (GSSG) and [4Fe-4S].

## Author summary

The Apicomplexa phylum includes harmful parasites like *Plasmodium falciparum* and *Toxoplasma gondii*, posing significant threats to human health. Understanding their survival mechanisms is crucial for developing effective treatments. Here, our focus is on iron-sulfur cluster [Fe-S] synthesis pathways found in the mitochondrion and cytosol, essential for the function of many enzymes. Although the transporter responsible for moving sulfur-containing compounds from mitochondria to the cytosol is known in other organisms, it remains unidentified in apicomplexans. Our study identifies *Pf*ATM1 and *Tg*ATM1 as critical transporters in the mitochondrial membrane of these parasites. Downregulation or overexpression of *Tg*ATM1 levels do not hinder mitochondrial functions but affect the level of other proteins, suspected to donate [Fe-S] to cytosolic proteins. We further confirm the transporter’s role by rescuing *Tg*ATM1 depleted parasites with the yeast homolog *Sc*ATM1. Additionally, we identify glutathione-coordinated [4Fe-4S] as a probable substrate for *Pf*ATM1. These findings provide valuable insights into how parasites manage essential resources, potentially opening new avenues for targeted drug development.

## Introduction

[Fe-S] are believed to be the oldest post-translational protein modification known in nature. They mediate electron transport reactions and catalysis in many critical processes such as DNA repair, protein translation, tricarboxylic acid (TCA) cycle, electron transport chain (ETC) and iron homeostasis [1–3]. [Fe-S] biogenesis is mediated by the ISC (iron-sulfur cluster), NIF (nitrogen fixation) and SUF (sulfur mobilization) pathways; ISC is found in bacteria and most Eukarya, NIF is found in bacteria, and SUF is the backup pathway in most bacteria and is also present in archaea, protozoa and plants [4, 5]. [Fe-S], most commonly [2Fe-2S] and [4Fe-4S], on cytosolic and nuclear proteins in Eukarya are delivered via the cytosolic iron-sulfur cluster assembly (CIA) pathway [6, 7]. The CIA pathway, which lacks cysteine desulfurase for sulfur mobilization, comprises a set of five to six cytosolic proteins with its NBP35-CFD1 scaffold complex serving as the primary recipient of [Fe-S] or their intermediates from mitochondrial ISC [6, 8, 9]. An exporter of [Fe-S]/intermediates is thus required at the mitochondrial membrane. This transporter has been first identified and characterized in yeast, named ATP binding cassette (ABC) transporter mitochondria type-1 (*Sc*ATM1), and localized to the inner membrane of the mitochondrion [7, 10, 11]. Subsequently, homologs have been identified in plants and mammals and named ATM3 and ABCB7, respectively [12, 13].

The supply of [Fe-S] from mitochondrial ISC to cytosolic CIA by the yeast mitochondrial transporter *Sc*ATM1 and its homologs has been proposed to be via transport of glutathione-coordinated [2Fe-2S], termed [2Fe-2S]GS_4_. An alternative cargo comprising a polysulfide adduct of oxidized glutathione with a possible role in cytosolic tRNA thiolation has been suggested from experiments conducted with *Sc*ATM1 and *Arabidopsis thaliana* ATM3 [7, 12]. Hence, the precise substrate(s) of ATM1 and its homologs remains elusive. ATMs belong to superfamily B of ABC transporters and are called ‘half-transporters’ because they form homodimers to facilitate active transport [14]. Each ATM comprises an N-terminal transmembrane domain and a C-terminal nucleotide-binding domain (NBD) [15]. Cargo is transported through a classical switch mechanism where movements of the NBD and transmembrane regions are coupled to catalyze transfer of cargo across the inner mitochondrial membrane [13, 16, 17]. Members of the ABC superfamily B include the biologically important multidrug resistance (MDR) proteins, iron/[Fe-S] transporters, heavy metal resistance proteins and the transporter associated with antigen processing (TAP) complex [18, 19]. Mutations in the ATM1 homolog in humans, ABCB7, results in severe disease X-linked sideroblastic anemia with ataxia which is characterized by the loss of cytosolic [Fe-S] proteins due to a CIA defect, an accumulation of iron in the mitochondria, and a defect of heme biosynthesis [20–22]. *Saccharomyces cerevisiae* lacking *Sc*ATM1 (Δ*Sc*ATM1) show sensitivity to oxidative stress and exhibit a 30-fold increase in mitochondrial iron accumulation [23]. Comparative analyses of the transcriptional responses in yeast lacking *Sc*ATM1 has revealed disruptions in several pathways, leading to the upregulation of genes involved in cytosolic iron uptake (Aft1/2), decreased heme levels, and reduced activity of the heme-containing complex IV of the electron transport chain (ETC) [24].

The phylum Apicomplexa groups thousands of obligate intracellular parasites infecting a broad range of hosts; *Plasmodium* and *Cryptosporidium* species as well as *Toxoplasma gondii* represent severe threats for human health. In most apicomplexan parasites, [Fe-S] biogenesis occurs in three subcellular compartments—the cytosol (CIA), the mitochondrion or mitosome in case of *Cryptosporidum* (ISC), and the apicoplast (SUF), a relict plastid organelle derived from secondary endosymbiosis [25–32]. [Fe-S] biogenesis via the *Plasmodium* apicoplast SUF pathway is crucial for parasite survival in asexual blood stages [27] and development of sporozoites in mosquitoes [29]. In *T*. *gondii*, downregulation of the scaffold protein *Tg*ISU1 disrupts mitochondrial ISC, which is essential for maintaining cellular respiration. This disruption triggers the differentiation of the parasite into bradyzoites, the slow-growing encysted stage responsible for chronic infection [33]. Depletion of the apicoplast desulfurase *Tg*NFS2 or a component of the scaffold complex (*Tg*SUFC) irreversibly impacts parasite fitness and disturbs critical apicoplast pathways relying on [Fe-S] proteins [33, 34]. A distinctive feature of *Plasmodium falciparum* ISC is that its scaffold protein *Pf*IscU directly assembles [4Fe-4S] clusters without [2Fe-2S] intermediates [31]. [4Fe-4S] are transferred to recipient apo-proteins directly by *Pf*IscU or via cluster carrier proteins such as *Pf*IscA2 [31]. In *T*. *gondii*, the first protein of the CIA pathway to be functionally characterized is the scaffold protein Nucleotide Binding Protein 35 (*Tg*NBP35) [35]. Unexpectedly, in contrast to the cytosolic NBP35 of human, yeast, and plants, *Tg*NBP35 is anchored to the outer membrane of the mitochondrion, where it is critical for the initiation of the CIA and important for parasite fitness [35]. Although many components of the [Fe-S] synthesis pathways are known in Apicomplexa, the transporter homolog of ATM1, which connects mitochondria-generated [Fe-S] with the CIA, remains uncharacterized in the phylum.

In *T*. *gondii*, *Tg*ATM1 (TGGT1_269000), termed ABCB7-like (ABCB7L) in a recent study [36], was previously proposed as the putative *Sc*ATM1 homolog [33, 37]. Consistent with this function, it was localized to the mitochondrial membranes in a spatial proteomics study [38] and assigned a very low fitness score in a whole-genome CRISPR/Cas9 screen, suggesting a fitness-conferring role [39]. However, *Tg*ATM1 also has homology to human ABCB6, a porphyrin transporter located at the outer mitochondrial membrane, implicated in heme synthesis [40]. Consequently, *Tg*ATM1 was also speculated to hold this function in the parasite [41, 42].

In *P*. *falciparum*, out of a total of seven ABCB superfamily members (ABCB1-ABCB7) [43, 44], ABCB6 (*Pf*MDR6/ *Pf*ATM1, PF3D7_1352100) was identified as the plausible mitochondrial *Sc*ATM1 homolog. An essential role of *Plasmodium* ATM1 during blood stage development has been indicated as *Pf*ATM1 knock-out parasites could not be isolated in *P*. *berghei* or *P*. *falciparum* [45, 46]. However, this was contradicted by a report where *Pb*ABCB6 (*Pb*ATM1) could be deleted in blood stage parasites [47]. Poly(Asn) repeats in *Pf*ATM1 have also been associated with differential drug susceptibility in field isolates from Africa and South Asia [48–50].

In this study, we identified *Tg*ATM1 and *Pf*ATM1 as the transporters likely facilitating movement of [Fe-S] complexes and sulfur-containing intermediates across the mitochondrial membrane in parasites. Downregulation of either transporter significantly affected parasite physiology. We successfully complemented the *Tg*ATM1 mutant with *Sc*ATM1 and confirmed a similar role for *Pf*ATM1. While *Tg*ATM1 downregulation or overexpression did not specifically impact mitochondrial metabolism, its levels inversely correlated with the expression of a few [Fe-S] proteins. In mammalian cells, these affected proteins have been proposed to localize to the outer mitochondrial membrane, transferring mitochondrially generated [Fe-S] complexes to cytosolic proteins. Based on experiments with heterologously expressed proteins, *Pf*ATM1 was shown to receive [4Fe-4S] from mitochondrial ISC scaffold proteins and interact with cytosolic CIA protein *Pf*NBP35. Biochemical assays confirmed *Pf*ATM1 as a functional ABC transporter capable of ATP hydrolysis, stimulated by oxidized glutathione (GSSG) and [4Fe-4S], and transporting GSSG cargo. These findings highlight the importance of this conserved mitochondrial transporter in apicomplexan parasites.

## Results

### A mitochondrial ATM1 homolog is conserved across Apicomplexa

To confirm the identity of ATM1 homologs in *P*. *falciparum* and *T*. *gondii*, we conducted homology searches using sequences from *Sc*ATM1, human ABCB7, and *At*ATM3 as queries [51] in PlasmoDB and ToxoDB databases. TGGT1_269000 was identified as the putative *T*. *gondii* ATM1; two homologs, *Pf*ABCB2 (*Pf*MDR2, PF3D7_1447900) and *Pf*ABCB6 (*Pf*MDR6, PF3D7_1352100) were initially considered in *P*. *falciparum*. However, a mitochondrial targeting sequence (MTS) was not identifiable in *Pf*ABCB2 whereas *Pf*ABCB6 had a predicted MTS (Mitoprot II prediction scores of 0.0218 and 0.9434 for *Pf*ABCB2 and *Pf*ABCB6, respectively). Phylogenetic analysis with ATM1 and HMT (heavy metal tolerance) family proteins from *Drosophila melanogaster*, yeast, human and *Arabidopsis thaliana* also suggested closer proximity of *Pf*ABCB2 to HMTs compared to *Pf*ABCB6 (**S1A Fig**). *Pf*ABCB2 formed a separate clade with eukaryotic HMTs, sharing a more recent common ancestor with them. *Pf*ABCB2 has been previously reported to localize to the parasite plasma membrane/food vacuole [52, 53]. It has also been demonstrated to play a role in heavy metal resistance in the parasite by mediating active extrusion of Cd^2+^ out of the cytosol [54], and likely belongs to the closely related HMT family. Thus, *Pf*ABCB6/*Pf*MDR6 was identified as the putative *Pf*ATM1.

Subcellular localization data obtained from isotope tagging (LOPIT) indicated mitochondrial localization for *Tg*ATM1 [38]. Additionally, whole-genome mutant screens in both parasites revealed a very low fitness score for these proteins, suggesting an important role in parasite survival [39, 55]. Homologs of these proteins were identified in other apicomplexan parasites through BLASTp searches, including the ABC transporter of *Theileria orientalis*, ABCB25 of *Babesia bovis*, and ATM1-like protein of *Cryptosporidium parvum* (**S1 Table**). Overall, this transporter is highly conserved within the phylum, even in species harboring a remnant mitochondrion (mitosome) that lacks a functional heme synthesis pathway.

**Fig 1:**
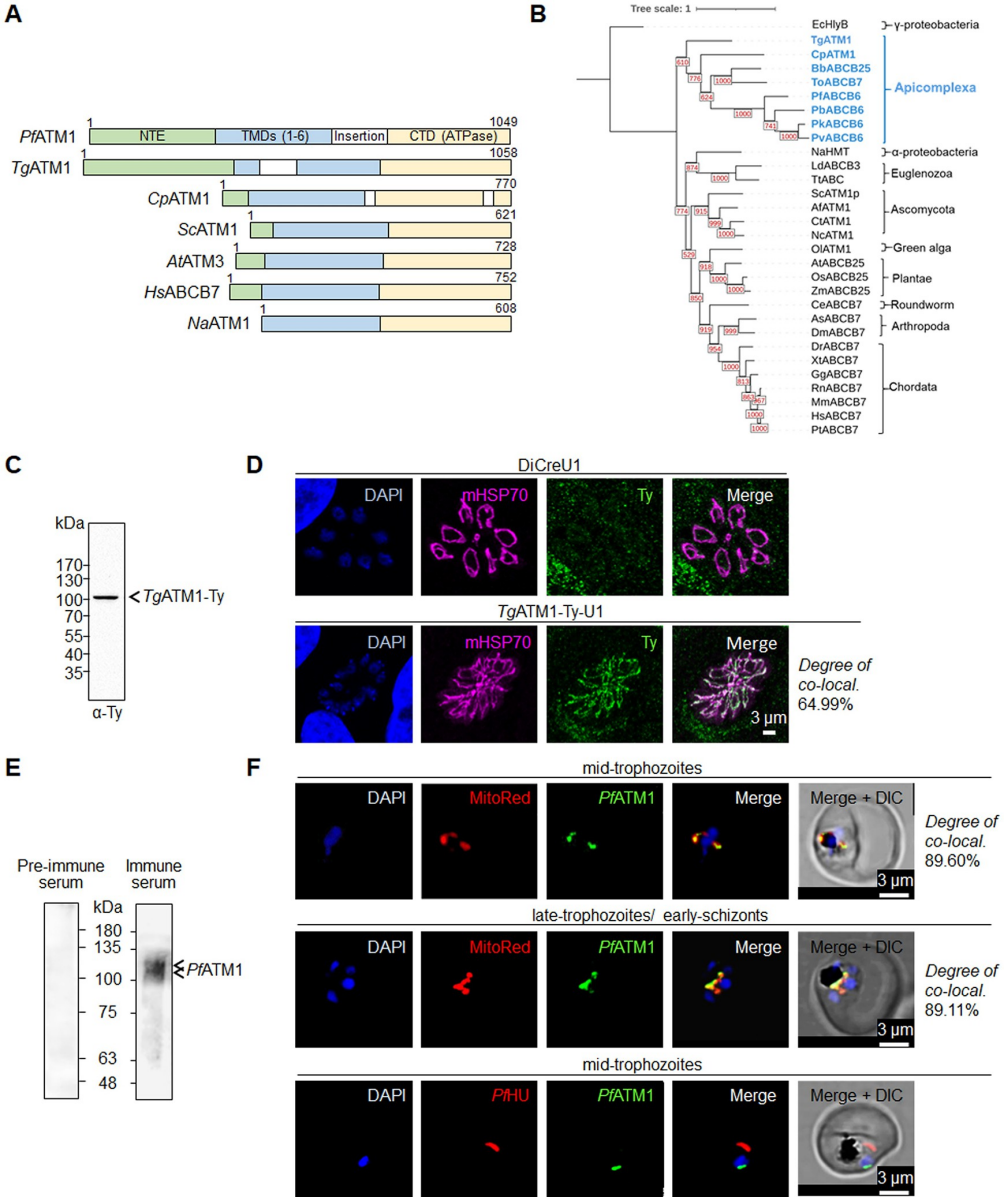
Phylogeny and localization of *Pf*ATM1 and *Tg*ATM1. (**A**) Domain organization and N-terminal extensions (NTE) in ATM1 homologs from apicomplexan parasites (*P*. *falciparum*, *T*. *gondii*, *C*. *parvum*), *Saccharomyces cerevisiae*, *A*. *thaliana*, *Homo sapiens* and the bacterium *N*. *aromaticivorans*. (**B**) Phylogeny of ATM1 homologs generated by PhyML 3.0. The tree was rooted at the *Ec*HlyB branch. Bootstrap values of 1000 replicates are indicated at each branch. (**C**) Western blot of a *Tg*ATM1-Ty-U1 parasite lysate probed with anti-Ty antibody. (**D**) Immunofluorescence assay of parental (DiCreU1) and *Tg*ATM1-Ty-U1 parasites stained with anti-Ty antibody and a mitochondrial marker (mitochondrial heat shock protein, mHSP70). The degree of colocalization is given. Images shown in C and D are representative of three independent biological replicates. (**E**) Western blot of *P*. *falciparum* lysate probed with anti-*Pf*ATM1 serum recognizes a specific band at the expected size of unprocessed *Pf*ATM1 (123 kDa) and a closely migrating lower band likely representing processed protein. **(F**) Immunofluorescence assay for subcellular localization of *Pf*ATM1 at different intra-erythrocytic stages. Scale (0–3 μm) is shown in the merged image. DAPI is the nuclear stain, Mitotracker Red is the mitochondrial marker dye, anti-*Pf*ATM1 antibodies and anti-*Pf*HU serum detect *Pf*ATM1 and the apicoplast marker *Pf*HU, respectively. Colocalization rate is given for overlay of *Pf*ATM1 and Mitotracker Red signals.

In contrast to the well-characterized α-proteobacterium *Novosphingobium aromaticivorans* ATM1, *Pf*ATM1 and *Tg*ATM1 exhibit a substantial unconserved N-terminal extension (NTE) preceding the N-terminal domain (NTD) and transmembrane region (**Figs 1A and S1B)**. Alignment with homologs from yeast, human, and plants revealed the preservation of crucial structural components of ATMs (**S1B Fig)**. Conserved glutathione binding sites and residues demonstrated to be complexed with [2Fe-2S] in *Na*ATM1 and other homologs were identified between TM4 and TM5 and downstream of TM5 [16, 56] **(S1B Fig**). The C-terminal ATPase domain (CTD) contains conserved Walker A, Walker B and ABC signature motifs. *Pf*ATM1 and *Tg*ATM1 carry diverged insertions between the transmembrane region and CTD in the former, and between TM1 and TM2 in the latter (**Figs 1A and S1B)**. The evolutionary proximity of apicomplexan ATM1 homologs was inferred by constructing a maximum likelihood phylogenetic tree (**Fig 1B**). The apicomplexan sequences formed a separate clade branching at the root of the tree and are sister to the basal clade of all other ATM1 homologs.

To assess the localization and significance of *Tg*ATM1, we inserted 3-Ty tags into the native locus for localization studies, alongside loxP sites and a U1 recognition site for conditional downregulation upon rapamycin (Rapa) induction [57] (**S2A Fig**). The modification was performed using CRISPR/Cas9 editing [58] in a line stably expressing the dimerizable Cre recombinase (DiCreU1) and the resulting strain was labelled as *Tg*ATM1-Ty-U1 [57]. Transfected parasites were selected and cloned through limited dilution. Correct integration in the 3′ untranslated region (UTR) of *Tg*ATM1 was validated in a clonal population by genomic PCR (**S2B Fig, S2 Table**). *Tg*ATM1-Ty was detected by western blot in *Tg*ATM1-Ty-U1 parasites, running slightly smaller than its anticipated size of 126 kDa, a common occurrence for transmembrane proteins (**Fig 1C**). Immunofluorescence assays (IFAs) coupled with confocal microscopy revealed that *Tg*ATM1-Ty exhibited co-localization with the mitochondrial marker mitochondrial heat shock protein 70 (mHsp70) [59] (**Fig 1D**), confirming its mitochondrial localization consistent with the previously determined localization by isotope tagging (LOPIT) [38].

To ascertain the subcellular localization of *Pf*ATM1, we expressed its unconserved NTE domain (*Pf*ATM1-NTE, 33 kDa) as a 6X-His tagged protein in *E*. *coli* (**S3A Fig**) and utilized the purified protein to produce antibodies in rabbits. The antiserum detected a distinct band around ~123 kDa, aligning with the anticipated size of unprocessed *Pf*ATM1 (**Fig 1E)**. Additionally, a closely migrating lower band was observed, possibly indicating a processed or degraded product. IFAs using purified *Pf*ATM1 Abs showed clear partitioning of *Pf*ATM1 signal to the parasite mitochondrion, merging with Mitotracker Red across various asexual stages (**Fig 1F**). No overlap was seen with the apicoplast marker *Pf*HU [60] (**Fig 1F**), confirming that *Pf*ATM1, like *Tg*ATM1, localizes to the mitochondrion.

### *Tg*ATM1 and *Pf*ATM1 are required for optimal parasite growth

**Fig 2:**
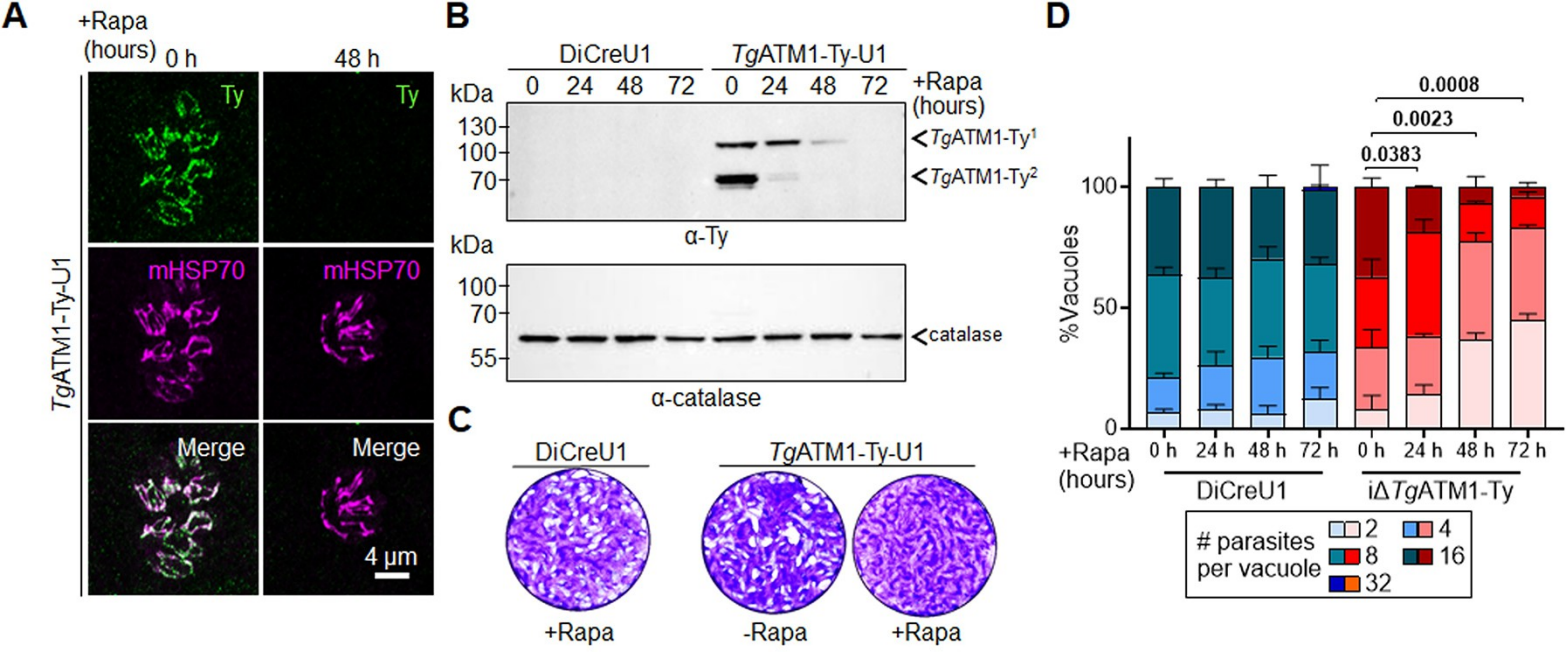
*Tg*ATM1 and *Pf*ATM1 are essential for parasite growth. (**A**) Immunofluorescence assays of *Tg*ATM1-Ty-U1 parasites stained with anti-Ty antibody and a mitochondrial marker (mitochondrial heat shock protein, mHSP70) following 0 or 48 hours of rapamycin (Rapa) treatment. (**B**) Western blot of parental (DiCreU1) and *Tg*ATM1-Ty-U1 parasite lysates following varying durations of Rapa treatment. The membrane was probed with anti-Ty antibody and anti-catalase antibody as loading control. Two major bands were observed for *Tg*ATM1-Ty, labelled *Tg*ATM1-Ty^1^ and *Tg*ATM1-Ty^2^. (**C**) Stained host cell monolayers revealing plaques formed by parental (DiCreU1) or *Tg*ATM1-Ty-U1 parasites over seven days in presence or absence of Rapa. A-C: Representative images are shown from one of 3 independent biological replicates. (**D**) Intracellular growth assay displaying the proportion of vacuoles containing 2, 4, 8, 16 and 32 parasites after 24 hours of intracellular growth and varying durations of Rapa treatment for parental (DiCreU1) and *Tg*ATM1-Ty-U1 parasites. The means and standard deviations from three independent biological replicates are shown. A two-sided Student’s t-test was used to compare the average number of parasites between the indicated conditions. Significant differences (p <0.05) are highlighted in bold.

Next, we employed conditional downregulation to probe the importance and function(s) of *Tg*ATM1. Effective depletion of *Tg*ATM1-Ty upon Rapa induction in *Tg*ATM1-Ty-U1 parasites was validated via IFA (**Fig 2A**), with no detectable signal observed after 48 hours of Rapa treatment. Efficient downregulation of *Tg*ATM1-Ty was further confirmed by western blot, revealing a gradual depletion and undetectable levels after 72 hours of Rapa (**Fig 2B**). Intriguingly, two bands were observed for *Tg*ATM1 in some but not all western blots, with the lower band at 70 kDa likely reflecting a degradation product or a processed form. Based on the kinetics of *Tg*ATM1 downregulation, most of the subsequent assays were performed after 72 h. To assess the significance of *Tg*ATM1 in the lytic cycle of *T*. *gondii*, we conducted plaque assays using human foreskin fibroblasts (HFFs) infected with the parental strain (DiCreU1) and *Tg*ATM1-Ty-U1 parasites with or without Rapa (+Rapa and −Rapa), respectively. Monolayers were fixed and stained with crystal violet 7 days post infection. Depletion of *Tg*ATM1 severely affected the formation of plaques confirming its crucial role in one or more steps of the parasite’s lytic cycle (**Fig 2C).** To assess if the hindered lytic cycle results from a growth deficiency, we quantified the parasite number per vacuole after 24 hours of intracellular growth and varying durations of Rapa treatment (**Fig 2D**). A subtle but significant growth defect was evident after just 24 hours of *Tg*ATM1 downregulation. The average number of parasites per vacuole decreased from >9 in the controls (DiCreU1 +Rapa and *Tg*ATM-Ty-U1 −Rapa) to 7.7, 4.7 and 4.1 after 24, 48 hours and 72 hours of Rapa treatment, respectively (**Fig 2D**). These findings validate the significance of *Tg*ATM1 in the parasite’s lytic cycle and replication.

The essentiality of *Pf*ATM1 in parasite physiology is suggested from results of random piggyBac transposon mutagenesis screen in *P*. *falciparum* [55]. There are, however, contradictory reports concerning the *P*. *berghei* ortholog (*Pb*ABCB6, PBANKA_1364800); it has been deemed essential through direct genetic knockout (PlasmoGEM, [46]), but another study described that its deletion does not affect viability of blood stage parasites [47]. We thus attempted downregulation of *Pf*ATM1 using the Vivo-morpholino approach, a method previously reported for targeted translation inhibition in *P*. *falciparum* [61]. A specific Vivo-morpholino (*Pf*ATM1-V_m_) was designed (by GeneTools, USA) to inhibit the translation of *Pf*ATM1 **(S3B Fig**). Treatment of *P*. *falciparum* 3D7-infected cells with *Pf*ATM1-V_m_ resulted in lower levels of the target at 72 hours and 96 hours post treatment (**S3C and S3D Fig**). Quantitative analysis of the western blots indicated significant reduction (~57%) in actin-normalized *Pf*ATM1 levels in *Pf*ATM1-V_m_ treated cells at the 96 hours’ time point when compared with untreated cells and control V_m_ (**S3D Fig**). The control V_m_ is a random sequence with no significant identity to the *P*. *falciparum* genome. Comparison of erythrocytic stages at different time points post-treatment revealed a growth delay in treated cells, particularly noticeable at 96 and 108 hours (**S3E and S3F Fig).** At 96 h, untreated and control V_m_-treated cells progressed to the early/mid-trophozoite stage, while *Pf*ATM1-V_m_-treated cells remained predominantly at the ring stage. By 108 hours, control cells were mainly at the mid- to late-trophozoite stage, whereas *Pf*ATM1-V_m_-treated cells were still in the early trophozoite stage (**S3E and S3F Fig**). Neither a significant reduction in parasitemia (**S3F Fig**), nor apparent alterations in mitochondrial morphology were observed in *Pf*ATM1-V_m_ treated cells at these time points (**S3G Fig**). At the later time point of 144 h, control and V_m_-treated cells showed similar stages suggesting that *Pf*ATM1-V_m_ had lost its effect after the third cycle (**S3E and S3F Fig**). This was corroborated by western blot analysis, which showed that the levels of *Pf*ATM1 in *Pf*ATM1-V_m_-treated cells returned to normal by 144 hours (**S3C and S3D Fig**). Reducing *Pf*ATM1 levels results in a slowdown of asexual parasite growth in blood stages. Direct genetic knockdown of *Pf*ATM1 (*e*.*g*., using the aptamer/TetR-DOZI system) would be required for conclusive evidence of its essentiality in the parasite.

Taken together, these findings reveal the essential role of *Tg*ATM1; the slow growth observed in *Pf*ATM1 knockdown and the reported essentiality of *P*. *berghei* ABCB6/MDR6 [46] supports their role as mitochondrial transporters required for optimal development of both parasites.

### *Tg*ATM1 and *Pf*ATM1 participate in [Fe-S] proteins homeostasis but not in mitochondrial function

**Fig 3:**
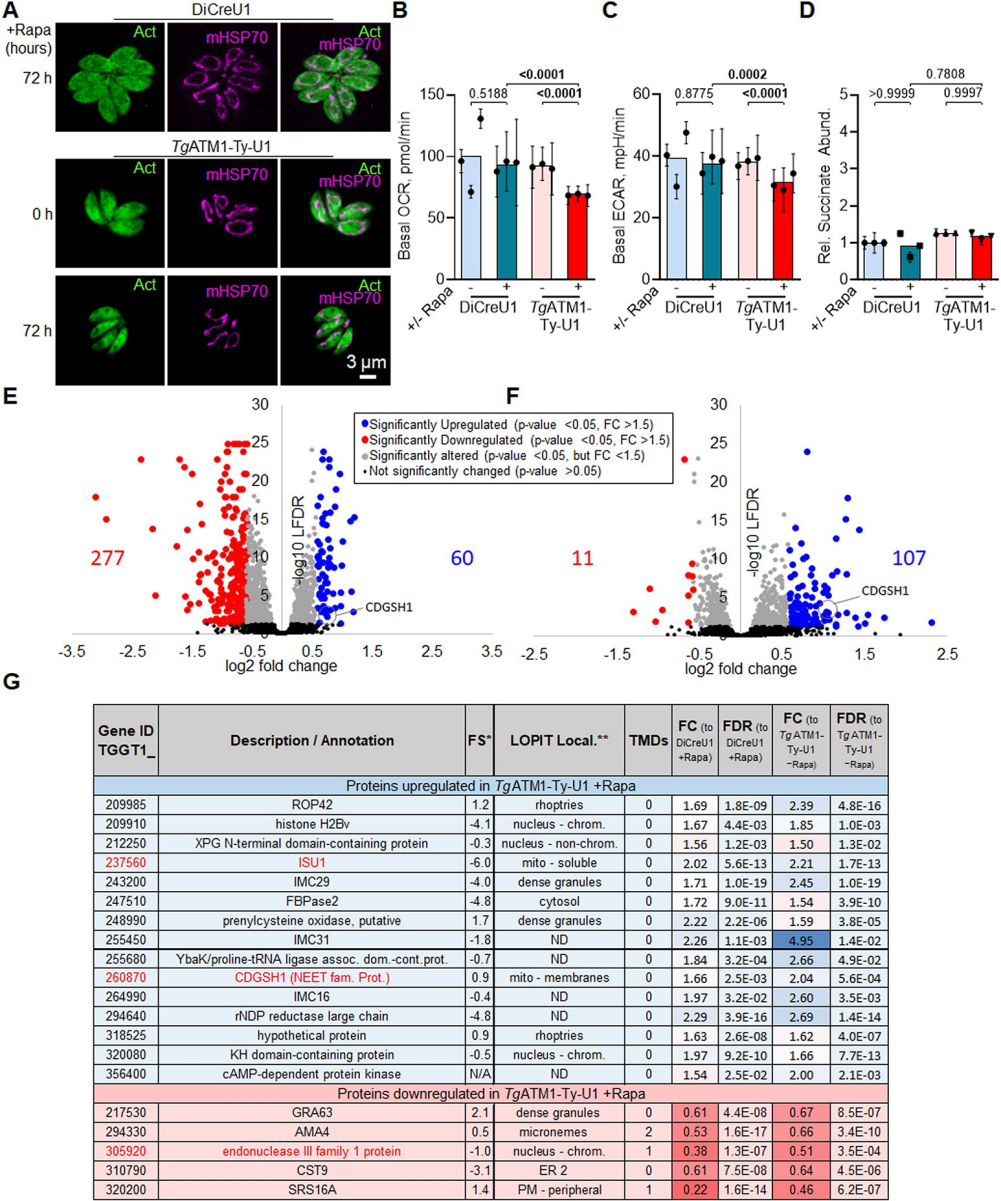
Downregulation of ATM1 affects iron-sulfur cluster proteins in *Toxoplasma* and iron levels in *Plasmodium*. (**A**) Immunofluorescence assays of DiCreU1 (parental control) and *Tg*ATM1-Ty-U1 parasites following 0 and 72 hours of rapamycin (Rapa) treatment. Parasites were visualized using anti-actin (Act) antibodies, while mitochondria were stained using antibodies against the mitochondrial heat shock protein (mHSP70). (**B**) and (**C**) Basal oxygen consumption rate (OCR) (B) and extracellular acidification rate (ECAR) (C) following 0 or 72 hours of Rapa treatment in parental (DiCreU1) and *Tg*ATM1-Ty-U1 parasites, as determined using an extracellular flux analyser. (**D**) Relative abundance of the tricarboxylic acid (TCA) cycle intermediate succinate as determined by gas chromatography-mass spectrometry (GC-MS) of parental (DiCreU1) and *Tg*ATM1-Ty-U1 parasite extracts following 0 or 72 hours of Rapa treatment. B-D) The means and standard deviation of three independent biological experiments are shown. Bars represent the means of the three experiments. Conditions were compared using a One-way ANOVA followed by Tukey multiple pair wise comparisons. Significant differences (p <0.05) are highlighted in bold. (**E**)-(**F**) Volcano plot highlighting changes in protein levels of *Tg*ATM1-Ty-U1 parasites after 72 hours of rapamycin treatment compared to parental parasites (DiCreU1) treated equally with Rapa (E) or *Tg*ATM1-Ty-U1 parasites not treated with Rapa (F). Unchanged proteins are displayed in black, while significantly altered proteins are displayed in grey (< 1.5-fold change (FC), local false discovery rate, LFDR < 0.05) or in blue (>1.5-fold increased; LFDR < 0.05) or red (> 1.5-fold decreased; LFDR < 0.05). The number of significantly increased and decreased proteins is given and the NEET protein CDGSH1 is indicated. (**G**) Table listing proteins that are significantly up- or downregulated in both comparisons shown in E and F. [Fe-S] proteins are highlighted in red. Gene accession numbers (identifiers, IDs), the fitness score from a genome-wide CRISPR screen, the putative localization from a spatial proteomics study (localization of organelle proteins by isotope tagging, LOPIT), the number of transmembrane domains (TMDs), as well as the LFDR and FC for both comparisons shown in E and F are given.

Given the mitochondrial localization of *Tg*ATM1, we assessed if its downregulation affects the organelle’s morphology. Previous studies have reported obvious morphological defects in mitochondria, including ‘ball-shaped’ and ‘broken’ mitochondria, upon downregulation of transmembrane proteins in the inner and outer mitochondrial membrane [62, 63]. However, downregulation of *Tg*ATM1 for 72 hours was not associated with any apparent morphological abnormalities, with parasites exhibiting the typical ‘lasso-shaped’ organelle, as observed in the controls (**Fig 3A**). We further scrutinized if the downregulation of *Tg*ATM1 impairs mitochondrial functions. To this end, we assessed the oxygen consumption rate (OCR) as an indicator of mitochondrial respiration using an extracellular flux analyser (Seahorse, Agilent) in both the parental DiCreU1 and *Tg*ATM1-Ty-U1 parasites under conditions of no treatment or after 72 hours of Rapa treatment (**Fig 3B, S3 Table**). The OCR decreased to about 70–80% upon *Tg*ATM1 downregulation compared to its controls (**Fig 3B**). The extracellular acidification rate (ECAR), which was simultaneously quantified, as a measure for glycolysis (**Fig 3C, S3 Table**), similarly decreased to about 70–80% upon downregulation of *Tg*ATM1, compared to its controls.

To further probe mitochondrial function in parasites depleted in *Tg*ATM1, we measured the relative abundance of metabolites related to mitochondrial metabolism, including tricarboxylic acid (TCA) cycle intermediates, via gas chromatography-mass spectrometry (GC-MS). The abundance of succinate (**Fig 3D**), citrate, malate, γ-aminobutyric acid (GABA), aspartate, glutamate and 5-aminolevulinic acid (5-ALA)) did not change significantly (p <0.05, fold change >1.5) upon downregulation of *Tg*ATM1, when compared to both controls (DiCreU1 +Rapa and *Tg*ATM1-Ty-U1 −Rapa) (**S4 Table**). Taken together, these results speak against a specific mitochondrial defect in parasites depleted in *Tg*ATM1. Instead, the decrease in OCR alongside a similar decrease in ECAR indicates a general fitness impairment following *Tg*ATM1 depletion, which is consistent with the growth defect observed at this time point (**Fig 2D**). In contrast to the relatively subtle changes observed here, we have previously shown that a defect in heme synthesis causes a dramatic drop in mitochondrial respiration in *T*. *gondii* [64]. Thus, we conclude that *Tg*ATM1 is likely not implicated in heme synthesis, *i*.*e*., the trafficking of heme synthesis intermediates (protoporphyrinogen IX) into the mitochondrion.

In other organisms, ATM1 and its homologs are implicated in the export of sulfur-containing compounds from the mitochondrion to facilitate [Fe-S] synthesis in the cytosol [7, 65, 66]. Previous studies in *T*. *gondii* and other organisms have shown that disruption of [Fe-S] synthesis leads to destabilization and concomitant decreased abundance of [Fe-S] in the affected subcellular compartment [35, 67, 68]. Thus, to explore the function of *Tg*ATM1, we conducted quantitative proteomic analyses comparing parental DiCreU1 and *Tg*ATM1-Ty-U1 parasites under untreated conditions or after 72 hours of Rapa treatment (**S5 Table**). In *Tg*ATM1-Ty-U1 parasites subjected to 72 hours of Rapa treatment, 277 proteins showed significant downregulation, while 60 proteins exhibited significant upregulation compared to equally treated DiCreU1 parasites (p <0.05 and >1.5-fold change) (**Fig 3E**). Conversely, in *Tg*ATM1-Ty-U1 parasites, exposed to Rapa for 72 hours, 11 proteins displayed significant decrease, whereas 107 proteins showed significant increase, compared to untreated *Tg*ATM1-Ty-U1 parasites (**Fig 3F**). To exclude non-specific effects caused by the strain or Rapa, we only considered proteins that exhibited significant changes in both comparisons. Consequently, the list was narrowed down to 15 upregulated and five downregulated proteins (**Fig 3G**). Notably, *Tg*ATM1 itself was not among the significantly altered proteins as it was not detected in all replicates but exhibited a clear downregulation upon Rapa treatment (**S5 Table)**. Assessing the subcellular localization [38] and performing GO-term enrichment analysis on the significantly altered proteins, revealed no specific impact of *Tg*ATM1 downregulation, with diverse subcellular compartments (**Fig 3G, S5 Table**) and biological processes affected (**S5 Table**). However, of the 20 significantly altered proteins, three were [Fe-S] proteins, including a NEET protein homolog, here termed CDGSH1, which has been implicated in transferring [Fe-S] to cytosolic proteins at the outer mitochondrial membrane in mammalian cells [69–73] (**Fig 3E and 3F**). Considering that *T*. *gondii* expresses approximately 64 [Fe-S] proteins [33], the presence of three in this shortlist of 20 proteins constitutes an overrepresentation. This led us to specifically assess the impact of *Tg*ATM1 downregulation on the abundance of [Fe-S] proteins, exploiting a previously published list [33]. In addition to the three significantly altered proteins (p <0.05 and >1.5-fold change), five others changed statistically significantly, but did not meet the 1.5-fold change threshold (**S5 Table)**. Notably, the total of five [Fe-S] proteins downregulated in response to *Tg*ATM1 depletion were exclusively of nuclear localization based on the LOPIT dataset, and included ABCE1, the delta catalytic subunit of the DNA polymerase (POLD1) and two subunits of the RNA polymerase (POLR1), all of which were previously proposed to be downregulated in response to disruption of the CIA pathway through downregulation of *Tg*NBP35 or *Tg*ATM1 [35, 36]. We speculate that a more striking fold-change and a global impact on cytosolic [Fe-S] was not observed here, due to the relatively short time of downregulation (72 hours). Comparable studies have assessed the abundance of cytosolic [Fe-S] proteins at later time points of depletion of the protein of interest (four days) and/ or used a faster system of downregulation (based on the western blots presented in those studies) [35, 36].

To assess the function of ATM1 in *P*. *falciparum*, we tested whether depletion of *Pf*ATM1 causes changes in mitochondrial iron and glutathione levels. Total organelle fractions from *Pf*ATM1-V_m_-treated and control parasites were prepared by treatment with low concentration of digitonin. The organellar pellet contained mitochondrial, apicoplast and low levels of nuclear marker proteins, and was free of the cytosolic marker tubulin (**S3H and S3I Fig**). At 108 hours post-treatment, the organellar fractions from *Pf*ATM1-V_m_-treated cells exhibited a notable rise in iron content compared to untreated controls **(S3J Fig).** While total glutathione levels showed a slight increase in treated cell fractions compared to controls, the difference was not statistically significant (**S3K Fig**). Accumulation of organellar iron was observed upon the knockdown of *Pf*ATM1; this accumulation could be due loss of [Fe-S] export via ATM1, although contributions from other phenomena are possible, such as increased uptake of iron into the mitochondrion stimulated by the decrease in extra-mitochondrial Fe-S proteins.

### *Tg*ATM1 depletion is rescued by homologs from *Plasmodium* and Yeast, and *Tg*ATM1 overexpression reduces NEET protein levels

**Fig 4:**
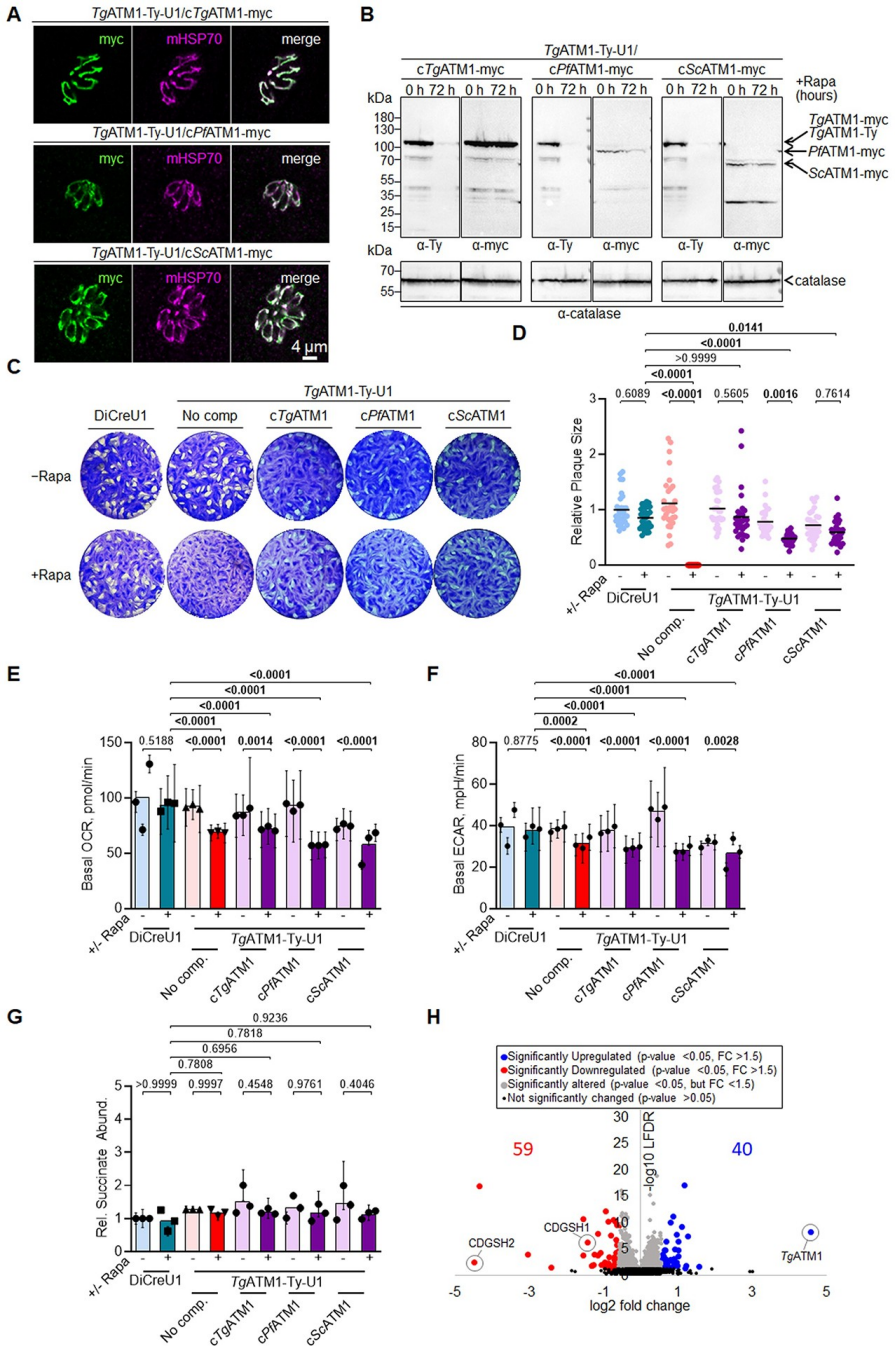
*Tg*ATM1-depleted *Toxoplasma* are functionally complemented through expression of the *Plasmodium* or yeast homolog. (**A**) Immunofluorescence assays of *Tg*ATM1-Ty-U1 parasites expressing a second copy of myc-tagged *Toxoplasma*- (*Tg*ATM1-Ty-U1/c*Tg*ATM1-myc), *Plasmodium*- (*Tg*ATM1-Ty-U1/c*Pf*ATM1-myc) or yeast ATM1 (*Tg*ATM1-Ty-U1/c*Sc*ATM1-myc). IFAs were stained with anti-myc antibody, as well as antibodies against a mitochondrial marker, mitochondrial heat shock protein (mHSP70). (**B**) Western blots of protein lysates from parasites as listed in (A) following 0 or 72 hours of downregulation of endogenous *Tg*ATM1 through addition of rapamycin (Rapa). Membranes were probed with anti-Ty and anti-myc antibodies as well as anti-catalase antibodies as loading control. The ATM1 proteins detected at varying molecular weights are indicated. The panel shows blots that were performed on the same membrane and exposed for identical durations. (**C**) Stained host cell monolayers revealing plaques formed by parental (DiCreU1), uncomplemented *Tg*ATM1-Ty-U1 parasites or *Tg*ATM1-Ty-U1 parasites expressing a second copy of TgATM1 as listed in (A) over seven days in presence or absence of Rapa. A-C: Representative images are shown from one of three independent biological replicates. (**D**) Plaque sizes quantified from assays as shown in (C) from three independent experiments. Sizes of individual plaques are shown relative to the mean of the parental line DiCreU1 not treated with Rapa. Means are indicated through horizontal bars. Conditions were compared using a One-way ANOVA followed by Tukey multiple pair wise comparisons. Significant differences (p <0.05) are highlighted in bold. (**E** and **F**) Basal oxygen consumption rate (OCR) (E) and basal extracellular acidification rate (ECAR) (F), as determined using an extracellular flux analyser following 0 or 72 hours of Rapa treatment of parasite lines listed in (C). (**G**) Relative abundance of the TCA cycle intermediate succinate, as determined by gas chromatography-mass spectrometry (GC-MS) of parental (DiCreU1) and *Tg*ATM1-Ty-U1 parasite extracts following 0 or 72 hours of Rapa treatment of parasite lines listed in (C). (E-G) The means and standard deviation of three independent biological experiments are shown. Bars represent the means of the three experiments. Conditions were compared using a One-way ANOVA followed by Tukey multiple pair wise comparisons. Significant differences (p <0.05) are highlighted in bold. Note that values for DiCreU1 and *Tg*ATM1-Ty-U1 parasites are as in Fig 3 C-H and were re-plotted. (**H**) Volcano plot highlighting changes in protein levels of *Tg*ATM1 overexpressing parasites (*Tg*ATM1-Ty-U1/c*Tg*ATM1-myc) compared to *Tg*ATM1-Ty-U1 parasites, both not treated with Rapa. Unchanged proteins are displayed in black, while significantly altered proteins are displayed in grey (< 1.5-fold change (FC), local false discovery rate, LFDR < 0.05) or in blue (>1.5-fold increased; LFDR < 0.05) or red (> 1.5-fold decreased; LFDR < 0.05). The number of significantly increased and decreased proteins is given and the NEET proteins CDGSH1 and CDGSH2 as well as *Tg*ATM1 are indicated.

The above functional analyses indicate that *Tg*ATM1 and *Pf*ATM1 may be implicated in the export of [Fe-S] from the mitochondrion to the cytosol, as previously described for the yeast homolog *Sc*ATM1 [11]. To provide more compelling evidence that *Tg*ATM1 and *Pf*ATM1 are indeed functional homologs of the well-characterized *Sc*ATM1, we generated *Tg*ATM1-Ty-U1 complemented cell lines which constitutively express *Sc*ATM1 4-myc tagged (c*Sc*ATM1-myc), in the uracil phosphoribosyltransferase (*UPRT*) locus [74] (**S4A Fig**). Functional complementation, similar to what was done with *Sc*ATM1, was also performed with *Pf*ATM1 4-myc tagged (c*Pf*ATM1-myc), along with a second copy of *Tg*ATM1 4-myc tagged (c*Tg*ATM1-myc) as a control. Transfected parasites were selected and cloned through limited dilution and correct integration was confirmed by PCR (**S4A and S4B Fig**). Products of the second copy ATM1 genes were efficiently detected and primarily localized to the mitochondrion of *T*. *gondii*, as confirmed by their co-localization with mHsp70 through IFA (**Fig 4A**). Effective downregulation of the endogenous *Tg*ATM1-Ty was verified following 72 hours of Rapa treatment in *Tg*ATM1-Ty-U1 parasites expressing the second copy ATM1s (**S4C Fig**). As for the IFA, western blot analysis confirmed that endogenous *Tg*ATM1 was efficiently downregulated upon addition of Rapa in the complemented strain and c*Tg*ATM1 was detected at the same size as the endogenous *Tg*ATM1-Ty. c*Sc*ATM1 migrated close to 70 kDa, as reported previously **(Fig 4B)** [23], and also exhibited a band at approximately 30 kDa, likely corresponding to a degradation product. On the other hand, c*Pf*ATM1-myc ran above the 100 kDa marker. This is in line with the size of the major *Pf*ATM1 band detected in the *P*. *falciparum* lysate (**Fig 1E**). The intensity of bands of c*Sc*ATM1-myc and c*Pf*ATM1-myc appeared to be weaker than c*Tg*ATM1-myc, indicating lower levels of expression (**Fig 4B**). To determine whether *Sc*ATM1 and *Pf*ATM1 function similarly to *Tg*ATM1 and whether their expression can rescue the severe phenotype linked to the depletion of endogenous *Tg*ATM1, we conducted plaque assays with the second copy complemented strains. All three strains formed plaques of lysis upon downregulation of endogenous *Tg*ATM1, confirming functional equivalence to *Sc*ATM1 and the genuine homology of *Pf*ATM1 to *Tg*ATM1 (**Fig 4C and 4D**). Notably, plaques formed by parasites complemented with the second copy of *Tg*ATM1-myc, following downregulation of endogenous *Tg*ATM1-Ty, were comparable to those formed by the parental line **(Fig 4C and 4D).** In contrast, parasites complemented with *Sc*ATM1-myc or *Pf*ATM1-myc formed slightly smaller plaques **(Fig 4C and 4D).** This difference could be due to lower expression levels **(Fig 4B)** or less efficient localization to the mitochondrion, or potentially a slight variation in substrate affinity between the ATMs of these species.

To evaluate the impact of expressing second copy ATMs on mitochondrial functions, we conducted extracellular flux analyses and GC-MS analyses as described earlier. Notably, the downregulation of endogenous *Tg*ATM1 resulted in a slight decrease in mitochondrial respiration and glycolytic activity, regardless of the presence of a second copy ATM (**Figs 4E, 4F, S4D and S4E, S3 Table**), despite the observed rescue in fitness (**Fig 4C and 4D**). These differences argue against a mitochondrial defect but rather suggest a general slight decrease in fitness upon downregulation of endogenous *Tg*ATM1. Concordant with the absence of mitochondrial defect upon expression of the second copy ATM1 proteins, we observed relatively modest or no differences in the abundance of TCA cycle intermediates and other mitochondrion-related metabolites, such as succinate (**Fig 4G, S4 Table**) in parasites expressing a second copy ATM1 and depleted in the endogenous protein or not. In summary, *Pf*ATM1 and *Sc*ATM1 serve as functional counterparts of *Tg*ATM1, capable of rescuing its depletion. The fact that expression of the second copy itself (*Sc*ATM1) or downregulation of the endogenous gene in second copy expressing strains (*Pf*ATM1) is associated with a decrease in plaques sizes emphasizes the necessity for precise regulation of ATM1 expression or subtle disparities in the transporters’ specific roles.

To expand on the relatively limited insights obtained through proteomic analyses of parasites depleted in *Tg*ATM1, we additionally performed quantitative proteomic analyses of parasites overexpressing *Tg*ATM1 (*Tg*ATM1-U1-Ty/c*Tg*ATM1-myc). Quantifying the abundance of proteins in *Tg*ATM1-U1-Ty/c*Tg*ATM1-myc parasites compared to *Tg*ATM1-U1-Ty parasites, both not treated with Rapa, revealed 59 proteins to be significantly decreased and 40 significantly increased (p <0.05 and >1.5-fold change) in parasites overexpressing *Tg*ATM1 **(Fig 4H, S6 Table)**. The most significantly upregulated protein was *Tg*ATM1 itself, exhibiting a 21-fold increase in its abundance. As observed during *Tg*ATM1 downregulation, *Tg*TAM1 overexpression impacted on proteins in various subcellular compartments and of diverse functions **(S6 Table)**. Strikingly, amongst the significantly downregulated proteins were two [Fe-S] proteins, here termed CDGSH1 and CDGSH2, which belong to the NEET family **(Fig 4H, S6 Table)**. These exhibited a 2.4-fold and 20.2-fold decrease in abundance, respectively, upon TgATM1 overexpression. In mammalian cells, CDGSH1 and 2 have been shown to localise to the outer mitochondrial membrane and endoplasmic reticulum-mitochondria interface, respectively, where they donate their uniquely coordinated and relatively labile [Fe-S] to other proteins [69–73]. CDGSH1 was also amongst only 20 significantly altered proteins upon *Tg*ATM1 downregulation (**Fig 3E-3G, S5 Table**), where it was upregulated (1.85-fold, average), suggesting that its abundance is inversely correlated to that of *Tg*ATM1.

### *Pf*ATM1 and *Tg*ATM1 form homodimers

**Fig 5:**
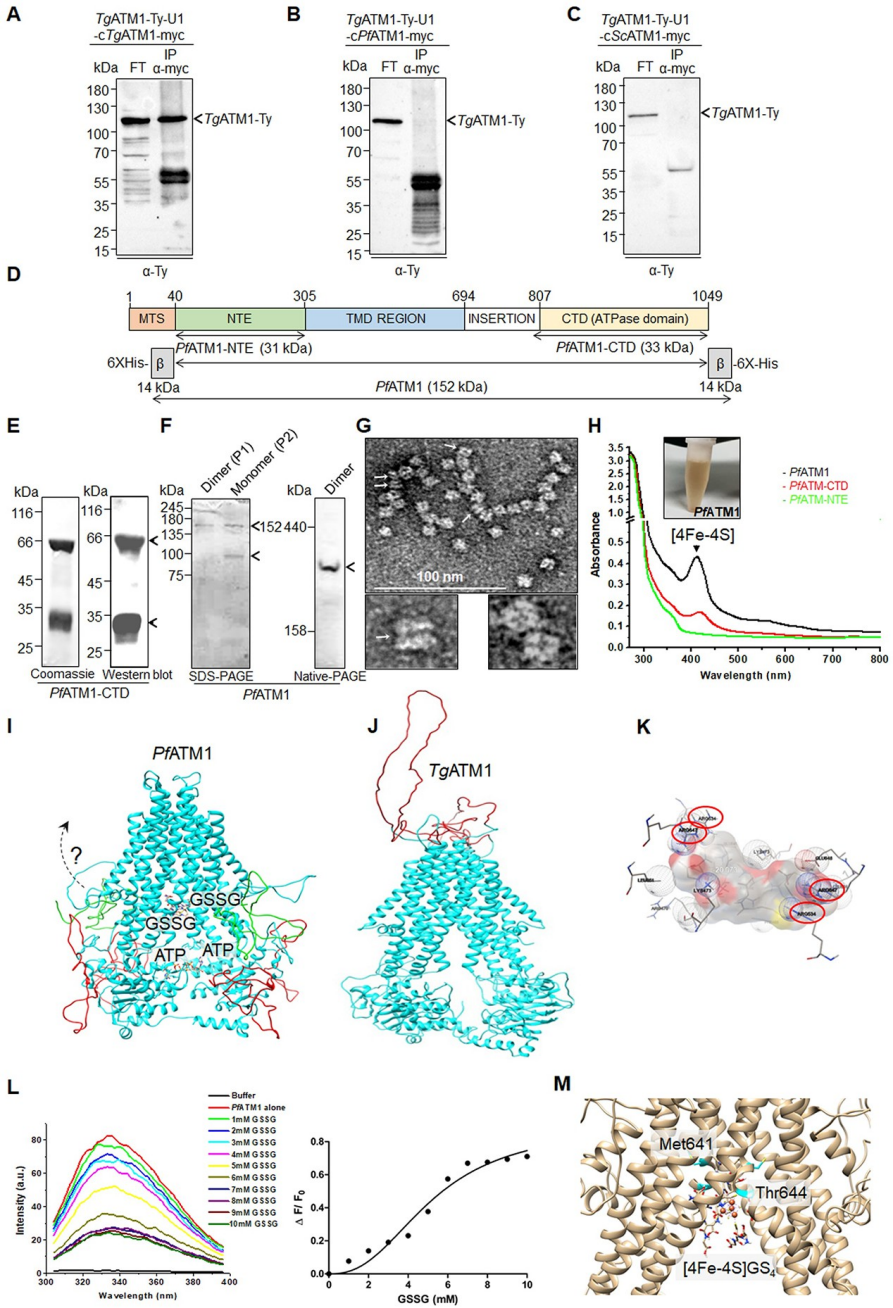
*Tg*ATM1 an *Pf*ATM1 form homodimers but no cross-species dimers are observed. (**A**-**C**) Western blots with material from an anti-myc co-immunoprecipitation assay (Co-IP) and the corresponding flow through (FT) from parasite lysates of *Tg*ATM1-Ty-U1 parasites expressing a second copy of myc-tagged *Toxoplasma*- (*Tg*ATM1-Ty-U1/c*Tg*ATM1-myc), *Plasmodium*- (*Tg*ATM1-Ty-U1/c*Pf*ATM1-myc) or yeast ATM1 (*Tg*ATM1-Ty-U1/c*Sc*ATM1-myc). Membranes were probed with anti-Ty antibody. *Tg*ATM1-Ty is indicated. Blots shown are representative of three independent experiments. (**D**) Schematic representation of *Pf*ATM1 domains. Full-length *Pf*ATM1 was expressed as a fusion protein in β-pET28a(+)-β. (**E**) Purified *Pf*ATM1-CTD (~30 kDa) with a dimeric form (~60 kDa) seen in Coomassie-stained SDS-PAGE, which are also detected in western blot with anti-6XHis Ab. (**F**) Purified dimeric and monomeric forms of *Pf*ATM1 after size exclusion chromatography separated on SDS-PAGE. *Pf*ATM1 dimer, of expected size 304 kDa, migrated between 158 and 440 kDa on native PAGE. (**G**) Negative staining TEM detected *Pf*ATM1 dimers. Arrows indicate electron densities revealing two-fold symmetry. (**H**) UV-VIS spectra of *Pf*ATM1, *Pf*ATM1-CTD and *Pf*ATM1-NTE. *Pf*ATM1 purified as a reddish-brown protein (inset). (**I**) and (**J**) *Pf*ATM1 (I) and *Tg*ATM1 (J) modelled on *Na*ATM1 (PDB:4MRS). The 128 aa insertion in between the transmembrane region and CTD of *Pf*ATM1 and the insertion between TM1 and TM2 of *Tg*ATM1 could not be modelled and are shown in red. A portion (aa 231–305) of the unique *Pf*ATM1 N-terminal extension is in green. An ATP molecule is docked in the ATPase domain (CTD) of each chain and two GSSG molecules are docked at the *Pf*ATM1 dimer interface in the transmembrane region. (**K**) 3-D interaction plot of two GSSG molecules positioned between the two *Pf*ATM1 chains. Arg534 and Arg647 (circled in red) in each *Pf*ATM1 chain have electrostatic interactions with the two GSSG molecules. **(L)** Change in intrinsic tryptophan fluorescence of *Pf*ATM1 in the presence of increasing concentrations of GSSG. a.u., fluorescence intensity in arbitrary units. In the corresponding graph, change in fluorescence intensity (ΔF/F_0_) at 335 nm is plotted against GSSG concentrations with curve-fitting using GraphPad Prism 5. (**M**) [4Fe-4S]GS_4_ manually docked into the central cavity of *Pf*ATM1 modelled on *Ct*ATM1 (PDB:7PRO).

It is well-established that *Sc*ATM1, ABCB7, and *At*ATM3 form homodimers [75–77]. To verify that *Tg*ATM1 forms homodimers, we performed a co-immunoprecipitation assay (Co-IP) with *Tg*ATM1-Ty-U1-c*Tg*ATM1-myc using an α-myc antibody (**Fig 5A**). The potential for cross-species heterodimer formation between *Tg*ATM1-Ty and the expressed *Sc*ATM1-myc and *Pf*ATM1-myc in *Tg*ATM1-Ty-U1 strains could have a notable impact on the functional characterization described above (**Fig 4**). This hypothesis was tested by performing Co-IPs with *Tg*ATM1-Ty-U1/c*Pf*ATM1-myc (**Fig 5B**) and *Tg*ATM1-Ty-U1/c*Sc*ATM1-myc (**Fig 5C**). The absence of *Tg*ATM-Ty in the pull-down assays suggests that no cross-species heterodimers are formed between the different ATMs even if myc-tagged ATM1 is readily detectable in the lysate (**S5A-S5C Fig**).

Dimerization of *Pf*ATM1 was tested using recombinant protein. *Pf*ATM1-CTD (30 kDa) was expressed as a 6X-His tagged protein in *E*. *coli* and purified through affinity chromatography (**Fig 5D and 5E**). *Pf*ATM1-CTD formed homodimers which did not completely break in SDS-PAGE but could be broken with urea (**S5D Fig**). Since stable full-length *Pf*ATM1 could not be expressed as a simple 6X-His fusion in bacteria, expression of soluble protein was accomplished by using the *E*.*coli* YbeL hydrophilic fusion domain (β) and 6XHis-tags fused to the N- and C-termini [78, 79] (**Fig 5D**). *Pf*ATM1 monomers (152 kDa) and dimers (~300 kDa) were separated by size exclusion chromatography (SEC) (**Figs 5F and S5E**) and a band of ~300 kDa was detected in the dimer peak in native-PAGE (**Fig 5F**), indicating that *Pf*ATM1 forms dimers in solution. The *Pf*ATM1 monomer fractionated with additional degradation product of ~100 kDa (**Fig 5F**). CD spectra of purified dimeric *Pf*ATM1 was as expected for a protein with predominantly alpha-helical secondary structural elements (**S5F Fig**). Negative stain transmission electron microscopy (TEM) confirmed its homodimeric state in aqueous buffer (**Fig 5G**). The purified complexes were observed in different orientations in the TEM micrographs and the dimers were clearly visible from the side view projections of the complex (**Fig 5G**). Here, the electron densities appear as two stripes or transverse striations revealing a clear two-fold symmetry which indicates that the complex is a dimer. *Pf*ATM1 purified as a reddish-brown protein suggesting the presence of bound iron (**Fig 5H**, inset). UV-VIS scan showed an intense peak at 420 nm indicating the presence of bound [4Fe-4S] (**Fig 5H**). Additional peak at 456 nm and shoulder at 322 nm expected for [2Fe-2S] were not seen. A lower intensity [4Fe-4S] peak was also observed in *Pf*ATM1-CTD, and none in *Pf*ATM1-NTE (**Fig 5H**).

Structure homology models of *Pf*ATM1 (231–1049 aa) and *Tg*ATM1 (410 to 1133 aa) were generated on the crystal structure of bacterial *Na*ATM1 (PDB id: 4MRS) [56]. Ramachandran plot analysis of the *Pf*ATM1 model indicated that 87% residues were in the favoured region, 10% residues in the additional allowed region, 1.9% residues in the generously allowed region and 1.1% residues in the disallowed region with RMSD score of 0.961 Å. The *Tg*ATM1 model had 89.7% residues in the favoured region, 7.8% residues in the additional allowed region, 1.2% residues in the generously allowed region and 1.3% residues in the disallowed region, with RMSD score of 0.755 Å. Only a part of the unique NTE of *Pf*ATM1 could be modelled and insertions in *Tg*ATM1 and *Pf*ATM1 appeared as unstructured loops. The full-length *Tg*ATM1 and *Pf*ATM1 structures available in the AlphaFold2 Protein Structure Database [80] also represent these stretches as extruded loops with some α-helices. Structural folds in all major domains were largely conserved (**Figs 5I, 5J, S6A and S6B**). Since *Pf*ATM1 has observed and predicted glutathione binding sites, the interaction of GSSG to the central cavity was tested in silico by simultaneously docking two molecules of GSSG in the *Pf*ATM1 model (binding affinity or free energy of binding = -20.073 kcal/mol) (**Figs 5K, S6C and S6D**). The predicted binding affinity of GSSG to *Pf*ATM1 compares well with re-dock of two GSSG molecules on the *Na*ATM1 co-crystal structure (binding affinity of -17.9 kcal/mol) and simultaneous docking of two GSSG molecules on the analogous site in *Ct*ATM1 (binding affinity of -20.59 kcal/mol). Out of the conserved arginine residues for glutathione binding in bacterial, fungal and yeast homologs, two *Pf*ATM1 arginine residues (Arg534, Arg647 downstream of TM4 and TM5, respectively) in each chain have electrostatic interactions with GSSG (**Figs 5K and S6D**). Two ATP molecules (one in each CTD) were also simultaneously docked in the *Pf*ATM1 model with a docking score of -25.465 kcal/mol in the best docking pose (**Fig 5I**). Molecular dynamics simulation confirmed the stability of *Pf*ATM1 with two GSSG molecules and two ATP molecules (**S6C Fig**). The binding of GSSG to *Pf*ATM1 was confirmed by spectrofluorometric analysis which showed a marked GSSG concentration-dependent quenching of intrinsic tryptophan fluorescence indicative of conformational changes upon interaction (**Figs 5L and S6E**). *Na*ATM1 has been shown to undergo conformational changes in transmembrane helix 6 (TM6) which modify the glutathione-binding site [56]. The altered TM6 conformation pre- and post-ATP hydrolysis is directly coupled to preclusion of glutathione binding and sets the directionality of transport. Although transmembrane domain prediction did not identify TM6 in *Pf*ATM1, the corresponding region in the molecular structure model superimposed on *Na*ATM1 TM6 (**S6A Fig**) suggesting a conserved role in GSSG binding and transport.

The possibility of a negatively charged [4Fe-4S]GS_4_ complex interacting with the positively charged central cavity of *Pf*ATM1 (**S6F Fig**) was addressed by modelling the protein on the recently available cryo-EM structure of the inward open configuration of fungal *Chaetomium thermophilum* ATM1 (*Ct*ATM1) (PDB: 7PRO) which binds [2Fe-2S]GS_4_ [17]_._ The size of the central cavity of *Pf*ATM1 was predicted to be >9000 Å, as compared to >7000 Å in *Ct*ATM1. [4Fe-4S]GS_4_ was manually docked into the *Pf*ATM1 cavity (**Fig 5M**). The gatekeeper residues Phe396 and Ser399 in *Ct*ATM1 (Met317 and 320 in *Na*ATM1) align with Met641 and Thr644 in *Pf*ATM1 (**S1B Fig**) and occupy a position above the central cavity (**Fig 5M**). MD simulation indicates RMS (root mean square) fluctuation of >1 Å in the putative gatekeeper residues in *Pf*ATM1 suggesting conserved function (**S6G Fig**). Structure modelling and docking results indicate that *Pf*ATM1 likely can accommodate GSSG or glutathione-complexed [4Fe-4S].

### *Pf*ATM1 interacts with components of the mitochondrial ISC and cytosolic CIA pathway

**Fig 6:**
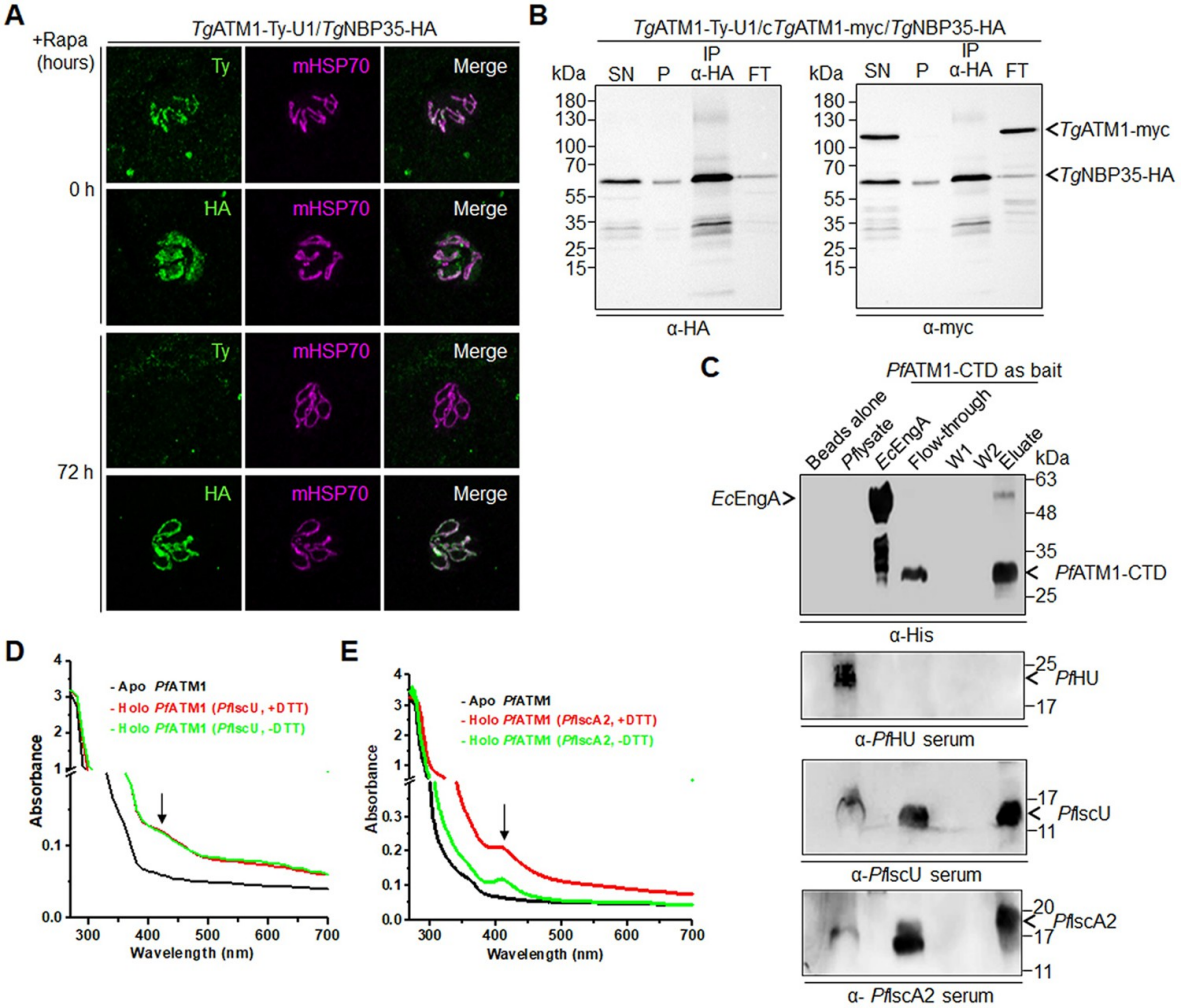
*Pf*ATM1 but not *Tg*ATM1 interacts directly with components of the cytosolic CIA pathway. (**A**) Immunofluorescence assay of *Tg*ATM1-Ty-U1/*Tg*NBP35-HA parasites following 0 and 72 hours of rapamycin (Rapa) treatment. Parasites were stained with anti-Ty and anti-HA antibodies as well the mitochondrial marker mitochondrial heat shock protein (mHSP70). (**B**) Western blots with material from an anti-HA co-immunoprecipitation assay (Co-IP) and the corresponding supernatant (SN), pellet (P) and flow through (FT) from lysates of *Tg*ATM1-Ty-U1/c*Tg*ATM1-myc/*Tg*NBP35-HA parasites. Membranes were probed with antibodies against HA and myc. *Tg*ATM1-myc and *Tg*NBP35-HA are indicated. Images shown in A and B are representative of three independent biological replicates, (**C**) Pull-down from *P*. *falciparum* lysate using *Pf*ATM1-CTD as bait. Anti-6XHis antibodies were used to detect *Pf*ATM1-CTD; anti-*Pf*IscA2, anti-*Pf*IscU and anti-*Pf*HU sera detected corresponding proteins in western blots. Eluates from negative control *Ec*EngA-tagged beads and beads alone were also loaded. (**D**) and (**E**) In vitro transfer of [4Fe-4S] to recipient apo-*Pf*ATM1 from *Pf*IscU (D) and *Pf*IscA2 (E) in the presence or absence of DTT.

NBP35 is a scaffold protein, important for [Fe-S] assembly in the CIA pathway [81]. In *T*. *gondii*, *Tg*NBP35 (TGGT1_280730) was localized to the outer mitochondrial membrane [35]. Thus, we speculated that a direct interaction between *Tg*ATM1 and *Tg*NBP35 might occur. To test this, an HA-tag was fused to the C-terminus of *Tg*NBP35 via CRIPR Cas9 editing at the endogenous locus of *Tg*NBP35 in *Tg*ATM1-Ty-U1 and *Tg*ATM1-Ty-U1/c*Tg*ATM1-myc parasites, thus generating *Tg*ATM1-Ty-U1/*Tg*NBP35-HA and *Tg*ATM1-Ty-U1/c*Tg*ATM1-myc/*Tg*NBP35-HA lines. These lines were used to establish by IFA that mitochondrial localization of NBP35-HA was not altered through downregulation of *Tg*ATM1 (**Fig 6A**). Also, co-IP with α-HA antibody did not pull down second copy expressed *Tg*ATM1-myc in *Tg*ATM1-Ty-U1/c*Tg*ATM1-myc/*Tg*NBP35-HA parasites **(Fig 6B**). Hence, we conclude that no stable interaction occurs between *Tg*NBP35 and *Tg*ATM1.

Similarly, to investigate the interaction between *Pf*ATM1 and components of the cytosolic CIA machinery, pull-down assays were carried out. Since the mitochondrial matrix side of *Pf*ATM1 comprises the CTD, *Pf*ATM1-CTD bound to Ni-NTA beads was used as bait to pull down interacting proteins from the parasite lysate. Western blots showed that *Pf*ATM1-CTD could pull down both *Pf*IscU and *Pf*IscA2 (**Fig 6C**). No signals for these proteins were seen in eluates of control sets using blank beads or unrelated protein *Ec*EngA as bait. Recombinant *Pf*IscU and *Pf*IscA2, expressed as GST fusion proteins and thrombin-cleaved to remove the GST tag (**S7A Fig**) were chemically reconstituted. Reconstituted *Pf*IscU and *Pf*IscA2 carrying [4Fe-4S] were then used as cluster donors for *Pf*ATM1 in transfer reactions in vitro, in the presence or absence of DTT (**Fig 6D and 6E**). Apo-*Pf*ATM1 could receive [4Fe-4S] from both donor proteins and was converted to the holo-form even in the absence of DTT, thus confirming its role as a recipient of [4Fe-4S] from ISC pathway cluster carriers.

To probe whether *Pf*ATM1 can interact with components of the CIA pathway to transport [4Fe-4S], *P*. *falciparum* NBP35 and CFD1 homologs were recombinantly expressed in *E*. *coli* (**S7B-S7E Fig**). NBP35 and CFD1 are ATPases that can exist as homodimers, heterodimers and also form a heterotetrameric [Fe-S] scaffold complex as part of the CIA machinery in yeast [9, 82]. NBP35 is annotated in PlasmoDB, and a *P*. *falciparum* CFD1 homolog is currently annotated as HCF101. Both proteins show conservation of major ATP binding and hydrolysis motifs as well as a CXXC motif each for binding [Fe-S] (**S8 Fig**). Antibodies generated against *Pf*NBP35 in rabbit recognized a specific band of the expected size of ~51 kDa in the parasite lysate (**S7I Fig**). The interaction of full-length *Pf*ATM1 with *Pf*NBP35 and/or *Pf*CFD1 in vitro was checked by incubating the recombinant proteins and resolving the complexes by native PAGE. *Pf*NBP35 and *Pf*CFD1 mixed in equimolar ratios formed a complex migrating above the 240 kDa marker protein, close to the expected heterotetrameric complex size of ~200 kDa (**S7F Fig**). The *Pf*ATM1 dimer formed a complex with *Pf*NBP35 as evident from an upward shift in migration in native PAGE, but did not interact with *Pf*CFD1 alone (**S7G Fig**). When the pre-formed *Pf*NBP35-*Pf*CFD1 heterotetramer was incubated with *Pf*ATM1, a complex migrating between 669 and 440 kDa was obtained (**S7G Fig**). This likely represents a complex formed by interaction of *Pf*ATM1 dimer and heterotetrameric *Pf*NBP35-*Pf*CFD1 with an expected total size of ~500 kDa. To check whether *Pf*ATM1-NTE which might be exposed in the mitochondrial intermembrane space can interact with *Pf*NBP35, in vitro complex formation was checked with purified *Pf*ATM1-NTE or -CTD (**S7H Fig**). An interaction was detected with *Pf*NBP35 and *Pf*ATM1-NTE (**S7H Fig,** lane 4), but not with *Pf*ATM1-CTD (**S7H Fig**, lane 5).

*Pf*ATM1-NTE was next used as bait in pull-down experiments. Western blot confirmed that *Pf*ATM1-NTE specifically pulled down *Pf*NBP35 from the parasite lysate (**S7J-S7K Fig**). *Pf*NBP35 was not pulled down with beads alone or with *Pf*EngA as bait. The results suggest that *Pf*ATM1 can interact with the cytosolic CIA component *Pf*NBP35 which is part of the *Pf*NBP35-*Pf*CFD1 scaffold complex and that its unique NTE can mediate this interaction in vitro. The orientation of the *Pf*ATM1-NTE domain in relation to the inner mitochondrial membrane is unclear. The NTE contains a hydrophobic stretch from aa 154 to 163 which is part of a predicted α-helix (aa 135 to 163) (**S1B Fig**); if this region traverses the membrane, a part of the NTE could be exposed to the intermembrane space. However, in the absence of direct interaction data with parasite proteins *in situ*, the physiological relevance of the *Pf*ATM1-*Pf*NBP35 interaction remains to be established.

### ATP hydrolysis is stimulated by GSSG and [4Fe-4S] and enhances GSSG transport by *Pf*ATM1

**Fig 7:**
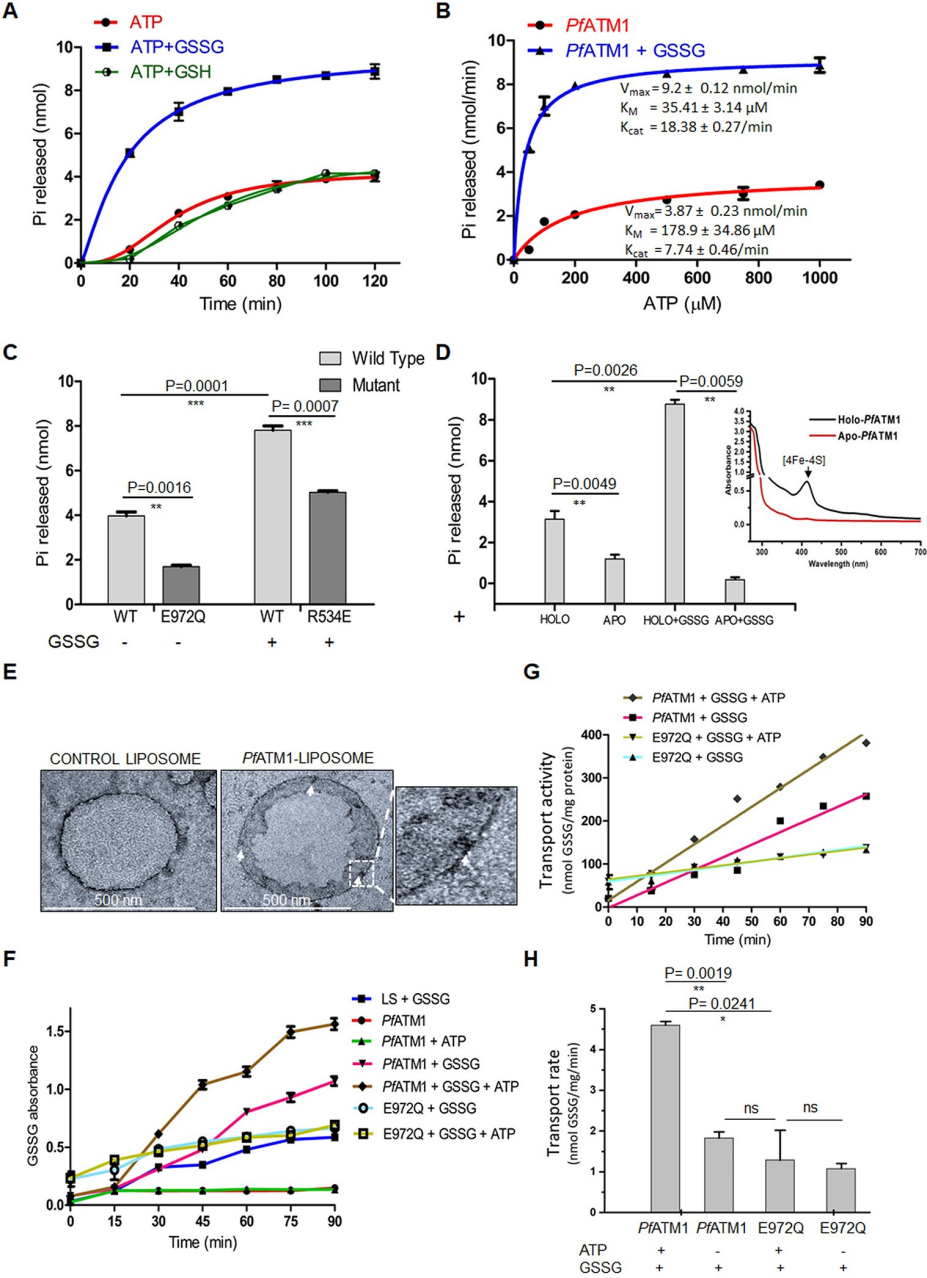
ATPase activity of *Pf*ATM1 and GSSG transport by *Pf*ATM1-proteoliposomes. (**A**) ATP hydrolysis by *Pf*ATM1 in the presence of GSH or GSSG as measured by Pi release. (**B**) Kinetics of ATP hydrolysis by *Pf*ATM1 in the presence or absence of GSSG. (**C**) Comparison of basal ATPase activity of the wild-type and *Pf*ATM1-E972Q mutant. ATP hydrolysis by *Pf*ATM1–R534E mutant was compared with wild-type protein in the presence of GSSG. (**D**) Comparison of ATPase activity of holo- and apo-*Pf*ATM1 (inset) in the presence or absence of GSSG. (**E**) Negative staining TEM of control liposomes and *Pf*ATM1-proteoliposomes. Incorporated protein is indicated by arrows. A section of the proteoliposome with *Pf*ATM1 dimer is enlarged. (**F**) Time-dependent GSSG uptake in the presence or absence of ATP by proteoliposomes carrying *Pf*ATM1-wild type or *Pf*ATM1-E972Q. LS, control liposome without protein. (**G**-**H**) Transport activity (G) and transport rate (H) were calculated. P values (t-test) from two biological replicates are indicated.

The ATPase activity of *Pf*ATM1 and its modulation by different ligands was assayed. We first determined that the ATPase activity of the protein was time- and concentration-dependent (**S9A and S9B Fig)**, followed by evaluation of the effect of oxidized and reduced glutathione (GSSG and GSH) on ATP hydrolysis. Significant enhancement of ATPase activity was observed only in the presence of GSSG (P = 0.001) (**Fig 7A**). Kinetics of ATP hydrolysis showed that GSSG-bound *Pf*ATM1 had lower K_M_ (greater affinity for ATP) and higher V_max_ (**Fig 7B**). The catalytic efficiency of *Pf*ATM1 (K_cat_/K_M_) increased ~11-fold in the presence of GSSG (0.52 μM^-1^ min^-1^ versus 0.043 μM^-1^ min^-1^ in the presence and absence of GSSG, respectively) indicating conformational changes transmitting to the ATPase domain upon GSSG binding to its cognate site in the transmembrane region.

The specificity of ATPase activity by purified *Pf*ATM1 was confirmed by generating the Walker B ATP hydrolysis mutant E972Q (**Fig 7C**). Mutation of the GSSG-binding site Arg534 identified in GSSG-*Pf*ATM1 docking **(Fig 5**) also significantly reduced ATP hydrolysis in the presence of GSSG (**Fig 7C**). Since *Pf*ATM1 strongly bound [4Fe-4S], the effect of the cluster on the ATPase activity was assayed by comparing apo- and holo-forms of the protein (**Fig 7D**). Removal of the cluster caused a significant reduction in ATP hydrolysis which could not be restored by addition of GSSG, suggesting that enhancement of ATPase activity by GSSG is contingent upon the presence of [4Fe-4S]. This was corroborated by spectrofluorometric analysis of conformational changes upon GSSG interaction with apo-*Pf*ATM1 (**S9C Fig**). Unlike holo-*Pf*ATM1 (**Fig 5L**), there was no drop in fluorescence at low GSSG concentrations; the drop seen at 3 mM GSSG did not increase further at higher concentrations. The results suggest that GSSG-induced conformational changes in *Pf*ATM1 are more prominent in the presence of [4Fe-4S]. Prior presence of the cluster might hold *Pf*ATM1 in a conformation that allows GSSG easier access to the central cavity.

To analyse whether *Pf*ATM1 can transport GSSG as reported for bacterial, yeast and plant ATM homologs [12, 56], we incorporated *Pf*ATM1 and *Pf*ATM1-E972Q in unilamellar proteoliposomes. The average diameter of *Pf*ATM1-liposomes deduced by DLS was ~312 nm (**S9D Fig**). Protein incorporation was confirmed by negative stain TEM (**Fig 7E**) and SDS-PAGE (**S9E Fig**). GSSG uptake by *Pf*ATM1-proteoliposomes was clearly enhanced by ATP (**Fig 7F**). The effect of ATP hydrolysis on GSSG transport was confirmed by the fact that *Pf*ATM1-E972Q proteoliposomes functioned identically in the presence or absence of ATP. Low levels of background GSSG uptake seen with blank liposomes is possibly attributable to limited diffusion across the membrane (**Fig 7F)**. Transport activity was linear with time (**Fig 7G**). The maximum transport rate of GSSG in the presence of ATP was 4.6 ± 0.09 nmol GSSG mg^-1^ min^-1^. The transport rate was halved to 1.83 ± 0.14 nmol GSSG mg^-1^ min^-1^ when ATP was not added to the reaction (**Fig 7H**). *Pf*ATM1-E972Q showed no significant change in transport rate of GSSG with or without ATP.

## Discussion

The conservation of an ATM1 homolog in *P*. *falciparum*, *T*. *gondii* and other apicomplexan parasites, along with its mitochondrial localization, implies its role as a transporter linking the parasite’s mitochondrial ISC and CIA systems. The presence of ATM1 in *Cryptosporidium* spp. which carry a mitosome but harbour ISC pathway proteins [83] also points to a significant role for mitochondrial [Fe-S] synthesis and its transport to the cytosol. Among apicomplexan parasites, *Pf*ATM1 and *Tg*ATM1 exhibit the longest unconserved N-terminal extension compared to other ATM1 homologs. Additionally, *Pf*ATM1 and *Tg*ATM1 feature unique insertions between specific regions, further distinguishing them from other homologs. *C*. *parvum* ATM1 (*Cp*ATM1) differs from other members in having a predicted 48-residue insertion between the D- and H-loop of the ATPase domain. Our results implicate *Pf*ATM1-NTE in mediating interaction with the CIA scaffold protein NBP35; the role of other insertions in protein conformational changes, ATP hydrolysis or protein-protein interaction for transport is unclear.

The structural framework underlying ATM1 transport function has been derived from X-ray crystallography and cryo-EM studies on bacterial *Na*ATM1, fungal *Ct*ATM1 and plant *At*ATM3 [17, 56, 77]. Different configurations are adopted during cargo transport; as the dimer progresses through the transport process, conformational changes are seen in TM6 and the glutathione/substrate-binding cavity of the inward facing and inward facing occluded structures in *Na*ATM1 [56]. The structural model for *Ct*ATM1 proposes that it accepts glutathione-complexed [Fe-S] cargo in an inward open, partially occluded state with the cargo interacting with residues in the inner cavity at the mitochondrial matrix interface [17]. The inner cavity in prokaryotic ATMs is more electroneutral, a property compatible with binding of metal ions or metal-GS complexes for heavy metal transport [56, 84]. On the other hand, eukaryotic ATMs such as *Ct*ATM1 have a highly positively charged inner cavity and can bind negatively charged [2Fe-2S]GS_4_. *Pf*ATM1 and *Tg*ATM1 also have a highly electropositive cavity that would allow binding of [4Fe-4S]GS_4_. Prokaryotic ATMs have two methionine residues at the inner gate (M317 and M320 in *Na*Atm1) which are replaced by phenylalanine and serine (or threonine) in eukaryotic mitochondrial homologs including *Tg*ATM1 (**S1B Fig**); *Pf*ATM1 retains the first methionine and *Cp*ATM1 retains the second methionine. The putative gatekeeper residues in *Pf*ATM1 are likely to follow the rearrangement seen in *Ct*ATM1 Phe396 and Ser399 during transition from the inward open/cluster structure to the inward open/occluded structure as proposed for the latter [17].

Binding of ATP in the NBD seems to trigger complete occlusion in *Ct*ATM1, and subsequent release of the cargo associated with outward facing configurations [77]. It has been suggested that a ternary complex with dimerized nucleotide binding domains and containing both the nucleotide and cargo exists only transiently [77]. ATP hydrolysis occurs post cargo release, thus allowing a new transport cycle [17]. Experiments with recombinant ATMs have shown that ATPase activity is stimulated by GSH and/or GSSG, by metal-GSH complexes as well as [2Fe-2S]GS_4_ [13, 16, 17, 84]_._ Stimulation of ATP hydrolysis is higher with GSSG than GSH in *Na*ATM1, *Sc*ATM1, *Ct*ATM1, and *At*ATM3 [12, 17, 84]; however, greater stimulation with GSH is also reported [77, 85]. *Pf*ATM1 ATPase activity is enhanced only in the presence of GSSG indicating preferential interaction of oxidized glutathione. This stimulation seems to require the prior presence of [4Fe-4S] as GSSG is unable to enhance ATP hydrolysis by apo-*Pf*ATM1. *Pf*ATM1 is an efficient transporter; Its ATP-dependent GSSG transport activity, as measured by its transport rate in proteoliposomes, is ~4.6 nmol/mg/min which is higher than the rate reported for *Na*ATM1 (1.5 nmol/mg/min) [56].

The physiological effects of *Pf*ATM1 downregulation include a ~6-fold increase in parasite organellar iron content, which likely reflects accumulation of iron in mitochondria, with no significant change in glutathione levels. This is in agreement with reported knockout of yeast ATM1 which caused a 30-fold increase in mitochondrial free iron with only a two-fold increase in glutathione [23]. In yeast, depletion of *Sc*ATM1 further caused changes in approximately 200 genes detected by DNA microarrays [24]. Iron responsive proteins, *Sc*Aft1p and *Sc*Aft2p (no homologs found in *T*. *gondii*) were upregulated due to the dysregulation of iron homeostasis; glycolytic enzymes were also upregulated. The latter was likely a direct response to the downregulation of heme enzymes, heme dependent proteins, and respiration proteins that affected the electron transport chain (ETC). In our proteomic analysis of *T*. *gondii*, significant changes in the abundance of 20 proteins were considered specific for the downregulation of *Tg*ATM1. Fifteen proteins were upregulated while five were downregulated. In contrast to the yeast DNA microarrays, we determined neither a specifically impacted pathway nor specific compartments affected by the depletion of *Tg*ATM1. Cytosolic and nucleus [Fe-S] proteins are involved in several pathways such as DNA replication and repair enzymes in the nucleus [86]. Pamukcu and colleagues published a list of 64 putative [Fe-S] proteins from which 26% are predicted to be activated by the CIA [33]. Using this published list, we identified three putative [Fe-S] proteins significantly changing upon *Tg*ATM1 depletion: TGGT1_305920, TGGT1_237560 and TGGT1_260870. In this relatively small set of changed proteins, three represent a considerable (~20-fold) enrichment of [Fe-S] proteins.

TGGT1_305920 was downregulated upon the depletion of *Tg*ATM1. It is the homolog of NTHL1 (ENSG00000065057), NTG1 (YAL015C), and NTH1 (AT2G31450) in human, yeast, and *A*. *thaliana*, respectively. They are highly conserved enzymes that catalyse the first step of base excision repair upon oxidative DNA damage [87]. These proteins are also known as endonuclease III, and the *P*. *falciparum* homolog has recently been characterized as a [4Fe-4S]-containing DNA glycosylase [88]. With a fitness score of -0.96, TGGT1_305920 is likely not essential for parasite survival. TGGT1_237560 (*Tg*ISU1) localizes to the mitochondrion and was previously characterized [33]. Interestingly, in yeast, it was also found that the scaffold proteins *Sc*ISU1 and *Sc*ISU2, two homologs of *Tg*ISU1, were upregulated upon downregulation of *Sc*ATM1; this was accompanied by downregulation of the carrier protein glutaredoxin 5 (*Sc*GRX5), homolog to the uncharacterized protein TGGT1_268730 [24]. For the authors, the upregulation of these proteins was likely triggered by the accumulation of the unknown substrate, which caused an increase of [Fe-S] formation in the mitochondria and transferred to the carrier protein to standby [24].

Finally, the third [Fe-S] protein altered in response to *Tg*ATM1 downregulation, TGGT1_260870, shares considerable similarity with human CDGSH1 (also MitoNEET, CISD1 or mNT) (ENSG00000122873) and NEET (AT5G51720) proteins in *A*. *thaliana*. While TGGT1_260870 was upregulated in cells depleted in *Tg*ATM1, it was downregulated alongside another NEET protein (TGGT1_255910) in cells overexpressing *Tg*ATM1. These findings strongly suggest an inverse correlation between the levels *Tg*ATM1 and these NEET proteins. Humans possess three NEET proteins (*Hs*CDGSH iron sulfur domain containing proteins 1–3), which share a 39 amino acid segment termed CDGSH domain and are encoded by CISD1-3. A [2Fe-2S] is coordinated by an unusual pH-labile 3-Cys and one non-Cys-ligand, which allows these proteins to transfer their [Fe-S] [69]. *Hs*CDGSH1 and *Hs*CDGSH2 (also NAF-1, miner1, ERIS and CISD2) are homodimers, with each subunit holding an [2Fe-2S] and anchored into the outer mitochondrial membrane or the endoplasmic reticulum-mitochondrion interface, respectively, via a single transmembrane domain [89]. Both proteins have been proposed to participate in the transfer of [2Fe-2S] at the outer mitochondrial membrane to cytosolic proteins [69–73, 89].

Similar to humans, *T*. *gondii* also expresses three NEET proteins, all of which have been localized to the mitochondrial membranes by LOPIT [38]. The *T*. *gondii* homologue of CDGSH3 (also HsMiNT, CISD3 and Miner2), *Tg*ApiCox13, was recently characterized and shown to be important for mitochondrial metabolism as a critical component of complex IV [90]. To the best of our knowledge, the other two NEET proteins, abundance of which appears to be dependent of *Tg*ATM1 levels, have not been functionally characterized in *T*. *gondii*. *Tg*CDGSH1 deletion was, however, shown to be dispensable *in vitro* and *in vivo*, while the epitope-tagged protein was not detected in a basic investigation of eight *T*. *gondii* zinc finger proteins [91]. The dispensability of *Tg*CDGSH1 and *Tg*CDGSH2 is also supported by their positive fitness score in a CRISPR-based genome wide fitness screen [39], which stands in sharp contrast to the low negative fitness score of *Tg*CDGSH3. This could indicate a dispensable function of *Tg*CDGSH1 and 2 or the two proteins may constitute a synthetic lethal pair with redundant activities. Given the observed inverse correlation between the abundance of *Tg*ATM1 and that of *Tg*CDGSH1 and 2, it is tempting to speculate that *Tg*ATM1 and the NEET proteins cooperate in the export of [Fe-S] from the mitochondrion, acting at the inner and outer mitochondrial membrane, respectively. The regulation of the NEET proteins in response to *Tg*ATM1 expression levels may present a mechanism to regulate [Fe-S] donation to cytosolic proteins during fluctuating [Fe-S] cluster export across the inner mitochondrial membrane. Alternatively, changes in the NEET proteins’ expression might be the result of altered iron levels in response to changing *Tg*ATM1 abundance or other unknown mechanisms. The precise mechanisms of [Fe-S] export and iron homeostasis in the mitochondrion of Apicomplexa must be further elucidated in future studies.

Considering the proteomic data and results from the successful functional complementation with the well-characterized yeast ATM1, our findings indicate that *Tg*ATM1 plays a role in [Fe-S] protein homeostasis. Two previous studies have investigated the effect of defects in the CIA pathway on the abundance of [Fe-S] proteins: Aw and colleagues showed that downregulation of *Tg*NBP35 causes a decrease in the abundance of the cytosolic [Fe-S] protein *Tg*ABCE1 [35]. Additionally, a recent study of *Tg*ATM1 reported that its downregulation resulted in the decrease of eight cytosolic [Fe-S] proteins, including the ribosome biogenesis factor, ABCE1, and POLD1, the [Fe-S] containing DNA polymerase delta subunit [36]. Importantly, in our proteomic analysis, ABCE1 and POLD1 were also significantly downregulated when comparing parasites depleted in *Tg*ATM1 to the parental line and non-depleted *Tg*ATM1-Ty-U1 parasites but the downregulation did not meet the 1.5-fold threshold. Notably, changes observed in the aforementioned studies were observed after 3–6 days of downregulation, in a system where the protein presumably acting in the CIA, namely *Tg*NBP35 and *Tg*ATM1, were downregulated within 1–2 days. Thus, the findings reported in these studies and here suggest that changes to the proteome through disruption of the CIA pathway occur relatively late.

Our functional analyses suggest that *Tg*ATM1 and *Pf*ATM1 harbour a role in parasite mitochondrial iron homeostasis and transfer of [Fe-S] clusters from the parasite mitochondrion to recipients in the cytosolic CIA machinery. *Pf*ATM1 can receive [4Fe-4S] generated through its ISC pathway from the scaffold and transfer protein *Pf*IscU as well as the transfer protein *Pf*IscA2. Since [4Fe-4S] can be directly assembled on *Pf*IscU [31], it is possible that *Pf*ATM1 has evolved to preferentially carry [4Fe-4S] over [2Fe-2S]. Although the *Pf*ATM1 inner cavity seems to be large enough to accommodate [4Fe-4S]GS_4_, it remains to be conclusively established if this is an actual cargo. The possibility of [4Fe-4S] alone, or a GSSG/GS-S-SG sulfur intermediate for tRNA thiolation [7, 12, 85] being transported via *Pf*ATM1 cannot be ruled out. It has been proposed that ATM1 transporters might recognise a common component in both mitochondrial [Fe-S] and sulfur intermediates; this common component could be a GSH derivative [7]. The predominant cluster generated by *T*. *gondii* ISC and the preferred cargo ([4Fe-4S]GS_4_ or [2Fe-2S]GS_4_) carried by *Tg*ATM1 is not known. The fact that both *Sc*ATM1, which has [2Fe-2S]GS_4_ as cargo [16], and *Pf*ATM1 can partially complement *Tg*ATM1 function suggests a degree of adaptability in the system. If [4Fe-4S] is the major cluster generated by *T*. *gondii* mitochondrial ISC, *Sc*ATM1 is possibly capable of transporting [4Fe-4S]GS_4_ complexes. On the other hand, if [2Fe-2S]GS_4_ is the cargo for *Sc*ATM1 in *T*. *gondii*, the conversion of [2Fe-2S] to [4Fe-4S] by reductive coupling of two adjacent [2Fe-2S] clusters might occur at a subsequent stage, perhaps in the CIA scaffold complex.

The connection between mitochondrial ISC and cytosolic CIA machineries for incorporation of [Fe-S] onto cytosolic apo-protein recipients in *P*. *falciparum* is made by the receipt of the *Pf*ATM1 cargo [4Fe-4S] by *Pf*NBP35. *Pf*NBP35 exists as a heterotetrameric scaffold complex with *Pf*CFD1 and would serve to transfer the labile [4Fe-4S] cluster to downstream CIA components. The proximity of *Pf*NBP35 to *Pf*ATM1 is suggested by studies on *T*. *gondii* NBP35 showing that the protein localizes to the outer mitochondrial membrane through a transmembrane region at its N-terminal end [35]. An N-terminal transmembrane region is also predicted in *Pf*NBP35. The presence of *Pf*NBP35 and/or the *Pf*NBP35-*Pf*CFD1 complex in the outer mitochondrial membrane would allow easy access to the released [4Fe-4S]-cargo from *Pf*ATM1.

In this study, we identify apicomplexan ATM1 as a highly conserved essential component that bridges the mitochondrial and cytosolic [Fe-S] pathways in these important human pathogens. Similar results were recently reported in another study [36]. We provide novel insights into the transporter’s function through detailed functional analysis and by revealing the apicomplexan transporter as a functional homolog of the well-characterized yeast ATM1. Detailed biochemical analyses identify [4Fe-4S]GS_4_ as a plausible cargo. Taken together, these findings add to previous studies delineating the metabolism of important human pathogens and provide important insights into the evolution of [Fe-S] synthesis pathways.

## Materials and methods

### Ethics statement

All animal experiments were conducted in compliance with guidelines of the Committee for Control and Supervision of Experiments on Animals (CCSEA), India. Approval was obtained from the Institutional Animal Ethics Committee of the Central Drug Research Institute (CDRI) (#IAEC/2007/126/Renew-10(234/17). Approval for use of RBCs from healthy human volunteers was obtained from the CDRI Institutional Ethics Committee (Human Research) (#CDRI/IEC/2017/A4); formal verbal consent was obtained from the volunteers.

### In-silico analyses of ABCB superfamily members and phylogeny

The *Plasmodium* and *Toxoplasma* databases (http://plasmodb.org; http://toxodb.org) were mined for sequences of putative members of ABCB superfamily in *P*. *falciparum* and *T*. *gondii*. Targeting predictions were made using PlasmoAP (https://plasmodb.org/plasmo/app/plasmoap; a predictor for the bipartite apicoplast-targeting sequence comprising signal and transit peptide), Signal P (http://cbs.dtu.dk/services/SignalP/), Mitoprot II (https://ihg.gsf.de/ihg/mitoprot.html) and TargetP-2.0 (http://www.cbs.dtu.dk/services/TargetP; used to predict presence of N-terminal mitochondrial transit peptide- mTP). TMHMM- 2.0 [92] was used to predict membrane spanning regions in the protein.

A maximum-likelihood phylogenetic genetic tree was constructed with 1000 bootstrap replicates using LG+R+F model by ATGC: SMS (Smart Model Selection) in PhyML 3. 0 [93]. A total of 30 protein sequences (**S1 Table**), including apicomplexan ATMs, were used for constructing the phylogenetic tree. Amino acid sequences were taken from NCBI database **(**www.ncbi.nlm.nih.gov**)** after BLASTp searches [94] for homologs of *Pf*ATM1 (*Pf*ABCB6) from bacteria to higher eukaryotes.

### Sequence analysis, molecular structure modelling and docking

Sequence alignment of *Pf*ATM1 with its homologs was done using Clustal Omega [95]. DISOPRED 3 (http://bioinf.cs.ucl.ac.uk/psipred/) was used to analyse the overall disordered regions in *Pf*ATM1. Molecular structure homology models of *Pf*ATM1 and *Tg*ATM1 were generated using Modeller 10.1 [96] based on the crystal structures of ATM1 of the bacterium *N*. *aromaticivorans* (PDB: 4MRS) [56] or the filamentous fungus *C*. *thermophilum* (PDB: 7PRO) [17]. 50 models were generated. The final model was selected according to DOPE (discrete optimized protein energy) score and the modeler objective function molpdf. PDBsum server [97] was used for external evaluation of the selected best model. Root mean square deviation (RMSD) score was estimated by alignment with template structure on PyMOL (The PyMOL Molecular Graphics System, Version 2.2, Schrödinger, LLC, 2018). Secondary structural analysis was done by Ramachandran plot statistics of the best selected model through RAMPAGE [98]. Vina version 1.2.0 [99] was used for simultaneous docking of two GSSG ligands around the analogous space in the *Pf*ATM1 model using Autodock4 forcefield. Two ATPs were simultaneously docked within a grid containing all putative ATP binding residues on each chain using Vina version 1.2.0 and Autodock 4 forcefield [100]. The docked complex consisting of protein, two GSSG molecules and two ATP molecules were used to perform molecular dynamics simulation using GROMACS version 2020.1 [101]. The simulation box was energy minimized, neutralized and equilibrated and subjected to molecular dynamics simulation using Charmm36 forcefield.

For interaction with glutathione-coordinated [4Fe-4S], *Pf*ATM1 was modelled on *Ct*ATM1 inward-open structure (PDB: 7PRO) [17]. A total of 50 models were generated and the best model was chosen on the basis of modeller objective function (molpdf). Cavity size was measured by CastP3.0 [102] with 2.5 Å probe. [4Fe-4S] was extracted from the pdb structure of [4Fe-4S]-dependent thiouracil desulfidase (PDB: 6Z92) [103]; 3D structure of glutathione was retrieved from PubChem (CID: 124886). The structure of glutathione was energy minimized using gaff forcefield [104] in Open Babel v3.0.0 by employing steepest descent algorithm with 1500 steps. Structures of a [4Fe-4S] and four molecules of glutathione were then joined as [4-Fe-4S]GS_4_. The final ligand was manually docked in the analogous pocket defined in the *Ct*ATM1 structure [17] using Chimera v1.6.

### Parasite strains and culture

*P*. *falciparum* 3D7 was cultured in human erythrocytes maintained in complete RPMI-1640 medium (Sigma-Aldrich, USA) supplemented with 0.5% (w/v) Albumax II (Invitrogen, Thermo Fisher Scientific, USA) and 0.2% sodium bicarbonate (Sigma-Aldrich, USA) at 2% hematocrit. Parasite genomic DNA was isolated by phenol-chloroform extraction after 0.05% saponin lysis of infected RBCs. Parasites were synchronized with 5% sorbitol at early- to mid-ring stage.

The *T*. *gondii* DiCre strain was a generous gift from Moritz Treeck. Parasites were maintained under standard tachyzoite growth conditions through regular passage in confluent human foreskin fibroblasts (HFFs) in Dulbecco Modified Eagle Medium (DMEM, Gibco, Thermo Fisher Scientific, USA) supplemented with 5% fetal bovine serum (FBS, Gibco, Thermo Fisher Scientific), 2 mM L-glutamine (Gibco, Thermo Fisher Scientific, USA) and 25 μg/ml gentamycin (Gibco, Thermo Fisher Scientific, USA). Cultures were maintained in humidified incubators at 37°C and 5% CO_2_.

### Vivo-morpholino (VMO) mediated *Pf*ATM1 knockdown

A specific Vivo-morpholino (*Pf*ATM1-V_m_; 5’-ACTCTCTATAATATTTCATCA*TG*ACA-3’) complementary to 5’UTR region of *Pf*ATM1 mRNA was designed and synthesized by Gene Tools (LLC, USA) and used as a translation blocking agent. 5’-AAGCCTAT*TG*CATAGAAAACGTACG-3’ was the non-specific control morpholino. *Pf*ATM1-V_m_ (3 μM) was added to late ring-stage synchronized *P*. *falciparum* 3D7 culture at 10% parasitemia and incubated at 37°C for 6 hours [61]. The morpholino was removed by media replacement. Parasite asexual blood stage development was monitored till 144 hours of parasite growth. After 72, 96 and 144 hours, parasites were harvested and *Pf*ATM1 expression was analysed by western blotting using anti-*Pf*ATM1 antibody. *Pf*ATM1 and actin levels were quantified by densitometry using ImageQuant TL software (Cytiva, USA). Parasite asexual growth stages were monitored by Giemsa staining.

Organellar fractions were prepared by treating parasites with low digitonin concentration (0.05%) as described previously [105, 106]. Total organellar fractions of morpholino-treated and untreated cells (from culture dishes containing synchronized parasites with equal parasitemia) were electrophoresed on 10–14% SDS-PAGE and western blotting was carried out with anti-α tubulin Ab and anti-histone 3 Ab (Sigma-Aldrich, USA). The blot was stripped and re-probed with anti-*Pf*HU Ab and anti-*Pf*IscA2 [31]. Iron in organellar fractions from control and *Pf*ATM1-V_m_ treated parasites was quantified using ferrozine as described earlier [31, 107]. The same total amount of protein (~1mg) from both treatments was assayed for quantification of iron and glutathione. Total glutathione was assayed using the Glutathione Assay kit (Cayman Chemical Company, USA).

### Generation of transgenic *T*. *gondii*

Tachyzoites were transfected by electroporation [108]. The *Tg*ATM1-Ty-U1 strain was generated by co-transfection of 20 μg of a CRISPR/Cas9 expression plasmid [109] targeting the 3′-UTR of the gene of interest (TGGT1_269000), and a PCR-generated homology repair template encoding the 3-Ty-U1 tagging construct as well as a hypoxanthine-xanthine-guanine phosphoribosyl transferase (HXGPRT) resistance cassette [58, 110]. The corresponding primers are listed in **S2 Table**. Transfected parasites were selected for 1 week with 25 μg/ml mycophenolic acid and 50 μg/ml xanthine for HXGPRT positive selection, and stable transfectants were cloned by serial dilution [111].

Codon remodelled copies of *Tg*ATM1-myc, *Sc*ATM1-myc, and *Pf*ATM1-myc were ordered from GenScript and inserted into a pUC57 vector. The copies were excised from the pUC57 vector using EcoRI and EcoRV sites and inserted in a linearized (EcoRI and EcoRV) pUPRT-pTUB-PEPCK-4Myc vector. Integration was checked by PCR. For transfection, 60 μg of pUPRT-pTUB-*XX*ATM1-4myc vector were linearized by NdeI and mixed with 20 μg of gRNA targeting the UPRT locus. Transfected parasites were selected for one week with 5-fluorodeoxyuridine (FUDR) for positive selection, and stable transfectants were cloned by serial dilution.

NBP35 (TGGT1_280730) was tagged at its 3′ by end through insertion of a 3-HA tag and a dihydrofolate reductase (DHFR) resistance cassette [112] using the CRISPR/Cas9 strategy as above. Transfected parasites were selected for 1 week with 1 μM pyrimethamine, and stable transfectants were cloned by serial dilution.

### Genomic DNA extraction and integration PCR

Genomic DNA was extracted from *T*. *gondii* parasites using the Wizard Genomic DNA Purification Kit (Promega, USA). Integration of all constructs was confirmed by PCR (primers in **S2 Table**) using GoTaq DNA Polymerase (Promega, USA) with the integration primers listed in **S2 Table**.

### Plaque assays and intracellular growth assays

Serial dilutions of freshly egressed *T*. *gondii* culture were incubated on confluent monolayers of HFF cells and left to grow for 7 days in the absence or presence of Rapa (added to a final concentration of 50 nM). After 7 days, host cell monolayers were fixed with 4% paraformaldehyde (PFA) for 10 min, washed with phosphate-buffered saline (PBS), and plaques were revealed by staining with a crystal violet solution (12.5 g crystal violet, 125 ml ethanol mixed with 500 ml water containing 1% (w/v) ammonium oxalate). Plaque sizes were quantified using the polygonal selection icon after setting the scale and entering the distance in ImageJ.

For growth assays, confluent HFFs grown on coverslips were inoculated with 10 μl of a freshly lysing *T*. *gondii* culture. Parasites were pre-treated with Rapa and treated with Rapa during the growth assay as indicated. Twenty-four hours after inoculation, parasites were fixed with 4% PFA-glutaraldehyde for 10 min, prior to quenching with 0.1 M glycine/PBS. Infected host cells were permeabilized (0.2% Triton X-100/PBS), blocked (2% BSA/0.2% Triton X-100/PBS), and probed with a polyclonal rabbit antibody against the pellicle marker GAP45 [113] diluted in 2% BSA/0.2% Triton X-100/PBS for 1 hour (polyclonal rabbit anti-IMC (1:2000). Samples were washed (3 × 5 min, 0.2% Triton X-100/PBS) and probed with a secondary antibody prior to washing as for the primary antibody (anti-rabbit Alexa fluor 594, Invitrogen, USA). The coverslip was mounted on microscopy slides using DAPI Fluoromount-G (Invitrogen, USA). Slides were viewed on a Nikon Eclipse Ti inverted microscope (Nikon, Japan). The number of parasites was counted in 100 vacuoles per condition in triplicates and replicated in three independent biological replicates.

### Recombinant protein purification

Gene segments encoding the predicted mature form of *Pf*ATM1 (Plasmo DB ID: *PF*3D7_1352100, amino acids 43–1049) and its domains, *Pf*ATM1-NTE (aa 43–305) and *Pf*ATM1-CTD (aa 807–1049) were PCR-amplified using primers listed in **S2 Table.** The sequence encoding *Pf*NBP35 (*PF*3D7_0910800, aa 23–356) and *Pf*CFD1 (*PF*3D7_1128500, aa 96–568) were also PCR-amplified from genomic DNA (primers in **S2 Table).** The resulting PCR product of *Pf*ATM1 (3018 bp) was cloned in β-pET28a(+)-β expression vector (gift from Prof. Hiroshi Omote, Okayama University, Japan) [78] to express recombinant *Pf*ATM1 with C-terminal and N-terminal 6XHis-tags (**Fig 5D**). The clone was confirmed by SacI and SalI and DNA sequencing. *Pf*ATM1-NTE (786 bp), *Pf*ATM1-CTD (729 bp), *Pf*NBP35 (993 bp) and *Pf*CFD1 (1479 bp) were cloned into pET23a expression vector, and confirmed by restriction digestion using SacI and SalI followed by DNA sequencing. DNA segments encoding *Pf*IscU and *Pf*IscA2 [31] were sub-cloned in pGEX-KG vector and confirmed by BamHI and SalI digestion.

For expression of 6X-His tagged β-*Pf*ATM1-β (henceforth referred to as recombinant *Pf*ATM1), *E*. *coli* C43 strain was co-transformed with pET28a-β-*Pf*ATM1-β and the RIG plasmid, and grown in LB media containing 50 μg/mL kanamycin sulfate until OD_600_ reached 0.6–0.8 followed by induction with 1 mM IPTG at 20°C for 16 h. For purification, we followed the procedure of [79]. Briefly, after sonication and removal of cell debris and insoluble fraction by centrifugation at 5860 × g, the supernatant was ultracentrifuged (SW41 Ti rotor, Beckman–Coulter) at 150,000 × g for 1 h. The membrane protein containing pellet was suspended and adjusted to 2 mg/ml with buffer (20 mM Tris-Cl pH 7.5, 100 mM NaCl, 10 mM KCl, 5% glycerol), followed by addition of combination of integral membrane protein detergents DDM (0.5%) and Triton X-114 (1%) (Cayman Chemical Company, USA). Membrane proteins were solubilized by incubation on magnetic stirrer for 30 min at 4°C followed by ultracentrifugation at 150,000 × g at 4°C for 1 h. The supernatant containing extracted membrane proteins was diluted two-fold and then purified using Ni-nitrilotriacetic acid (Ni-NTA) Superflow (Qiagen, Germany) pre-equilibrated with the buffer consisting of 20 mM Tris·Cl pH 8.0, 100 mM NaCl, 10 mM KCl and 5% glycerol. After protein binding, the column was washed with buffer containing 10 mM imidazole and 1% octyl glucoside (Cayman Chemical Company, USA) and eluted with buffer containing 300 mM imidazole and 1% octyl glucoside. The purity of eluted fractions was checked on 8% SDS-PAGE. For further purification of *Pf*ATM1, size exclusion chromatography (SEC) on Superdex S-200 column (GE Healthcare/Cytiva, USA) was carried out in an AKTA purifier system (GE Healthcare/Cytiva, USA). High molecular weight protein markers (GE Healthcare, USA) were used to plot the standard curve for estimation of molecular weight of *Pf*ATM1. To determine the oligomeric status of *Pf*ATM1, fractions collected during size exclusion chromatography were electrophoresed on 6% native-PAGE.

For expression, *E*. *coli* BL21 (DE3) expression host was co-transformed with RIG plasmid and pET23a-*Pf*ATM1-CTD. *Pf*ATM1-NTE, *Pf*NBP35 and *Pf*CFD1 were expressed in *E*. *coli* BL21 Codon Plus. The transformed cells were grown in LB media supplemented with ampicillin (100 μg/ml) and chloramphenicol (25 μg/ml) at 37°C until OD_600_ reached 0.5, followed by induction with 0.5 mM IPTG for 16 hours at 16°C. pGEX-KG-*Pf*IscU was expressed in *E*. *coli* BL21 (DE3) and pGEX-KG-*Pf*IscA2 was expressed in *E*. *coli* C43, co-transformed with the RIG plasmid, with induction at 20°C.

Recombinant 6XHis-tagged proteins were purified by affinity chromatography using Ni-NTA Superflow and eluted in 20 mM Tris-Cl pH 7.5, 100 mM NaCl, 10 mM KCl, 5% glycerol and 300 mM imidazole. The GST-tagged proteins (*Pf*IscU and *Pf*IscA2) were purified using glutathione-agarose resin (Macherey-Nagel, Germany) and eluted in 20 mM Tris-Cl pH 7.5, 100 mM NaCl, 10 mM KCl, 5% glycerol and 20 mM reduced glutathione. *Pf*ATM1-CTD, *Pf*NBP35 and *Pf*CFD1 were further purified by SEC through Superdex S-75 column (GE Healthcare, USA).

Purified proteins were checked on 10% SDS-PAGE and confirmed by western blotting using anti-6XHis antibody (Ab) (Santa Cruz Biotechnology, USA) for *Pf*ATM1, *Pf*ATM1-NTE, *Pf*ATM1-CTD, *Pf*NBP35 and *Pf*CFD1 and anti-GST antibody (Sigma-Aldrich, USA) for GST-tagged proteins *Pf*IscU and *Pf*IscA2. To cleave the GST-tag, proteins were treated overnight with 100 U of thrombin at 4°C on the affinity column and eluted. The concentration of all purified proteins was determined by Pierce^TM^ BCA protein assay kit (Thermo Fisher Scientific, USA).

*Pf*ATM1 mutants (E972Q and R534E) were generated using Q5 site-directed mutagenesis kit (New England Biolabs, USA) (primers in **S2 Table**) and confirmed by DNA sequencing.

### CD spectroscopy

CD spectra of *Pf*ATM1 (5 μM) in 20 mM Tris-Cl pH8 and 100 mM NaCl was scanned at 25°C with a spectral scan range of 190 to 250 nm in a CD spectropolarimeter (J-1500, JASCO, Japan). Total of three accumulations were taken during the scan.

### TEM

*Pf*ATM1 and reconstituted proteoliposomes were visualized using the negative stain TEM technique. Purified *Pf*ATM1 (1mg/ml protein in 20 mM Tris-Cl pH 7.5, 100 mM NaCl) or proteoliposomes (in the same buffer) were allowed to adsorb to freshly glow-discharged carbon coated 400 mesh copper grids and blotted off the edge of the grids using a Whatman filter paper. 2% (w/v) aqueous uranyl acetate was used to perform negative staining followed by air drying. The grids were analysed under a TEM (Jeol JEM1400; Jeol, Japan) at 100 kV after performing astigmatism correction and magnification calibration. Images were collected using an Orius SC200B CCD camera (Gatan, UK) and analysed using Digital Micrograph software (Gatan, UK).

### Polyclonal antisera and western blotting

Purified recombinant *Pf*ATM1-NTE and *Pf*NBP35 were used as antigens to generate antisera in rabbit (New Zealand White) as described earlier [114]. Antibodies from rabbit polyclonal immune serum were affinity purified using *Pf*ATM1-NTE and *Pf*NBP35 proteins immobilized on nitrocellulose membrane.

For western blotting of the parasite lysate, parasites at the mid- to late-trophozoite stage were released from erythrocytes by 0.05% saponin lysis. After washing with cold PBS, the parasite pellet was resuspended in RIPA buffer (50 mM Tris-Cl, 150 mM NaCl, 1.0% (v/v) NP-40, 0.5% (w/v) sodium deoxycholate, 0.1% (w/v) SDS at pH 7.4) with 1X protease inhibitor cocktail (Sigma-Aldrich, USA), incubated for 1 hour at 4°C on a tube rotator, sonicated for 2 min, and centrifuged at 13,800 × g for 15 min. The lysate (supernatant) was suspended in 1X Laemmli buffer and electrophoresed on 8 or 10% SDS-PAGE. For western blotting, purified anti-*Pf*ATM1 and anti-*Pf*NBP35 Abs were used as primary Abs, and HRP-tagged goat anti-rabbit Ab (1:10,000, Santa Cruz Biotechnology, USA) as secondary Ab. Immobilon Western Chemiluminescent HRP Substrate was used to probe blots using a chemiluminescence detection system (Amersham Image Quant 800, Cytiva, USA).

For immunoblot analysis in *T*. *gondii*, pellets from extracellular tachyzoites were resuspended in Laemeli buffer under a reducing condition (1 mM of DTT) and then boiled (at 98°C) for 5 min before loading onto a gradient SDS–PAGE gel for separation. Proteins were transferred to a hybond ECL nitrocellulose membrane using a wet transfer system (Bio-Rad Laboratories, Hercules, USA). Antibodies were diluted in PBS/0.05% Tween20/ 5% skimmed milk. Primary antibodies were monoclonal hybridoma mouse α-Ty (1:10, BB2, Invitrogen, USA) and monoclonal hybridoma mouse anti-myc (1:10, 9E10, Invitrogen, USA). Polyclonal rabbit α-catalase was used as a loading control (1:5,000) [115]. Secondary antibodies (goat anti-mouse, horse radish peroxidase (HRP)-conjugated, Sigma Aldrich, USA; and goat anti-rabbit, HRP conjugated, Sigma Aldrich, USA) were visualized using the SuperSignal West Pico PLUS Chemiluminescent Substrate (Thermo Fisher Scientific, USA). Images were acquired using the Bio-Rad ChemiDoc MP Imaging System and images were processed using Bio-Rad Image Lab software.

### [Fe-S] reconstitution and transfer assay

UV visible spectroscopy (spectral scan from 270–700 nm) was performed in Epoch (BioTek, USA). Conversion of holo- to apo-form of *Pf*ATM1 was carried out as described earlier [29]. Chemical Reconstitution of [4Fe-4S] on thrombin-cleaved *Pf*IscU and *Pf*IscA2 was carried out as previously described [29, 116]. In-vitro [Fe-S] transfer assays were performed in an anaerobic chamber (PLAS-Labs, USA). For direct transfer of [4Fe-4S] from holo-*Pf*IscU/ holo-*Pf*IscA2 on apo-*Pf*ATM1, apo-*Pf*ATM1 was incubated with four molar excess of holo-*Pf*IscU or holo-*Pf*IscA2 with or without 2 mM DTT in buffer containing 25 mM Tris–Cl pH 7.5 and 100 mM NaCl for 6 hours at 4°C [31]. The reaction mixture was loaded on Ni-NTA beads, bound *Pf*ATM1 was eluted using imidazole, and analysed by UV-VIS scan.

### ATPase assay and kinetics

ATPase activity of *Pf*ATM1 and its mutants was measured using EnzCheck Phosphate Assay Kit (Invitrogen, Thermo Fisher Scientific, USA). In reactions with GSH/GSSG, 3 mM of each glutathione derivative was used with 500 nM of holo/apo *Pf*ATM1 in 200 μl reaction mixture (containing 40 μl MESG substrate, 2 μl PNP enzyme, 10 μl 20 X reaction buffer and 20 μl 10X ATPase buffer). All background control values were subtracted from the readings. Kinetics of ATP hydrolysis in the presence of GSSG was determined with increasing concentrations of ATP and 500 nM *Pf*ATM1. K_m_, K_cat_ and catalytic efficiencies (K_cat_/K_m)_) were calculated using GraphPad Prism 5.

### Spectrofluorometric analysis

Variation in intrinsic tryptophan fluorescence was monitored in a solution containing 10 μM *Pf*ATM1, 20 mM Tris-Cl pH 8, 100 mM NaCl and 10 mM KCl with increasing concentrations of GSSG (1–10 mM). After excitation at 280 nm, emission spectra were recorded from 300–400 nm in Cary Eclipse Fluorescence Spectrophotometer (Agilent Technologies, USA). Buffer alone with the corresponding GSSG concentration was used as control.

### Immunofluorescence assays

*P*. *falciparum*-infected erythrocytes at different asexual stages were fixed in glutaraldehyde and paraformaldehyde, followed by permeabilization with 0.2% Triton X-100, and processed as described earlier [106, 117]. After blocking, cells were incubated overnight at 4°C in purified anti-*Pf*ATM1 Ab (1:100) and mouse anti *Pf*HU (1:200) as apicoplast marker [60]. For co-labelling with mitochondrial marker dye, cells were incubated with 50 nM Mitotracker Red CMXRos (Invitrogen, Thermo Fisher scientific, USA) prior to fixation. AlexaFluor 488-tagged anti-mouse Ab (1:1000, Invitrogen) and AlexaFluor 568-tagged anti-rabbit Ab (1:1000, Invitrogen) were used to detect *Pf*HU and *Pf*ATM1, respectively. AlexaFluor 514- tagged anti-rabbit Ab (1:1000, Invitrogen) was used as secondary antibody to detect *Pf*ATM1 in Mitotracker Red labelled cells. Nuclei were stained with DAPI (0.2μg/mL, Sigma-Aldrich). Cells were layered on poly-L-Lysine coated coverslips, washed and mounted in anti-fade mounting media (Invitrogen), and scanned on Leica SP8 confocal microscope under 63X oil immersion objective. Co-localization was quantified using LasX software.

Mitochondria and nuclei of Vivo-morpholino (*Pf*ATM1-V_m_)-treated *P*. *falciparum* 3D7 blood cultured parasites were labelled with 50 nM Mitotracker Red CMXRos (Invitrogen) and DAPI (0.2 μg/ml) for 30 min at 37°C. After three washes with 1X PBS, cells were diluted and spotted on poly-L-Lysine coated coverslips. After a 2 hours incubation and two more washes with 1XPBS, the cover-slips were mounted and scanned as above.

For *T*. *gondii*, HFF monolayers grown on coverslips were inoculated with tachyzoites and grown at 37°C. Cells were subsequently fixed with 4% PFA-Glu for 10 min, then quenched with 0.1 M glycine/PBS. Cells were permeabilized (0.2% Triton X-100/PBS), blocked (2% BSA/0.2%Triton/PBS), and probed with primary antibodies (1 h) prior to washing (0.2%Triton/PBS) and incubation with secondary antibodies (Alexa488 or Alexa594 conjugated goat anti-mouse/rabbit, respectively). Coverslips were mounted in DAPI FluoromountG (Invitrogen, USA). Confocal images were generated with a LSM700 laser scanning confocal microscope (Zeiss, Germany). Image stacks were processed with ImageJ. Antibodies used in the study (used dilution): polyclonal rabbit anti-GAP45 (1:5000) [113], monoclonal hybridoma mouse α-Ty (1:10, BB2, Invitrogen, USA), monoclonal hybridoma mouse anti-myc (1:10, 9E10, Invitrogen, USA), polyclonal rabbit anti-chaperone 60 (CPN60, 1:2000) [118], polyclonal rabbit anti- mitochondrial heat shock protein 70 (mHsp70, 1:2,000) [59]. Secondary anti-mouse Alexa fluor 488 (Invitrogen, USA), anti-rabbit Alexa fluor 594 (Invitrogen, USA).

### Protein pull-down assay

6XHis-tagged *Pf*ATM1-CTD or *Pf*ATM1-NTE bound to Ni-NTA beads were used as bait to pull down interacting partners from the parasite lysate as described previously [114]. After elution, proteins were separated on 14% SDS–PAGE followed by western blotting using anti-*Pf*IscU, anti-*Pf*IscA2 [31] and anti-6XHis antibodies in the experiment with *Pf*ATM1-CTD as bait. Anti-*Pf*HU was used as control to confirm that *Pf*ATM1-CTD bound specifically to ISC pathway components. After pull down from parasite lysate using *Pf*ATM1-NTE as bait, samples were loaded on 10% SDS-PAGE. Western blotting was carried out using anti-*Pf*NBP35 and anti-6XHis Abs. Beads alone (without bait protein) and beads tagged with 6XHis-*Ec*EngA (kind gift from Dr. Balaji Prakash) served as negative control baits.

### In vitro complexation assay

Purified *Pf*ATM1, *Pf*NBP35 and *Pf*CFD1 were used for complex formation in vitro. Equimolar ratio of *Pf*NBP35 and *Pf*CFD1 was incubated for 1 hour or 16 hours at 4°C to detect formation of *Pf*NBP35-*Pf*CFD1 complex. Further, 40 nM each of *Pf*NBP35 with *Pf*CFD1, *Pf*ATM1 with either *Pf*NBP35 or *Pf*CFD1, and *Pf*ATM1 with *Pf*NBP35+*Pf*CFD1 complex were incubated for 16 hours at 4°C. Native-PAGE loading dye (2.5X TBE, 50% glycerol and 0.1% bromophenol blue) was added to the samples at 1X final concentration. For investigating *Pf*NBP35-*Pf*CFD1 complex formation, samples were electrophoresed on 6–10% gradient native-PAGE. *Pf*ATM1 complexation with *Pf*NBP35 and *Pf*CFD1 was detected on 6–8% native-PAGE. Gels were stained with Coomassie Brilliant Blue R-250.

### *Pf*ATM1-liposomes and transport assay

Purified *Pf*ATM1 or *Pf*ATM1-E972Q were reconstituted into proteoliposomes using the detergent-mediated method as described by [119]. Briefly, 3:1 molar ratio of cholesterol and phosphatidylethanolamine (PE) (Sigma-Aldrich) dissolved in chloroform [78] was divided into 70 μl aliquots 10 ml test tubes. Nitrogen gas was purged to remove all chloroform. The thin lipid films were placed under vacuum for 4–5 h. The lipid films were then hydrated in 350 μl transport buffer (17 mM Tris-Cl pH7.5, 85 mM NaCl) and bath-sonicated until homogeneity [120]. To prepare unilamellar liposomes, 250 μl of lipid sample was extruded in Avanti mini-extruder with 400 nm polycarbonate filter, pre-equilibrated with transport buffer. Liposomes were stored at 4°C. Purified *Pf*ATM1 and *Pf*ATM1-E972Q were dialysed in 1% n-octylglucoside (n-OG) containing transport buffer. Unilamellar liposomes were also saturated with 1% n-OG [120]. 230 μl of n-OG saturated unilamellar liposomes and 770 μl of dialysed protein were mixed together. The protein-lipid-detergent mixture was incubated at room temperature for 2 hours and then applied to an NAP5 desalting column (GE Healthcare, USA) pre-equilibrated with transport buffer. The cloudy void fraction which contains proteoliposomes was collected [121]. Detergent was further removed using 20 mg/ml slurry of polystyrene biobeads SM-2 (Bio-Rad Laboratories, USA) added to the proteoliposomes mixture and nutated overnight at 4°C. The supernatant was subjected to ultracentrifugation at 140,000 × g (SW41 Ti rotor, Beckman Coulter) for 45 min to pellet proteoliposomes. Proteoliposomes resuspended in transport buffer were analysed by dynamic light scattering (DLS) scan in Zetasizer Nano ZS (Malvern Panalytical, UK). Control liposomes lacking protein were prepared following the same procedure.

Transport activity was of proteoliposomes was assayed as described by Fan and colleagues [56]. In brief, 1 ml reaction mixture was prepared in transport buffer (85 mM NaCl, and 17 mM Tris-Cl pH 7.5) with 10 mM Mg-ATP [122], 2.5 mM GSSG and 5 mg/ml of proteoliposomes. All reactions were carried out at 37°C for a total duration of 90 min with sample taken out at 15 min intervals. 150 μl sample taken at each time point was added to 10 ml of cold transport buffer. All the samples were ultracentrifuged at 140,000 × g for 10 min at 4°C. Liposomes without protein were taken as a negative control. *Pf*ATM1 proteoliposomes without GSSG were used as background control. The pellet obtained after ultracentrifugation was rinsed five times with cold transport buffer, resuspended in 100 μl of solubilization buffer (transport buffer with 2% N-lauroyl sarcosine), and incubated for 2 hours at 4°C. Samples were centrifuged at 14,800 × g to remove bubbles. 50 μl of the supernatant of detergent-lysed liposomes was used for GSSG quantification using the Glutathione Assay Kit (Cayman Chemical Company, USA). GSSG standard curve was used to estimate transported GSSG concentration in all samples. Reactions were carried out in triplicate. The transport rates were not corrected for the orientation of the protein in reconstituted liposomes.

### Statistical analysis

Graphs were generated using Origin 5.0 and GraphPad-Prism 10. P values were calculated using two tailed, unpaired t-test.

### Cell harvest for mass spectrometry analyses

Following the indicated treatment, freshly egressing parasites (independent triplicates per condition) were harvested. To this end, culture medium was gently removed without disturbing the host cell monolayer, the cell layer was scraped, collected, and passed 3x through 26G needles before removing host-debris through filtration (3 μm pore size, Millipore, USA). Filters were rinsed with additional ice-cold PBS to maximize the yield of parasites. The parasite solution was centrifuged at 2,500 × g for 25 min at 4°C. The supernatant was removed, and parasite numbers adjusted to 1.5x10^8^ parasites per sample. Two additional wash steps (2,500 × g for 10 min) were carried out swiftly at 4°C with cold PBS for GC-MS analyses or with PBS containing protease inhibitor (Pierce Protease Inhibitor, EDTA free, Thermo Scientific, USA) for proteomic analyses. Parasite pellets were snap frozen and stored at −80°C until metabolite extraction.

### Metabolite profiling by GC-MS

#### Sample preparation

Samples for GC-MS analysis were prepared as previously described [123]. In brief, pellets were gently thawed on ice and metabolites extracted through addition of 50 μl CHCl_3_ and vortexing, followed by addition of 200 μl of CH_3_OH/H_2_O (3:1), containing 5 μM scyllo-inositol (Sigma-Aldrich, USA) as internal standard. The insoluble fraction was pelleted by centrifugation at 20,000 × g for 5 min at 4°C and the supernatant transferred to a new microtube containing 100 μl of H_2_O. The polar and apolar phases were separated by centrifugation at 20,000 × g for 10 min at 4°C. The polar phase was collected and dried sequentially (50 μl at a time) in a glass vial insert suitable for mass spectrometry vials using a centrifugal evaporator. Samples were methoximated by resuspending the dried extract in 20 μl of pyridine (Sigma-Aldrich, USA) containing methoxyamine-hydrochloride (20 mg/ml; room temperature, overnight, Sigma-Aldrich). The next day, samples were silylated through addition of 20 μl N,O-bis(trimethylsilyl)trifluoroacetamide with 1% trimethylchlorosilane (BSTFA-TMCS, Cerilliant, Sigma-Aldrich).

#### GC-MS Analysis

Samples were analysed using an 8890 GC System connected to a 5977B GC/MSD (Agilent Technologies, USA) equipped with a VF-5ms column (length: 30 m, +10 m EZ-guard, diameter: 0.25 mm, film: 0.25 μm, Agilent Technologies, USA). The inlet was set to 270°C, the MS transfer line to 280°C, the quadrupole to 150°C and the ion source to 230°C. 1 μl of sample was injected and analysed in splitless mode via electron ionization. The oven gradient was as follows: 70°C (1 min); 70°C to 295°C at 12.5°C/min; 295°C to 320°C at 25°C/min; 320°C for 2 min. Samples were analysed in scan mode (*m/z* 70–700) with 5.5 min solvent delay.

#### Data analysis and statistics

All metabolites were identified based on the retention time and mass spectra of authentic standards or through high confidence (>80%) identification by the NIST mass spectral library. The relative abundance of each metabolite was determined by measuring the signal (area under the curve) and normalizing it to the signal of the internal standard (scyllo inositol) in the corresponding sample. The relative concentration was expressed in respect to the parental strain −Rapa. Signal intensities were determined using Mass Hunter Qualitative Analysis Software, 12.1 (Agilent Technologies, USA). Calculations were performed using Excel (Microsoft, USA). The above analysis was repeated for a total of three independent experiments. The means and standard deviation are provided for each experiment. Relative abundances were compared by performing a One-way ANOVA followed by Tukey multiple pair wise comparisons, using GraphPad Prism 10. p-values are given for relevant comparisons.

### Proteomics

#### Sample preparation

Cell pellets were resuspended in 50 μl of 0.1% RapiGest Surfactant (Waters, USA) in 50 mM ammonium bicarbonate (AB). Samples were heated for 5 min at 95°C. Lysis was performed by sonication (6 × 30 sec.) at 70% amplitude and 0.5 pulse. Samples were kept 30 sec on ice between each cycle of sonication. Samples were centrifuged for 10 min at 14,000 × g. Protein concentration was measured by Bradford assay and 50 μg of each sample was subjected to protein digestion as follows: sample volume was adjusted to 100 μl with 0.1% RapiGest in 50 mM AB. 2 μl of Dithioerythritol (DTE) 50 mM were added and the reduction was carried out at 37°C for 1 h. Alkylation was performed by adding 2 μl of iodoacetamide 400 mM during 1 hour at room temperature in the dark. Overnight digestion was performed at 37°C with 10 μl of freshly prepared trypsin (Promega; 0.1 μg/μl in 50 mM AB). To remove RapiGest, samples were acidified with trifluoroacetic acid (TFA), heated at 37°C for 45 min. and centrifuged 10 min. at 17,000 × g. Supernatants were then desalted with a C18 microspin column (Harvard Apparatus, USA) according to manufacturer’s instructions, completely dried under speed-vacuum and stored at -20°C.

#### ESI-LC-MSMS analysis

Samples were dissolved at 1 μg/μl with loading buffer (5% CH3CN, 0.1% formic acid, FA). Biognosys iRT peptides were added to each sample and 2 μg of peptides were injected on column. LC-ESI-MS/MS was performed on an Orbitrap Fusion Lumos Tribrid mass spectrometer (Thermo Fisher Scientific, USA) equipped with an Easy nLC1200 liquid chromatography system (Thermo Fisher Scientific, USA). Peptides were trapped on an Acclaim pepmap100, C18, 3 μm, 75 μm × 20 mm nano trap-column (Thermo Fisher Scientific, USA) and separated on a 75 μm × 500 mm, C18 ReproSil-Pur (Dr. Maisch GmBH), 1.9 μm, 100 Å, home-made column. The analytical separation was run for 135 min using a gradient of H_2_O/FA 99.9%/0.1% (solvent A) and CH3CN/H2O/FA 80.0%/19.9%/0.1% (solvent B). The gradient was run from 8% B to 28% B in 110 min, then to 42% B in 25 min, then to 95%B in 5 min with a final stay of 20 min at 95% B. Flow rate was of 250 nl/min and total run time was of 160 min. Data-Independent Acquisition (DIA) was performed with MS1 full scan at a resolution of 60,000 (FWHM) followed by 30 DIA MS^2^ scan with fix windows. MS1 was performed in the Orbitrap with an AGC target of 1 × 10^6^, a maximum injection time of 50 msec and a scan range from 400 to 1240 m/z. DIA MS2 was performed in the Orbitrap using higher-energy collisional dissociation (HCD) at 30%. Isolation windows was set to *m/z* 28 with an AGC target of 1 ^x^ 10^6^ and a maximum injection time of 54 msec.

#### Data analysis and statistics

DIA raw files were loaded into Spectronaut v.15 (Biognosys) and analysed by direct DIA using default settings. Briefly, data were searched in *T*. *gondii GT1* database (ToxoDB, release 57, 8460 entries). Trypsin was selected as the enzyme, with one potential missed cleavage. Variable amino acid modifications were oxidized methionine. Fixed amino acid modification was carbamidomethyl cysteine. Both peptide precursor and protein FDR were controlled at 1% (*Q value* < 0.01). Single Hit Proteins were excluded. For quantitation, Top 3 precursor area per peptides were used, “only protein group specific” was selected as proteotypicity filter and normalization was set to “automatic”. The quantitative analysis was performed with MapDIA tool, using the precursor quantities extracted from Spectronaut output. No further normalization was applied. The following parameters were used: min peptides = 2, max peptides = 10, min correl = -1, Min_DE = 0.01, max_DE = 0.99, and experimental design = replicate design. Proteins were considered to have significantly changed in abundance with an FDR ≤ 0.05 and an absolute fold change FC≥ |1.5| (log2FC ≥ |0.58|).

### Extracellular flux analysis (Seahorse XFe96)

The extracellular flux analysis was carried out as previously outlined [64] and described, in brief, here. Freshly egressed parasites were harvested and washed in Seahorse XF Base Medium (Agilent Technologies, USA). The Base Medium used throughout the assay was supplemented with 10 mM glucose (Agilent Technologies, USA), 2 mM glutamine (Agilent Technologies, USA), and 1 mM pyruvate (Agilent Technologies, USA) and adjusted to pH 7.4. Parasites were counted and numbers adjusted to 3x10^7^ parasites/ml. Of this cell solution, 30 μl (9x10^5^) were seeded into wells of a XFe96 culture plate (Agilent Technologies, USA), following its coating with Cell-Tak cell adhesive (Corning, USA), according to the manufacturer’s guidelines. Additional 150 μl of XF Base Medium were added to each well and plates briefly incubated in a 37°C incubator without additional CO_2_. Injection ports of XFe96 sensor cartridges were loaded with Oligomycin (port A), carbonyl cyanide-p-trifluoromethoxyphenylhydrazone (FCCP) (port B), and rotenone/antimycin A (port C) resuspended in XF Base medium to obtain final concentrations of 2.5 μM, 2 μM, and 0.5 μM, respectively. The assays were run as follows: 3 basal measurement cycles, followed by 3 measurement cycles after injection of each compound, with each cycle consisting of 3 min mixing and 3 min measurement of the oxygen consumption rate (pmol O_2_/min) and media acidification rate (mpH/min). Each strain/condition was analysed in 8–14 technical replicates in 3 independent experiments. The data was analysed using Wave Software (Agilent Technologies, USA) and Excel (Microsoft, USA). The basal oxygen consumption rate and extracellular acidification rate were determined based on the three initial measurements, while the maximal OCR represents the values from the three measurements after injection of FCCP and the spare capacity is the difference between the basal and maximal respiration rate. Means and standard deviation are provided for each experiment. Values between conditions were compared by performing a One-way ANOVA followed by Tukey multiple pair wise comparisons, using Graph Pad Prism 10. p-values are given for relevant comparisons.

### Co-immunoprecipitation

For co-immunoprecipitation, freshly egressed *T*. *gondii* parasites were pelleted and lysed in 950 μl of 10 mM HEPES, pH 7.4, 2 mM EDTA, 150 mM NaCl and 10% glycerol with 0.1% of Triton X-100. 50 μl of the lysate was used. After separation of the soluble and insoluble fraction, the pellet was resuspended in 300 μl of 1x of Laemmli buffer. The soluble fraction was separated into two microtubes and incubated on a shaker for 2 hours at 40°C with either anti-Ty (mouse) or anti-myc (mouse) antibodies, kindly gifted by Prof. Ermmano Candolfi, University of Strasbourg, France. Protein A Sepharose CL-4B antibody purification beads (Cytiva, USA) were added following an incubation of 2 hours at 40°C on a shaker. The solution was washed three times with the above buffer. 50 μl were sampled after the first wash for the flow-through. After the last wash, beads were resuspended in 1x Laemmli buffer and loaded into a gradient SDS-PAGE gel. Membranes were incubated with the primary antibodies anti-Ty (rabbit, Sigma-Aldrich) or anti-myc (rabbit), kindly gifted by the Prof. Chris Tonkin, WEHI, Melbourne, Australia.

## Supporting information

S1 Fig Phylogeny and sequence alignment. (**A**) Phylogenetic analysis of *Pf*ABCB2 (*Pf*MDR2) and *Pf*ABCB6 (*Pf*MDR6) (sequences in **S1 Table**). LG+R+F model was found to be the best substitution model predetermined by ATGC: SMS (Smart model selection) in PhyML 3. 0, visualised by iTOL software. Bootstrap support values (in red) and branch lengths of the unrooted tree are shown. Green arrow indicates a more recent common ancestor shared between *Pf*ABCB2 (*Pf*MDR2) and eukaryotic HMTs. (**B**) Multiple sequence alignment of ATM1 homologs from apicomplexan parasites. ClustalW alignment of ATM1- like proteins of *P*. *falciparum*, *Toxoplasma gondii* and *Cryptosporidium parvum* with *Saccharomyces cerevisiae* ATM1, bacterial ATM1 of *Novosphingobium aromaticivorans*, fungal ATM1 of *Chaetomium thermophilum*, ATM3 (ABCB25) of *Arabidopsis thaliana*, and human ABCB7. The NTE, transmembrane region, insertion and CTD with conserved ATP-binding sites are indicated. Positions showing the extent of full-length recombinant *Pf*ATM1 (FL) and its CTD and NTE domains are marked. Black arrows and blue circles indicate GSSG binding sites in *Na*ATM1 and GSH binding sites in *Sc*ATM1, respectively. [2Fe-2S]GS_4_ cluster coordination sites in *Sc*ATM1 and *Hs*ABCB7 are marked by grey and orange circles, respectively. Gatekeeper residues in *Na*ATM1, *Ct*ATM1 and *Pf*ATM1 are boxed in blue. The TM6 helix of *Na*ATM1 is indicated by a blue dashed line. The predicted α-helix (PSIPRED prediction) in the NTE of *Pf*ATM1 is shown by a red line and the hydrophobic stretch in this region is indicated by a green dashed line. Poly(Asn) in the NTE is indicated by a blue line.
(PDF)

S2 Fig Knockdown strategy for *Tg*ATM1 and *Pf*ATM1. (**A**) Cartoon scheme showing the principle of the conditional downregulation strategy employed for *Tg*ATM1 based on dimerizable Cre recombinase (DiCre) and mRNA destabilization. The U1-recognition site moves closer to the stop codon upon activation of the DiCre and causes destabilization and degradation of the mRNA resulting in reduced expression of *Tg*ATM1. (**B**) Genomic integration PCR probing the parental line (DiCreU1) and *Tg*ATM1-Ty-U1 parasites to validate the integration of the intended construct, including a Ty-tag, a U1 recognition site, loxP sites and a selection cassette, in the 3′UTR of *Tg*ATM1. The approximate primer binding sites are given in the scheme and primers are listed in S2 Table.
(PDF)

S3 Fig Downregulation of *Pf*ATM1. (**A**) Coomassie-stained SDS-PAGE of purified ~33 kDa *PfA*TM1-NTE. (**B**) Strategy for blocking translation of *Pf*ATM1 using *Pf*ATM1-Vm. (**C**) Time-dependent expression of *Pf*ATM1 in untreated (UT), control morpholino (C-V_m_) or *Pf*ATM1-V_m_ treated parasites at 72 hours (i), 96 hours (ii) and 144 hours (iii) post-treatment. Actin served as loading control. (**D**) Quantitative analysis of western blots after V_m_ treatment. *Pf*ATM1 levels at 72, 96, and 144 hours post-treatment in untreated and *Pf*ATM1-V_m_ treated cells were normalized with actin and plotted relative to levels in untreated cells (left). Two biological replicates were analysed by two-tailed Student’s t-test; mean and SD are plotted. *Pf*ATM1 levels from untreated, C-V_m_ treated and *Pf*ATM1-V_m_ treated cells were compared at 96 hours post-treatment (right). Mean and SD of three biological replicates are plotted. Significant differences (p <0.05) are in bold. (**E**) Giemsa-stained parasites representing predominant stages from untreated, C-V_m_ and *Pf*ATM1-V_m_ treated sets at different times post-treatment. (**F**) Parasite stages as % of total parasites in the second and third infection cycle post-treatment. P values (t-test) are shown for comparison of ring stage between control V_m_ and *Pf*ATM1-V_m_ at 96 hours, and for late trophozoites between control V_m_ and *Pf*ATM1-V_m_ at 108 hours. Significant differences (p <0.05) are highlighted in bold. Parasitemia (%) is given at the top of the bars. (**G**) Confocal microscopy after staining *Pf*ATM1-V_m_ treated and untreated cells at 96 and 108 hours post-treatment. Cells were stained with DAPI and Mitotracker Red to detect any changes in mitochondrial morphology. Matching parasite stages were selected for comparison. (**H**) and (**I**) Western blot of organellar fraction from control untreated (H) and *Pf*ATM1-V_m_-treated cells (I) probed with antibodies against mitochondrial (*Pf*IscA2), apicoplast (*Pf*HU), nuclear (Histone H3) and cytosolic (α-tubulin) proteins. The cytosolic marker protein α-tubulin was not seen in the organelle pellet. Soluble fraction (SF), Pellet (P). (**J**) and (**K**) Total iron (J) and glutathione (K) levels in crude organelle fraction of untreated and *Pf*ATM1-V_m_ treated parasites at 108 hours post-treatment. The means and standard deviations from two independent biological replicates are shown. P values (t-test) are indicated.
(PDF)

S4 Fig ATM1 second copy complementation and mitochondrial function. (**A**) Scheme visualizing the strategy to insert second copies of myc-tagged ATM1s from *Toxoplasma*, *Plasmodium* and yeast (*Tg*, *Pf* and *Sc*, respectively) into the uracil phosphoribsyltransferase (UPRT) locus of *T*. *gondii*. (**B**) Modification of the UPRT locus was validated by genomic PCR. The approximate binding sites of the employed primers are shown in scheme A and sequences listed in S2 Table. (**C**) Immunofluorescence assay of *Tg*ATM1-Ty-U1 parasites and *Tg*ATM1-Ty-U1 parasites expressing a second copy of myc-tagged *Toxoplasma*- (*Tg*ATM1-Ty-U1/c*Tg*ATM1-myc), *Plasmodium*- (*Tg*ATM1-Ty-U1/c*Pf*ATM1-myc) or yeast ATM1 (*Tg*ATM1-Ty-U1/c*Sc*ATM1-myc). Parasites were stained with anti-Ty and anti-myc antibodies and merged signals are shown. Images are representative of three independent biological replicates. (**D** and **E**) Representative traces from an extracellular flux analysis following 0 or 72 hours of Rapa treatment of parental (DiCreU1), uncomplemented *Tg*ATM1-Ty-U1 parasites or *Tg*ATM1-Ty-U1 parasites expressing a second copy of myc-tagged yeast ATM1 (*Tg*ATM1-Ty-U1/c*Sc*ATM1-myc). The oxygen consumption rate (OCR, D) and extracellular media acidification rate (ECAR, E) are shown. The legend shown in D applies to both panels. Other overexpressing strains behaved similarly–see S3 Table and Fig 4E and 4F.
(PDF)

S5 Fig *Tg*ATM1 and *Pf*ATM1 dimerization. (**A**-**C**) Controls for Co-immunoprecipitation assay shown in Fig 5A-5C. Western blots as described in Fig 5A-5C but membranes were probed with anti-myc antibody. *Tg*ATM1-myc (A), *Pf*ATM1-myc (B) and *Sc*ATM1-myc (C) are indicated. Blots are representative of three independent biological replicates. (**D**) Recombinant *Pf*ATM1-CTD dimer breaks down into monomers upon treatment with DTT and urea. (**E**) Size exclusion chromatography on S200 column for purification of *Pf*ATM1 separates dimeric (P1) and monomeric protein with some degradation (P2). Absorbance at 280 nm is plotted against elution volume. Coomassie-stained SDS-PA gel for P1 and P2 is shown in Fig 5F. Inset, molecular weight standard plot for S200. (**F**) CD spectra of purified *Pf*ATM1 homodimer.
(PDF)

S6 Fig (**A**) Molecular structure model of *Pf*ATM1 (cyan) shown in Fig 5I superimposed on the *Na*ATM1 template (gray). The predicted TM6 in *Pf*ATM1 (residues 636–662, gold) overlays on the *Na*ATM1 TM6 helix (residues 312–338, salmon). (**B**) Molecular structure model of *Tg*ATM1 (cyan) shown in Fig 5J superimposed on the *Na*ATM1 template (gray). (**C**) MD simulation of *Pf*ATM1 docked with GSSG. RMSD plot after molecular dynamics simulation of *Pf*ATM1 dimer docked with GSSG and ATP. (**D**) 2-D interaction plot of two GSSG molecules with the two *Pf*ATM1 chains. (**E**) Spectrofluorometry of buffer alone and increasing concentrations of GSSG in buffer (as control for Fig 5L) (top). a.u. denotes arbitrary units for fluorescence intensity. The absorbance spectral scan of GSSG (in buffer) in the 300–400 nm range (bottom) to rule out an inner filter effect in Fig 5L. (**F**) Electrostatic potential map of the *Pf*ATM1 dimer model generated by PyMOL. The positively charged central cavity is encircled. (**G**) MD simulation (200 nsec) showing RMS fluctuation in gatekeeper residues of *Ct*ATM1 (PDB:7PRO) and the *Pf*ATM1 model.
(PDF)

S7 Fig Interaction of *Pf*ATM1 with ISC and CIA proteins. (**A**) *Pf*IscU and *Pf*IscA2 after thrombin cleavage for removal of GST from the purified fusion proteins. (**B-C**) Size exclusion chromatography of partially purified *Pf*NBP35 (B) and *Pf*CFD1 (C) through S75 column. Absorbance at 280 nm is plotted against elution volume. Inset, Molecular weight standard plot for S75. (**D**) Coomassie-stained SDS-PAGE of purified recombinant *Pf*NBP35 (~40 kDa) and (**E**) Purified recombinant *Pf*CFD1 (~57.5 kDa). (**F**) 6–10% native PAGE to detect complexation of *Pf*NBP35 and *Pf*CFD1. (**G**) 6–8% native PAGE to detect in vitro complexation of *Pf*ATM1 dimer (lane 1) with *Pf*CFD1 (lane 2) and *Pf*NBP35 (lane 3). The *Pf*NBP35-*Pf*CFD1 heterotetramer (lane 5) complexed with the *Pf*ATM1 dimer (lane 4). (**H)** 10–12% native PAGE to detect in vitro complexation of *Pf*ATM1-NTE and *Pf*ATM1-CTD with *Pf*NBP35. (**I**) Anti-*Pf*NBP35 serum detects a specific band of the expected size of ~51 kDa in the parasite lysate. (**J**) Pull-down from *P*. *falciparum* lysate using *Pf*ATM1-NTE as bait. Anti-6XHis antibodies and anti-*Pf*NBP35 serum were used to detect *Pf*ATM1-NTE and *Pf*NBP35, respectively in western blots. (**K**) *Ec*EngA used as negative control bait for the pull-down experiment in (**J**).
(PDF)

S8 Fig Multiple sequence alignment of CIA proteins. (**A**-**B**) ClustalW alignment of *Pf*NBP35 (A) and *Pf*CFD1 (B) with their homologs. NBP35 homologs: TgNbp35 from *T*. *gondii*, AtNbp35 from *A*. *thaliana*, HsNbp35 from *H*. *sapiens*, ScNbp35 from *S*. *cerevisiae*, and C.alb Nbp35 from *Candida albicans*. CFD1 homologs: HsNubp2 from *H*. *sapiens*, ScCfd1 from *S*. *cerevisiae*, and Calbcfd1 from *C*. *albicans*. Arrows mark the start and end of recombinant *Pf*NBP35 and *Pf*CFD1. The predicted transmembrane domain of *Pf*NBP35 is indicated by a navy blue dashed box.
(PDF)

S9 Fig *Pf*ATM1 activity and proteoliposomes. (**A**-**B**) ATPase activity of *Pf*ATM1 as a function of time (A) and protein concentration (B). (**C**) Change in intrinsic tryptophan fluorescence of apo-*Pf*ATM1 with increasing concentrations of GSSG. (**D**) Dynamic light scattering scan for determination of average size of reconstituted *Pf*ATM1-proteoliposomes. (**E**) The incorporation of *Pf*ATM1 confirmed by SDS-PAGE of proteoliposomes.
(PDF)

S1 Table List of sequences used for phylogeny of ATM1 in Fig 1B and S1A Fig. (XLSX)

S2 Table List of primers used throughout this study. The assigned numbers, descriptions and sequences are given. (XLSX)

S3 Table Raw values from the extracellular flux analyser measurements shown in Figs 3 and 4 as well as S4 Fig. (XLSX)

S4 Table Raw values from the gas chromatography-mass spectrometry (GC-MS) measurements shown in Figs 3 and 4 as well as S4 Fig. Note that the raw data has been deposited on the Yareta depository. It can be accessed under this doi: https://doi.org/10.26037/yareta:imf7o2y5kvghzefwecqsemjh3y
(XLSX)

S5 Table Raw values and analyses from the proteomic analysis shown in Fig 3 (*Tg*ATM1 downregulation). Note that the raw data has been deposited on the Yareta depository. It can be accessed under this doi: https://doi.org/10.26037/yareta:imf7o2y5kvghzefwecqsemjh3y
(XLSX)

S6 Table Raw values and analyses from the proteomic analysis shown in Fig 4 (*Tg*ATM1 overexpression). Note that the raw data has been deposited on the Yareta depository. It can be accessed under this doi: https://doi.org/10.26037/yareta:xe4xwdhnr5gyrlakhmvjc2et6y
(XLSX)
