# Supplementary material for: ATM1, an essential conserved transporter in Apicomplexa, bridges mitochondrial and cytosolic [Fe-S] biogenesis
Source: PLoS Pathog. 2024 Sep 30;20(9):e1012593. doi: 10.1371/journal.ppat.1012593 (PMC11476691; doi:10.1371/journal.ppat.1012593)
Supplement: S1 Fig — (A) Phylogenetic analysis of PfABCB2 (PfMDR2) and PfABCB6 (PfMDR6) (sequences in S1 Table). LG+R+F model was found to be the best substitution model predetermined by ATGC: SMS (Smart model selection) in PhyML 3. 0, visualised by iTOL software. Bootstrap support values (in red) and branch lengths of the unrooted tree are shown. Green arrow indicates a more recent common ancestor shared between PfABCB2 (PfMDR2) and eukaryotic HMTs. (B) Multiple sequence alignment of ATM1 homologs from apicomplexan parasites. ClustalW alignment of ATM1- like proteins of P. falciparum, Toxoplasma gondii and Cryptosporidium parvum with Saccharomyces cerevisiae ATM1, bacterial ATM1 of Novosphingobium aromaticivorans, fungal ATM1 of Chaetomium thermophilum, ATM3 (ABCB25) of Arabidopsis thaliana, and human ABCB7. The NTE, transmembrane region, insertion and CTD with conserved ATP-binding sites are indicated. Positions showing the extent of full-length recombinant PfATM1 (FL) and its CTD and NTE domains are marked. Black arrows and blue circles indicate GSSG binding sites in NaATM1 and GSH binding sites in ScATM1, respectively. [2Fe-2S]GS4 cluster coordination sites in ScATM1 and HsABCB7 are marked by grey and orange circles, respectively. Gatekeeper residues in NaATM1, CtATM1 and PfATM1 are boxed in blue. The TM6 helix of NaATM1 is indicated by a blue dashed line. The predicted α-helix (PSIPRED prediction) in the NTE of PfATM1 is shown by a red line and the hydrophobic stretch in this region is indicated by a green dashed line. Poly(Asn) in the NTE is indicated by a blue line. (PDF) [file ppat.1012593.s001.pdf]

A

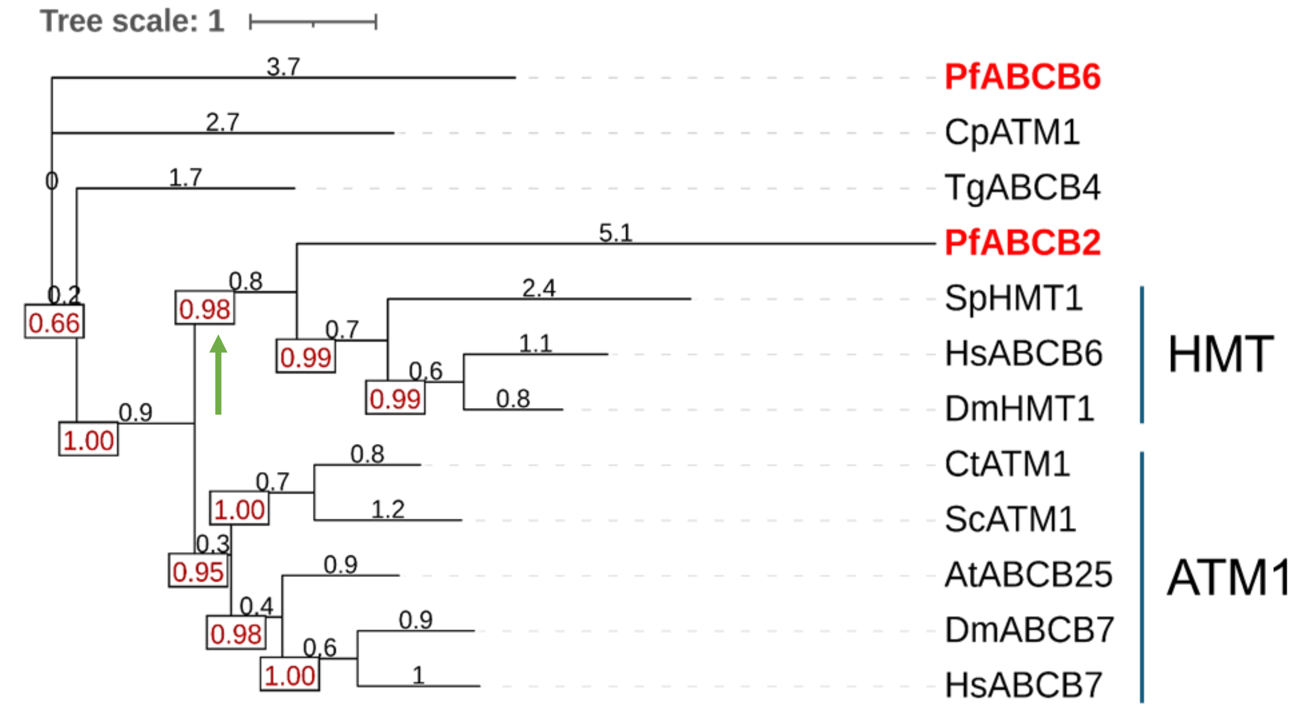

Tree scale: 1
3.7
2.7
1.7
5.1
2.4
1.1
0.8
0.8
1.2
0.9
0.9
1
0
0.2
0.66
0.98
0.8
0.7
0.99
0.6
0.99
0.9
1.00
0.7
1.00
0.3
0.95
0.4
0.98
0.6
1.00
PfABCB6
CpATM1
TgABCB4
PfABCB2
SpHMT1
HsABCB6
DmHMT1
CtATM1
ScATM1
AtABCB25
DmABCB7
HsABCB7
HMT
ATM1

B

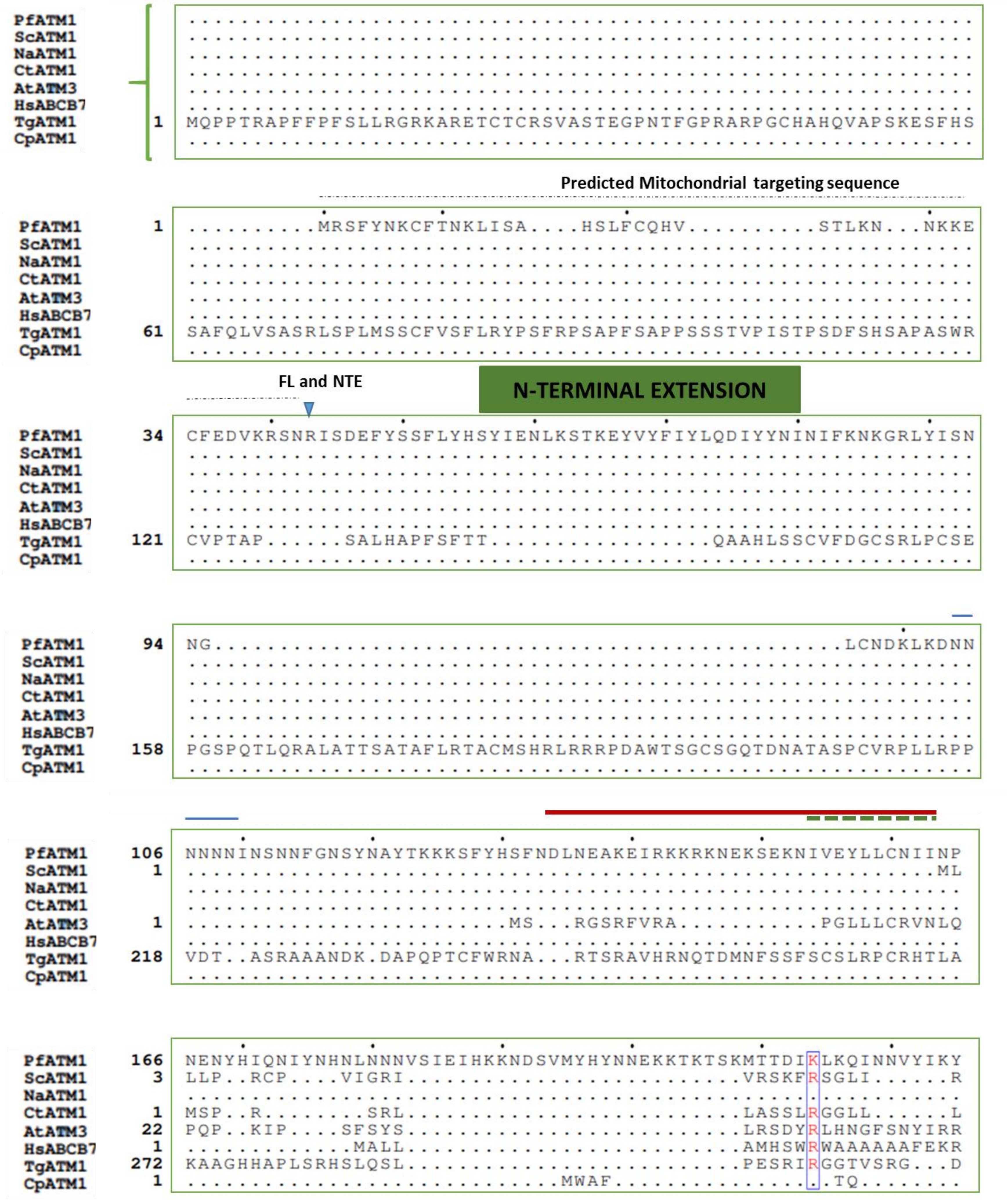

PfATM1
ScATM1
NaATM1
CtATM1
AtATM3
HsABCB7
TgATM1
CpATM1
1 MQPPTRAPFFPFSLLRGRKARETCTCRSVASTEGPNTFGPRARPGCHAHQVAPSKESFHS
Predicted Mitochondrial targeting sequence
1 ..........MRSFYNKCFTNKLISA....HSLFCQHV..........STLKN...NKKE
61 SAFQLVSASRLSPLMSSCFVSFLRYPSFRPSAPFSAPPSSSTVPISTPSDFSHSAPASWR
FL and NTE
N-TERMINAL EXTENSION
34 CFEDVKRSNRISDEFYSSFLYHSYIENLKSTKEYVYFIYLQDIYYNINIFKNKGRLYISN
121 CVPTAP......SALHAPFSFTT...............QAAHLSSCVFDGCSRLPCSE
94 NG...LCNDKLKDNN
158 PGSPQTLQRALATTSATAFLRTACMSHRLRRRPDAWTSGCSGQTDNATASPCVRPLLRPP
106 NNNNINSNNFGNSYNAYTKKKSFYHSFNDLNEAKEIRKKRKNEKSEKNIVEYLLCNIINP
1 ML
1 MS...RGSRFVRA.........PGLLLCRVNLQ
218 VDT..ASRAAANDK.DAPQPTCFWRNA...RTSRAVHRNQTDMNFSSFSCSLRPCRHTLA
166 NENYHIQNIYNHNLNNNVSIEIHKKNDSVMYHYNNEKKTKTSKMTTDIKLKQINNVYIKY
3 LLP..RCP....VIGRI....VRSKFRSGLI......R
1 MSP..R........SRL....LASSLRGGLL......L
22 PQP..KIP....SFSYS....LRSDYRLHNGFSNYIRR
1 ....MALL....AMHSWRWAAAAAAFEKR
272 KAAGHHAPLSRHSLQSL....PESRIRGGTVSRG...D
1 ....MWAF....TQ

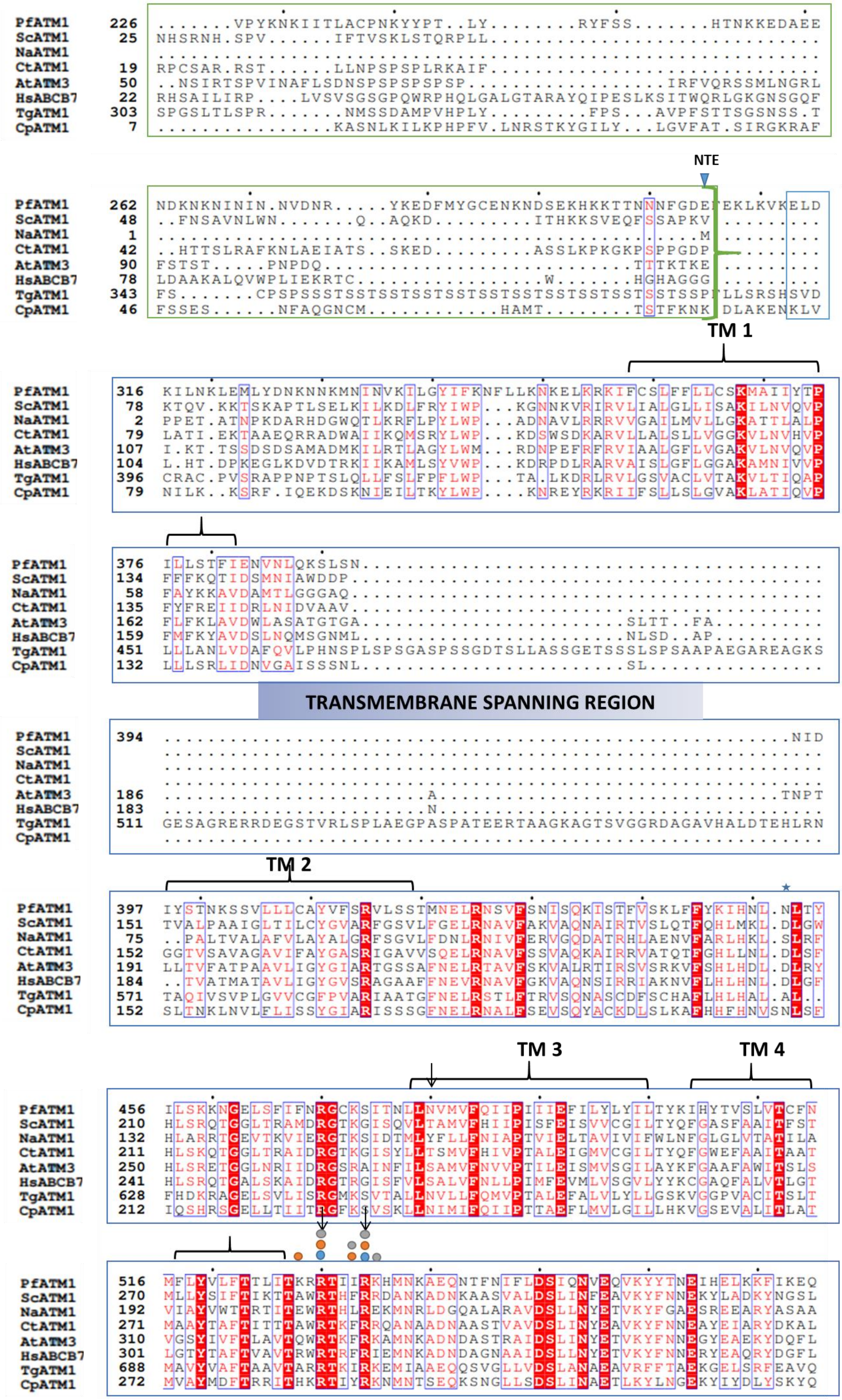

PfATM1
ScATM1
NaATM1
CtATM1
AtATM3
HsABCB7
TgATM1
CpATM1
NTE
TM 1
TRANSMEMBRANE SPANNING REGION
TM 2
TM 3
TM 4

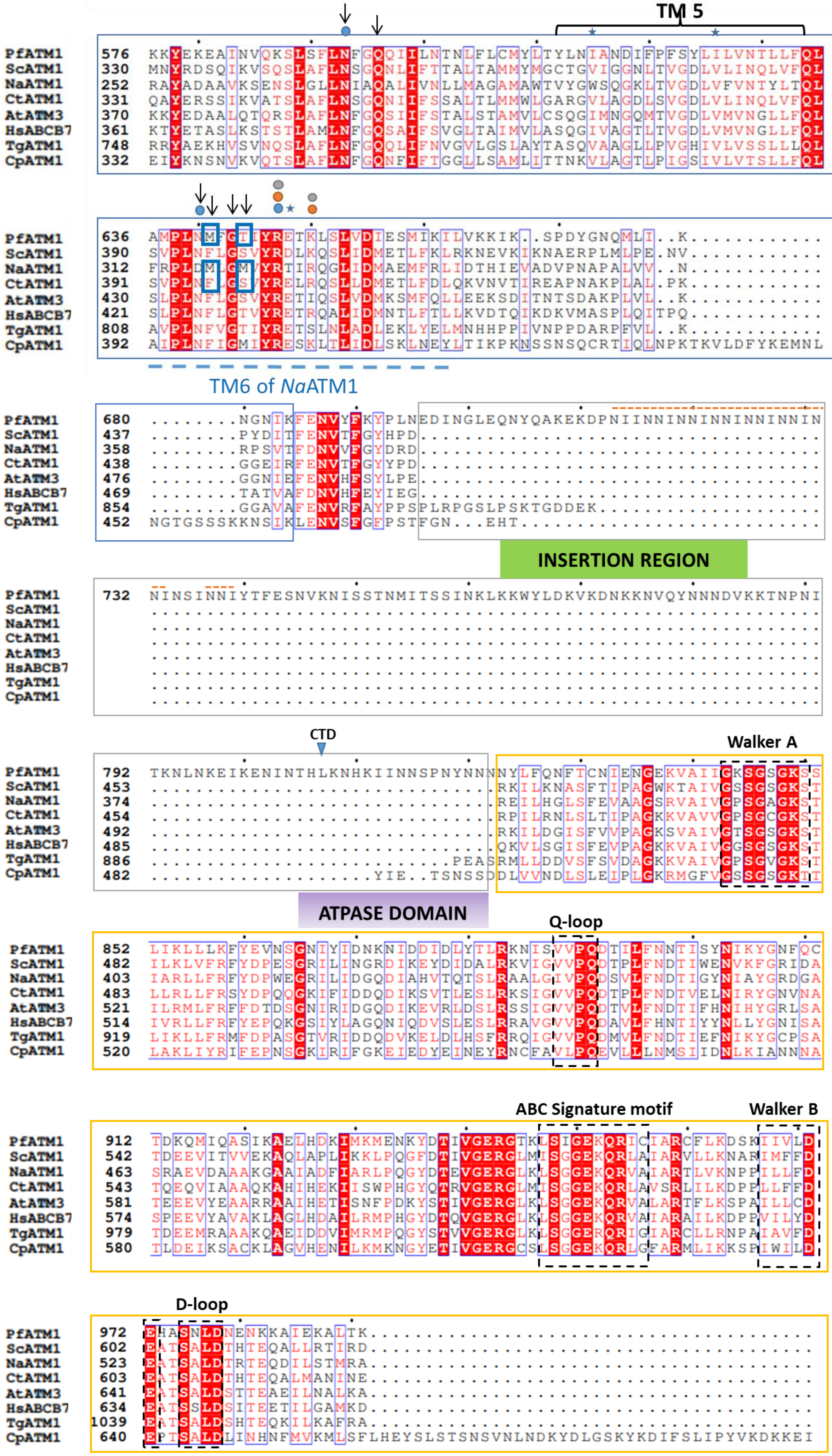
TM 5
TM6 of NaATM1
INSERTION REGION
CTD
Walker A
ATPASE DOMAIN
Q-loop
ABC Signature motif
Walker B
D-loop
PfATM1
ScATM1
NaATM1
CtATM1
AtATM3
HsABCB7
TgATM1
CpATM1

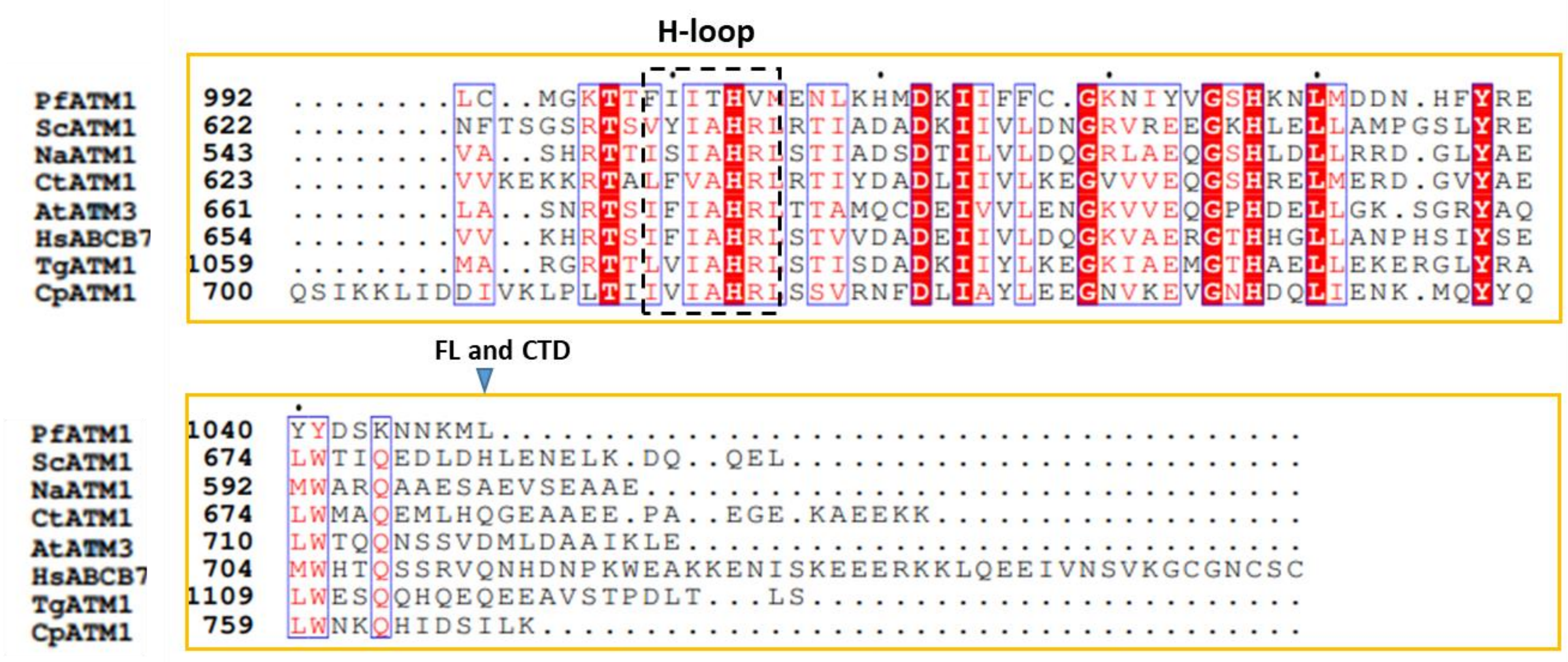

GSH binding sites in *Sc*ATM1 (Srinivasan et al., 2014)

GSSG binding sites in *Na*ATM1 (Fan et al., 2020)

[2Fe-2S]GS_4 cluster coordination sites in *Sc*ATM1 (Qi et al., 2014)

[2Fe-2S]GS_4 cluster coordination sites in *Hs*ABCB7 (Qi et al., 2014)

★ HsABCB7 mutants Linked to X-linked sideroblastic anaemia (Hooghe et al., 2012)

Poly(Asn) in *Pf*ATM1

Gatekeeper residues (Li et al., 2022)

Hydrophobic stretch in NTE of *Pf*ATM1 (Kyte-Doolittle hydropathy plot)

Predicted α-helix in NTE of *Pf*ATM1 (PSIPRED prediction)

TM6 helix of *Na*ATM1
