# Supplementary material for: ATM1, an essential conserved transporter in Apicomplexa, bridges mitochondrial and cytosolic [Fe-S] biogenesis
Source: PLoS Pathog. 2024 Sep 30;20(9):e1012593. doi: 10.1371/journal.ppat.1012593 (PMC11476691; doi:10.1371/journal.ppat.1012593)
Supplement: S2 Fig — (A) Cartoon scheme showing the principle of the conditional downregulation strategy employed for TgATM1 based on dimerizable Cre recombinase (DiCre) and mRNA destabilization. The U1-recognition site moves closer to the stop codon upon activation of the DiCre and causes destabilization and degradation of the mRNA resulting in reduced expression of TgATM1. (B) Genomic integration PCR probing the parental line (DiCreU1) and TgATM1-Ty-U1 parasites to validate the integration of the intended construct, including a Ty-tag, a U1 recognition site, loxP sites and a selection cassette, in the 3′UTR of TgATM1. The approximate primer binding sites are given in the scheme and primers are listed in S2 Table. (PDF) [file ppat.1012593.s002.pdf]

A

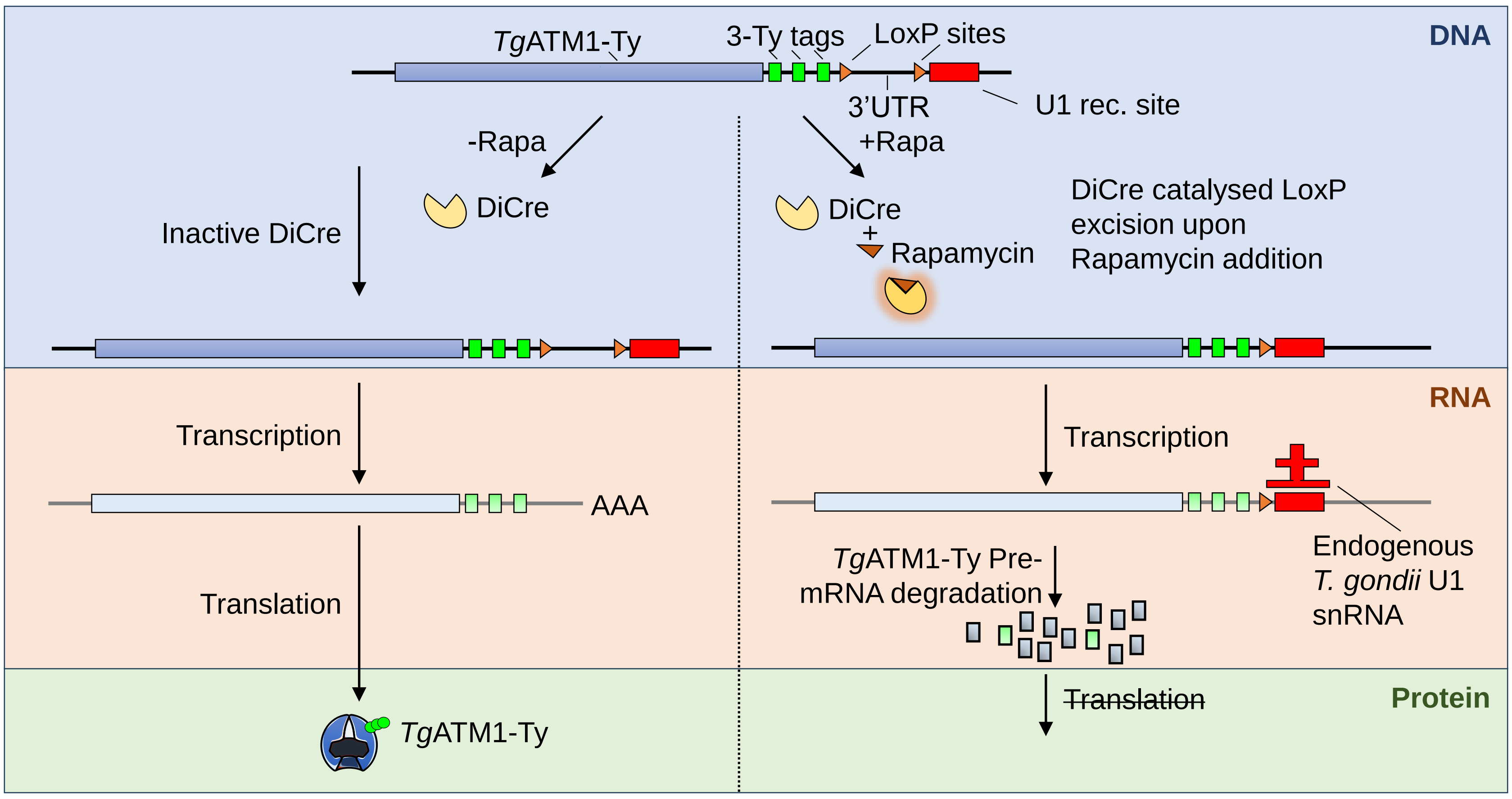
DNA
*Tg*ATM1-Ty
3-Ty tags
LoxP sites
3'UTR
U1 rec. site
-Rapa
+Rapa
DiCre
Inactive DiCre
DiCre
+
Rapamycin
DiCre catalysed LoxP excision upon Rapamycin addition
RNA
Transcription
AAA
Translation
Transcription
*Tg*ATM1-Ty Pre-mRNA degradation
Endogenous *T. gondii* U1 snRNA
Protein
*Tg*ATM1-Ty
~~Translation~~

B

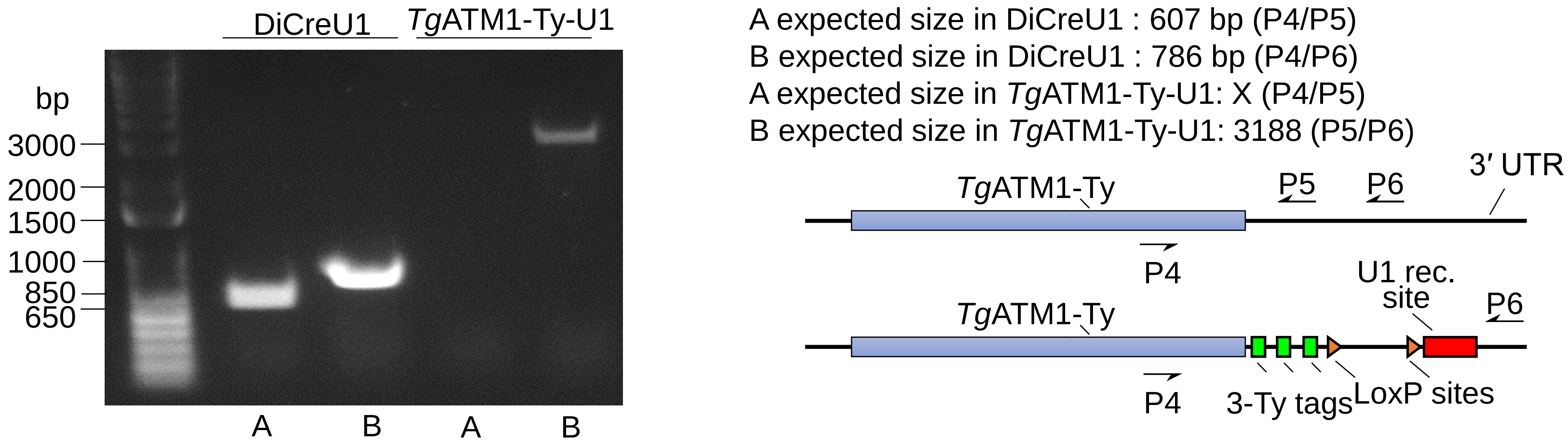
DiCreU1
*Tg*ATM1-Ty-U1
bp
3000
2000
1500
1000
850
650
A
B
A
B
A expected size in DiCreU1 : 607 bp (P4/P5)
B expected size in DiCreU1 : 786 bp (P4/P6)
A expected size in *Tg*ATM1-Ty-U1: X (P4/P5)
B expected size in *Tg*ATM1-Ty-U1: 3188 (P5/P6)
*Tg*ATM1-Ty
P5
P6
3′ UTR
P4
*Tg*ATM1-Ty
U1 rec. site
P6
P4
3-Ty tags
LoxP sites
