# Supplementary figures and images for: ATM1, an essential conserved transporter in Apicomplexa, bridges mitochondrial and cytosolic [Fe-S] biogenesis
Source: PLoS Pathog. 2024 Sep 30;20(9):e1012593. doi: 10.1371/journal.ppat.1012593 (PMC11476691; doi:10.1371/journal.ppat.1012593)

**A**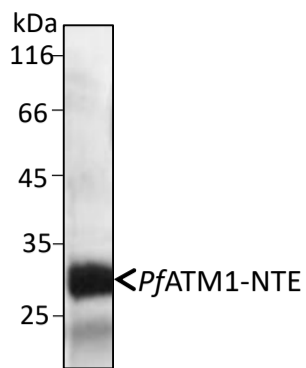**B**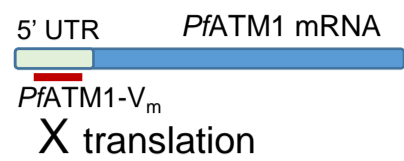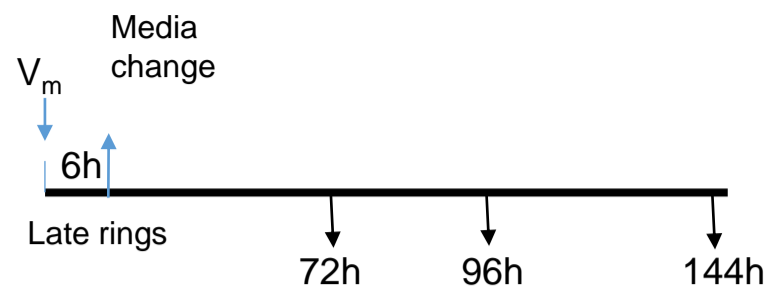**C**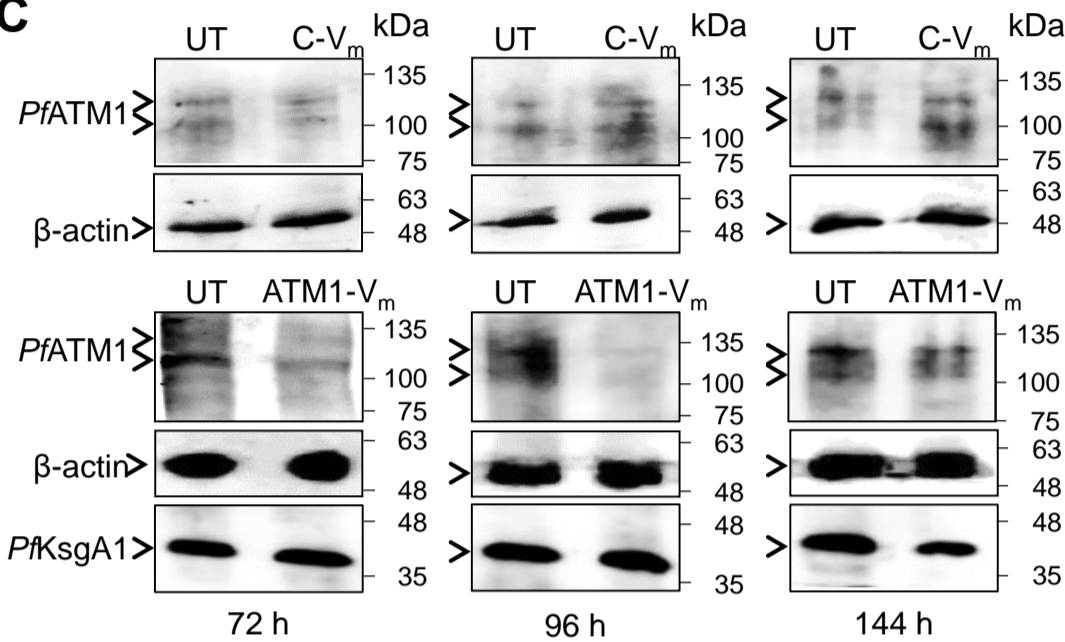**D**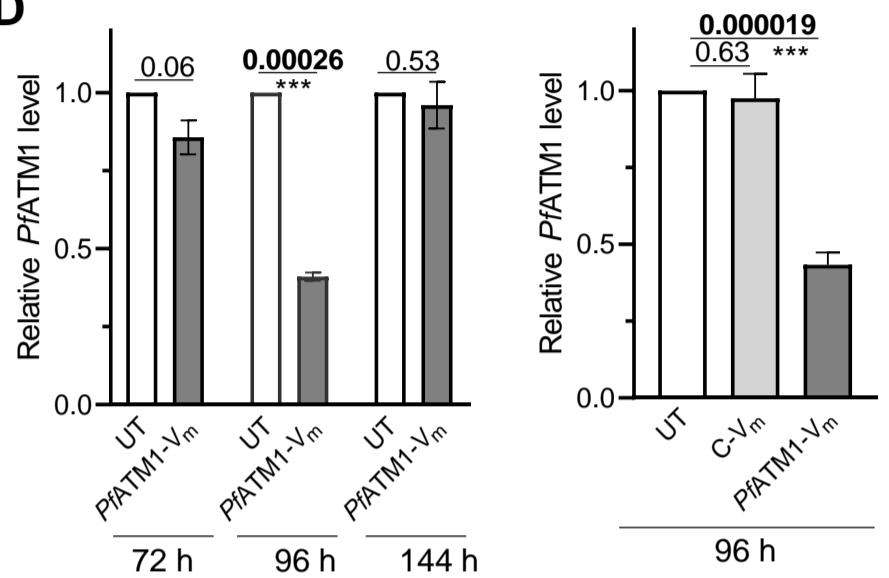**E**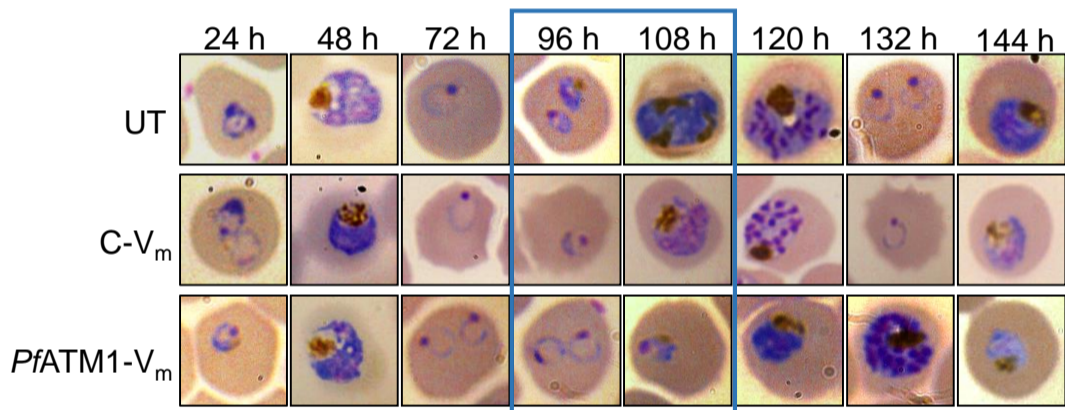**F**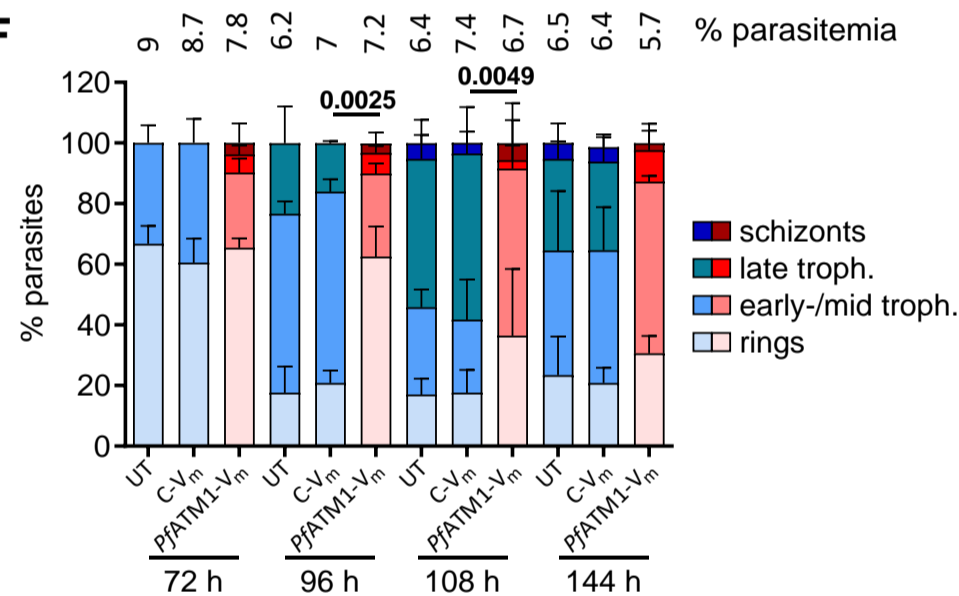**G**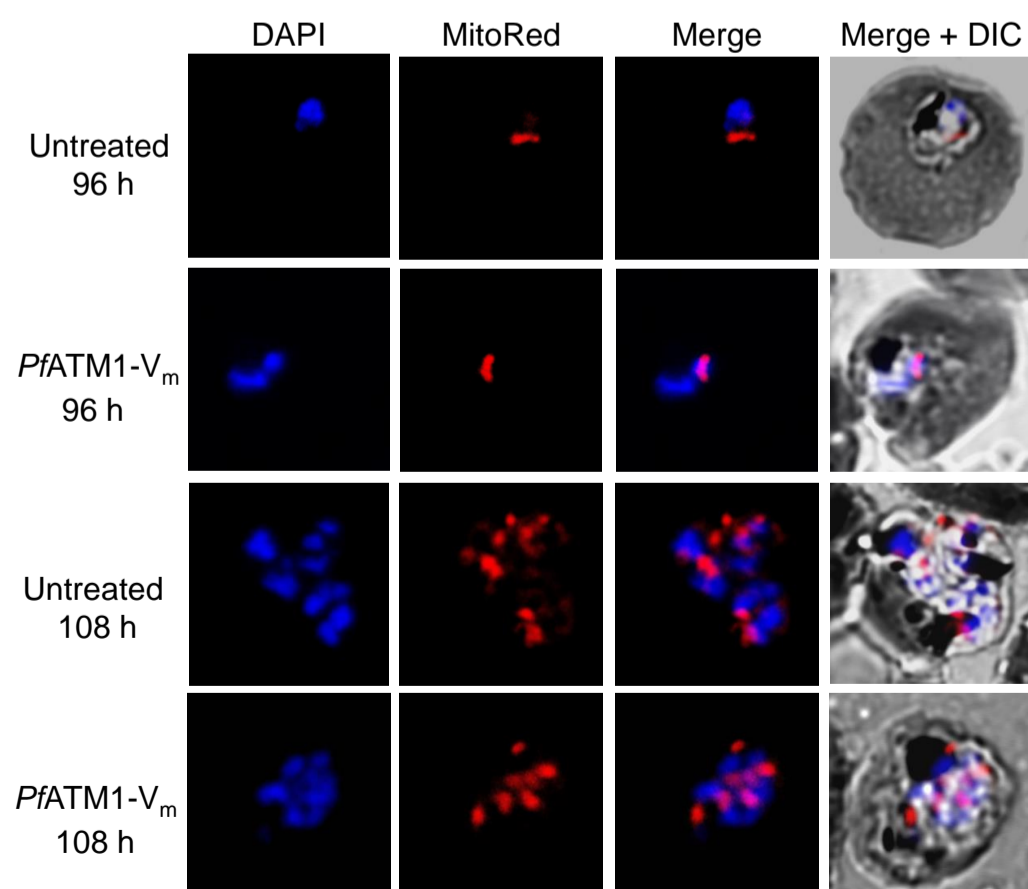**H**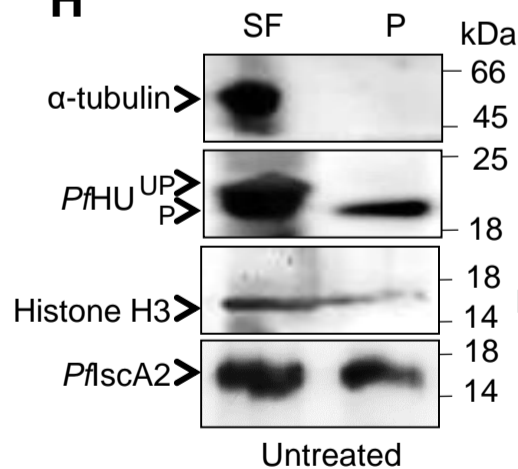**I**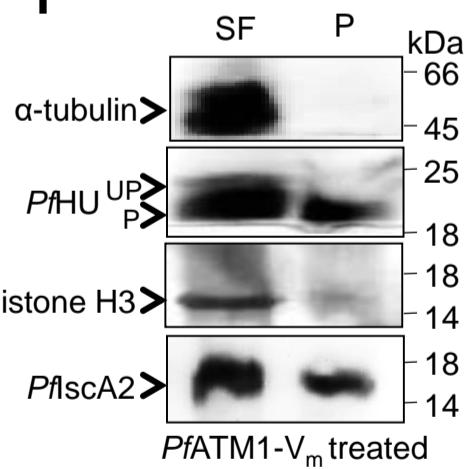**J**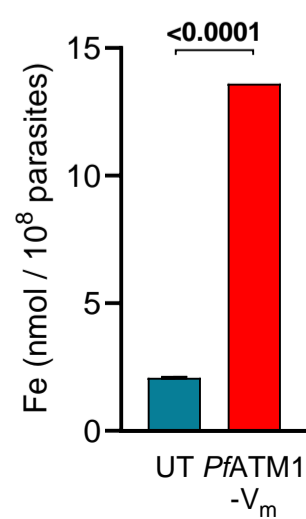**K**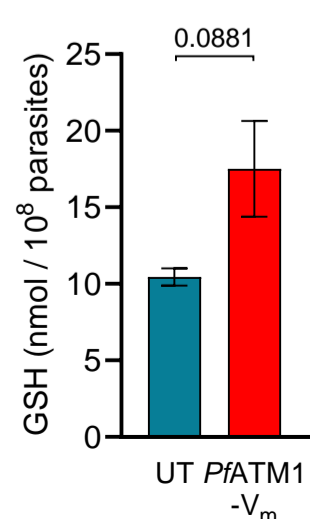

Supplement: S3 Fig — (A) Coomassie-stained SDS-PAGE of purified ~33 kDa PfATM1-NTE. (B) Strategy for blocking translation of PfATM1 using PfATM1-Vm. (C) Time-dependent expression of PfATM1 in untreated (UT), control morpholino (C-Vm) or PfATM1-Vm treated parasites at 72 hours (i), 96 hours (ii) and 144 hours (iii) post-treatment. Actin served as loading control. (D) Quantitative analysis of western blots after Vm treatment. PfATM1 levels at 72, 96, and 144 hours post-treatment in untreated and PfATM1-Vm treated cells were normalized with actin and plotted relative to levels in untreated cells (left). Two biological replicates were analysed by two-tailed Student’s t-test; mean and SD are plotted. PfATM1 levels from untreated, C-Vm treated and PfATM1-Vm treated cells were compared at 96 hours post-treatment (right). Mean and SD of three biological replicates are plotted. Significant differences (p <0.05) are in bold. (E) Giemsa-stained parasites representing predominant stages from untreated, C-Vm and PfATM1-Vm treated sets at different times post-treatment. (F) Parasite stages as % of total parasites in the second and third infection cycle post-treatment. P values (t-test) are shown for comparison of ring stage between control Vm and PfATM1-Vm at 96 hours, and for late trophozoites between control Vm and PfATM1-Vm at 108 hours. Significant differences (p <0.05) are highlighted in bold. Parasitemia (%) is given at the top of the bars. (G) Confocal microscopy after staining PfATM1-Vm treated and untreated cells at 96 and 108 hours post-treatment. Cells were stained with DAPI and Mitotracker Red to detect any changes in mitochondrial morphology. Matching parasite stages were selected for comparison. (H) and (I) Western blot of organellar fraction from control untreated (H) and PfATM1-Vm-treated cells (I) probed with antibodies against mitochondrial (PfIscA2), apicoplast (PfHU), nuclear (Histone H3) and cytosolic (α-tubulin) proteins. The cytosolic marker protein α-tubulin was not seen in th [file ppat.1012593.s003.pdf]

**A**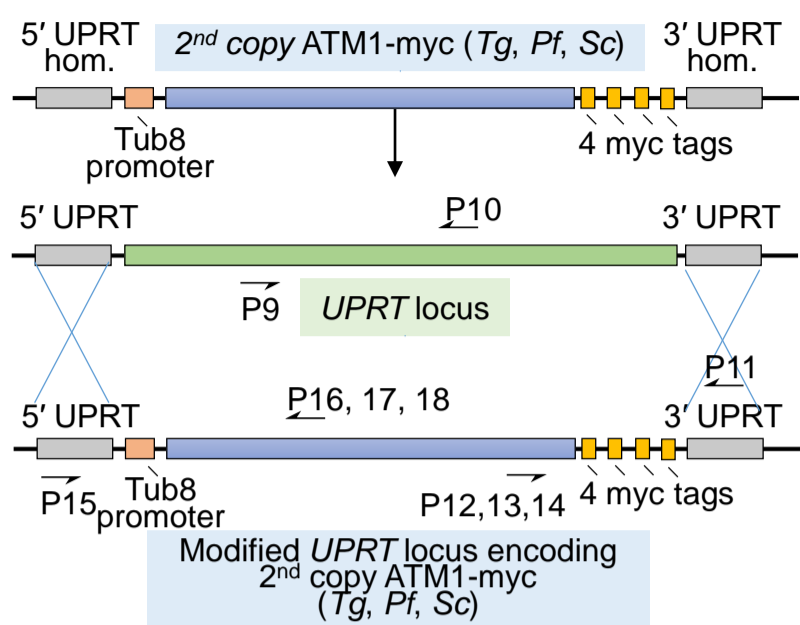**B**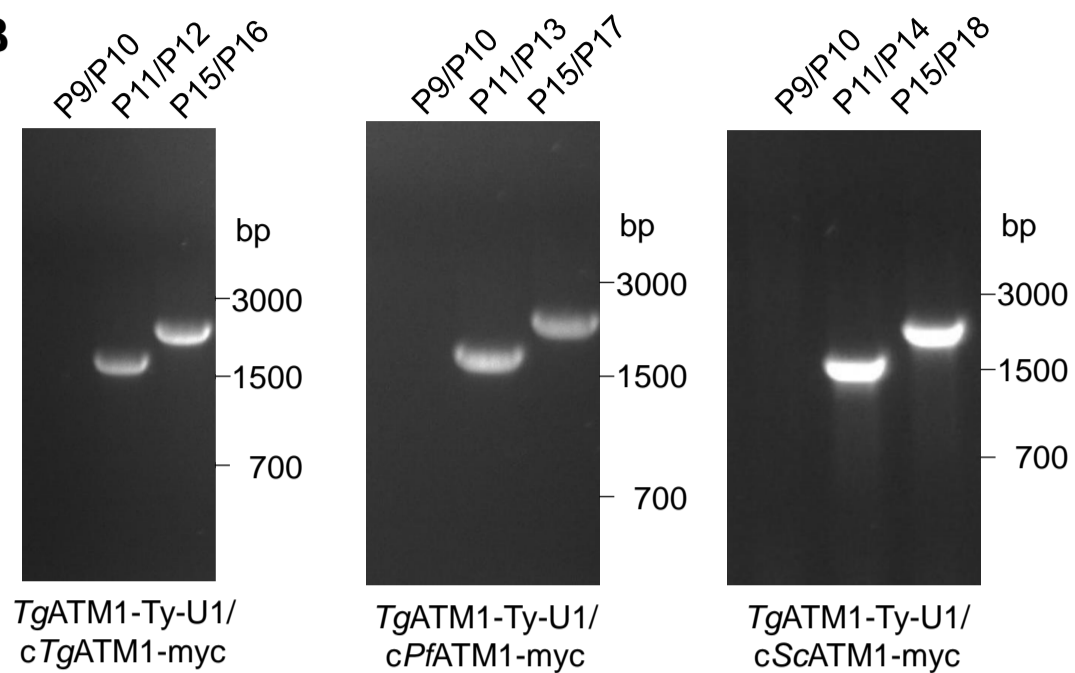**C**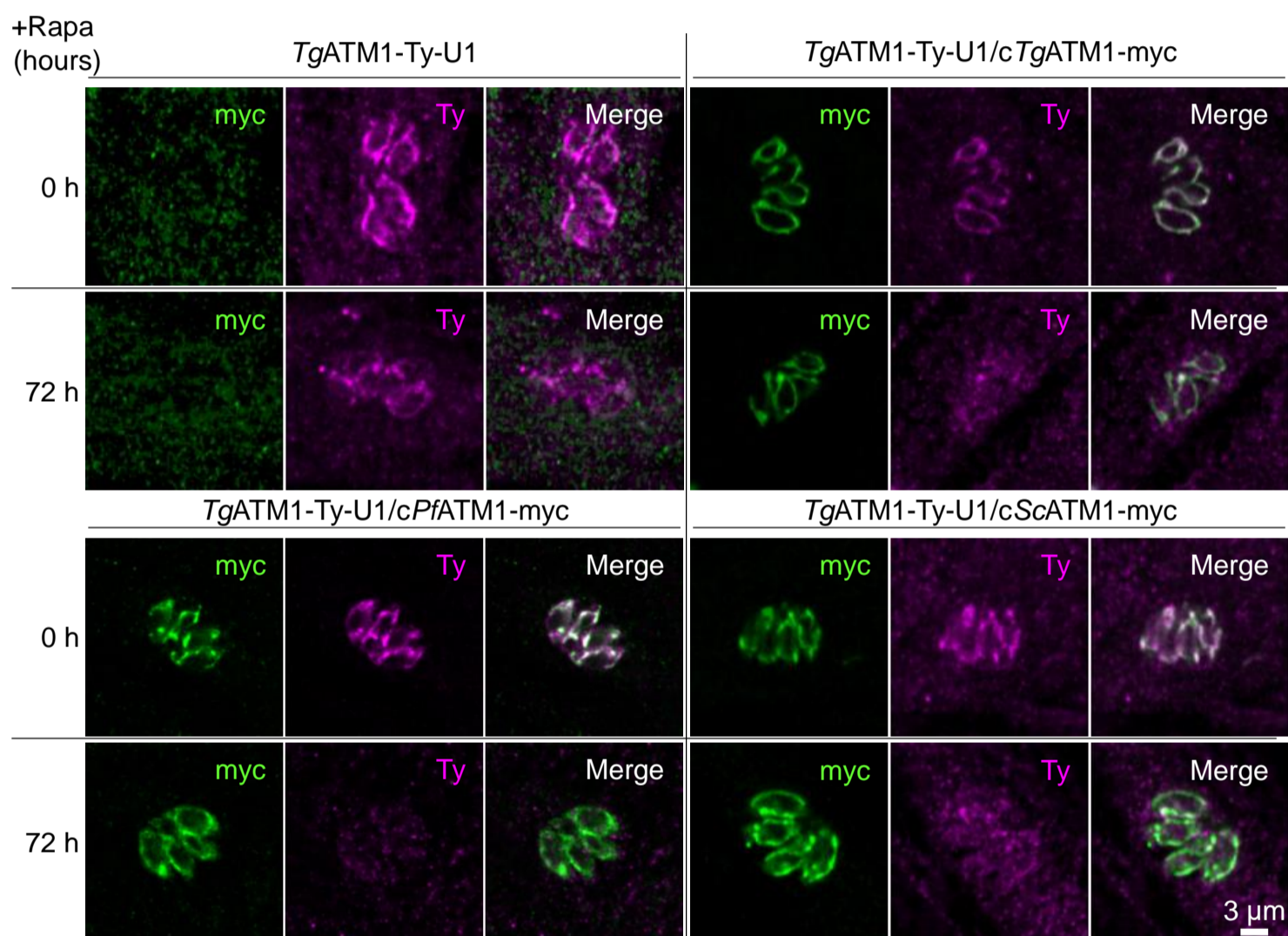**D**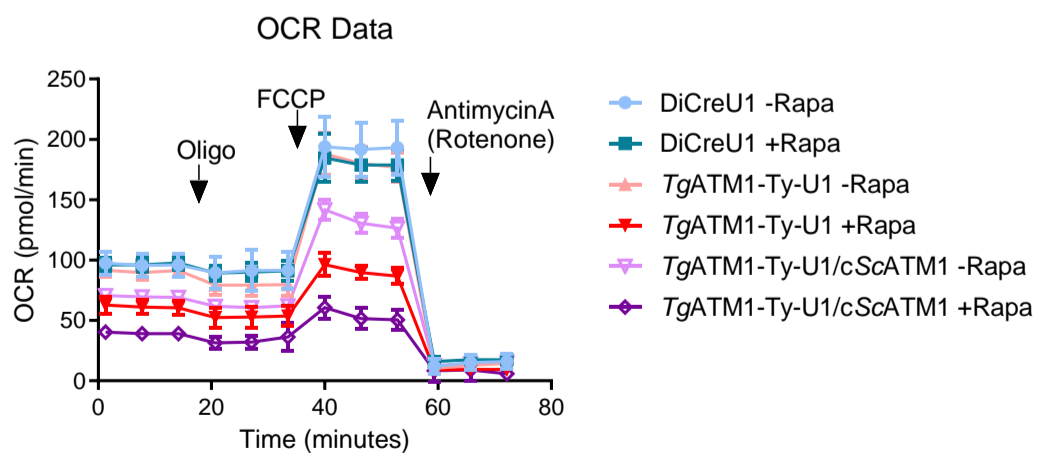**E**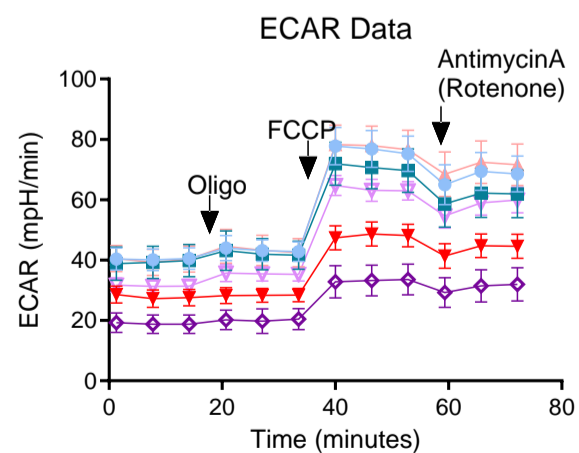

Supplement: S4 Fig — (A) Scheme visualizing the strategy to insert second copies of myc-tagged ATM1s from Toxoplasma, Plasmodium and yeast (Tg, Pf and Sc, respectively) into the uracil phosphoribsyltransferase (UPRT) locus of T. gondii. (B) Modification of the UPRT locus was validated by genomic PCR. The approximate binding sites of the employed primers are shown in scheme A and sequences listed in S2 Table. (C) Immunofluorescence assay of TgATM1-Ty-U1 parasites and TgATM1-Ty-U1 parasites expressing a second copy of myc-tagged Toxoplasma- (TgATM1-Ty-U1/cTgATM1-myc), Plasmodium- (TgATM1-Ty-U1/cPfATM1-myc) or yeast ATM1 (TgATM1-Ty-U1/cScATM1-myc). Parasites were stained with anti-Ty and anti-myc antibodies and merged signals are shown. Images are representative of three independent biological replicates. (D and E) Representative traces from an extracellular flux analysis following 0 or 72 hours of Rapa treatment of parental (DiCreU1), uncomplemented TgATM1-Ty-U1 parasites or TgATM1-Ty-U1 parasites expressing a second copy of myc-tagged yeast ATM1 (TgATM1-Ty-U1/cScATM1-myc). The oxygen consumption rate (OCR, D) and extracellular media acidification rate (ECAR, E) are shown. The legend shown in D applies to both panels. Other overexpressing strains behaved similarly–see S3 Table and Fig 4E and 4F. (PDF) [file ppat.1012593.s004.pdf]

**A**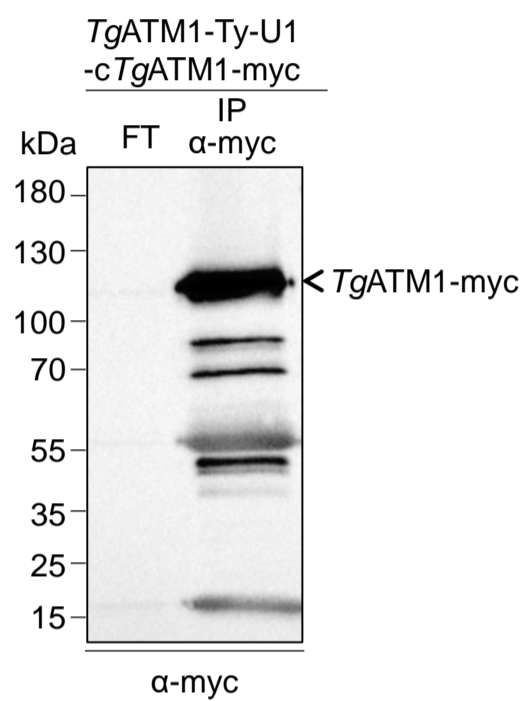**B**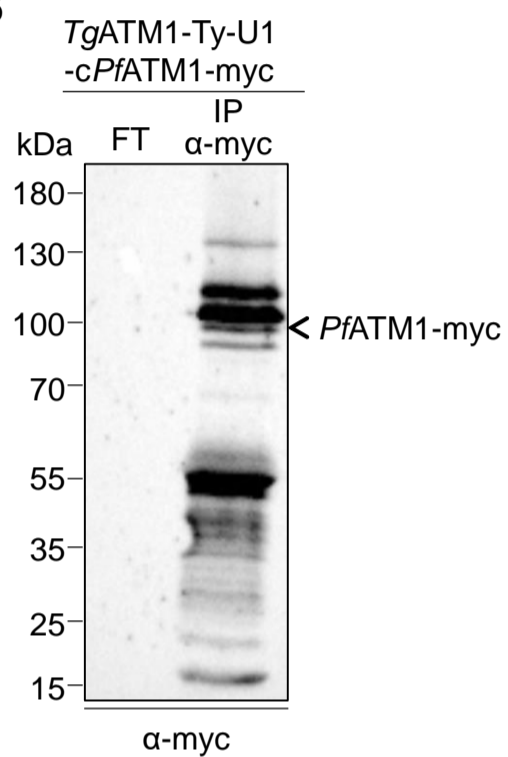**C**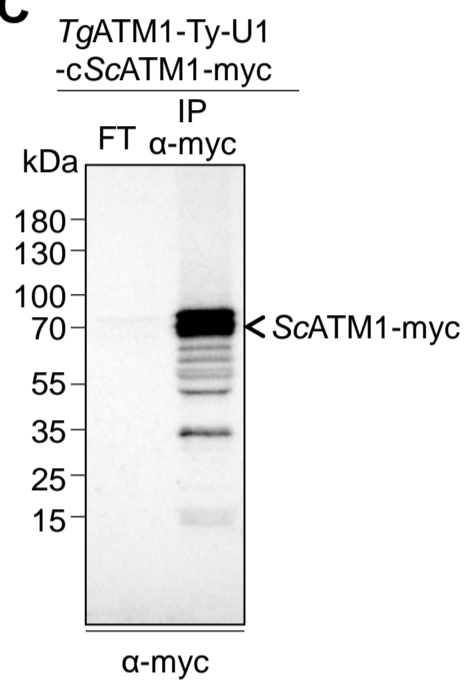**D**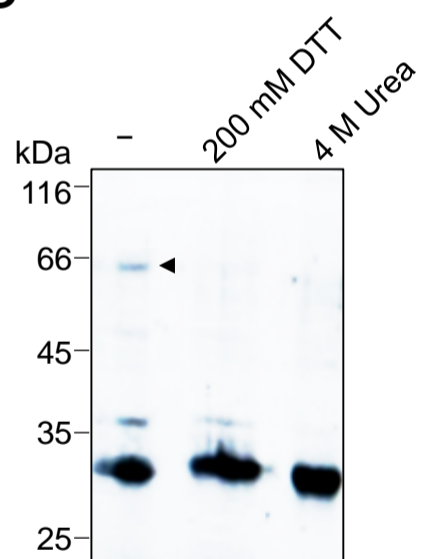**E**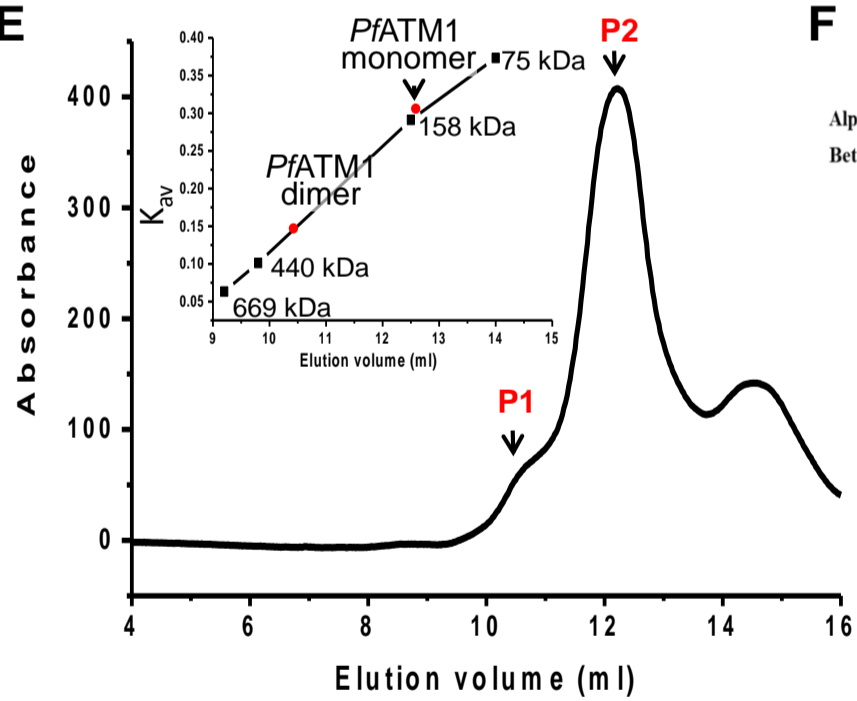**F**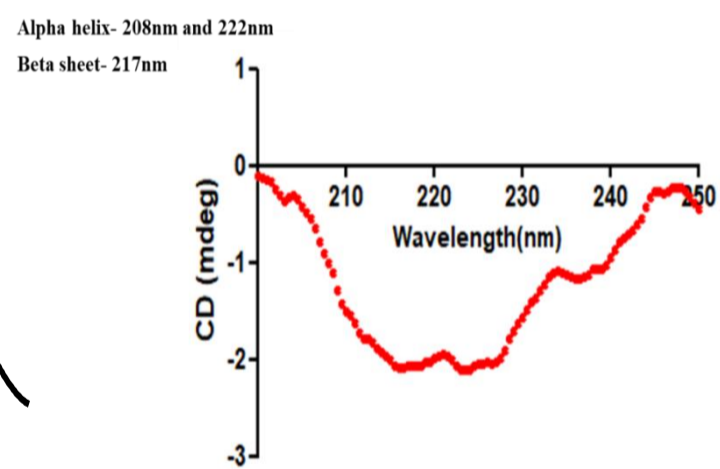

Supplement: S5 Fig — (A-C) Controls for Co-immunoprecipitation assay shown in Fig 5A-5C. Western blots as described in Fig 5A-5C but membranes were probed with anti-myc antibody. TgATM1-myc (A), PfATM1-myc (B) and ScATM1-myc (C) are indicated. Blots are representative of three independent biological replicates. (D) Recombinant PfATM1-CTD dimer breaks down into monomers upon treatment with DTT and urea. (E) Size exclusion chromatography on S200 column for purification of PfATM1 separates dimeric (P1) and monomeric protein with some degradation (P2). Absorbance at 280 nm is plotted against elution volume. Coomassie-stained SDS-PA gel for P1 and P2 is shown in Fig 5F. Inset, molecular weight standard plot for S200. (F) CD spectra of purified PfATM1 homodimer. (PDF) [file ppat.1012593.s005.pdf]

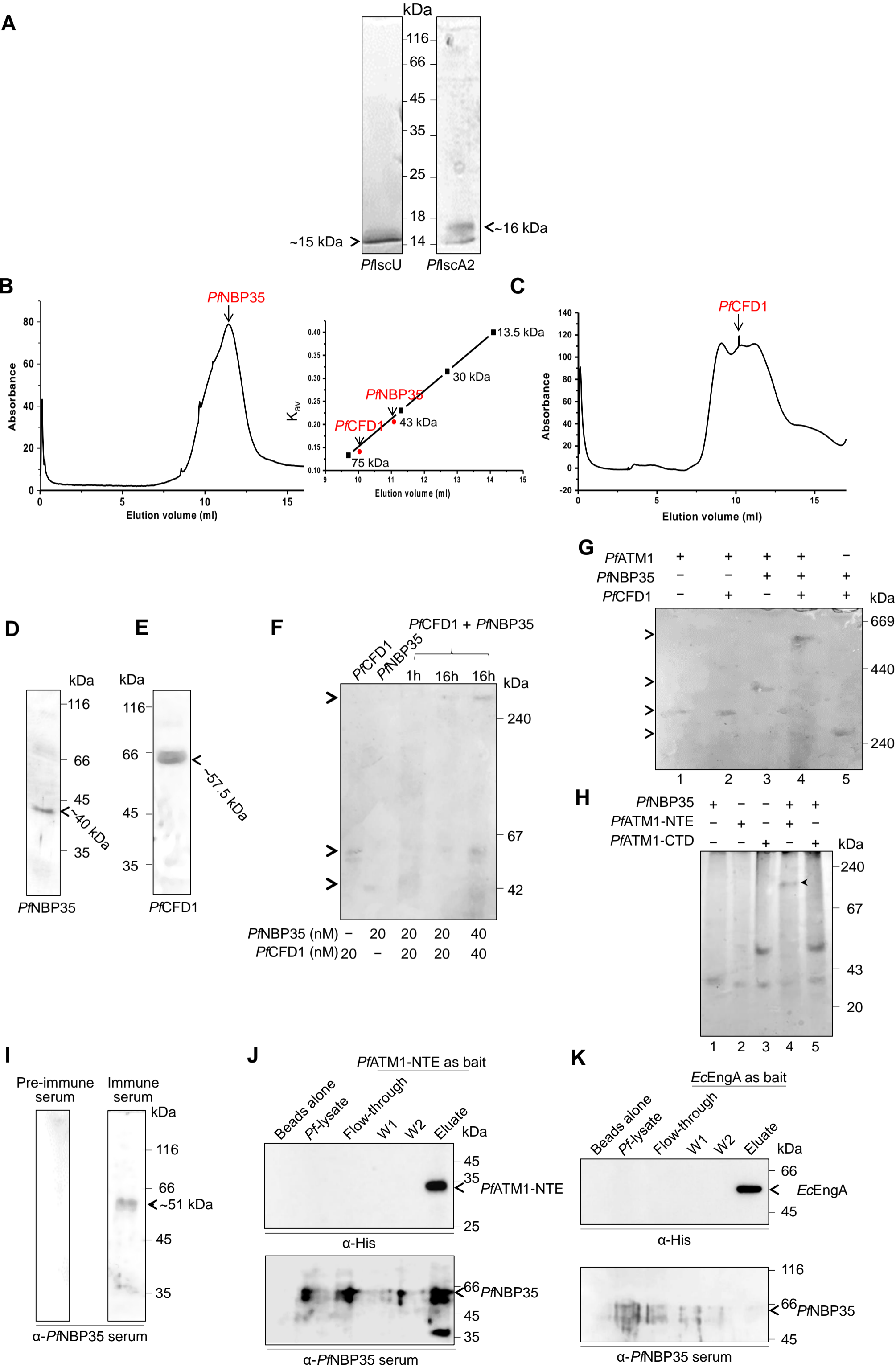

Supplement: S7 Fig — (A) PfIscU and PfIscA2 after thrombin cleavage for removal of GST from the purified fusion proteins. (B-C) Size exclusion chromatography of partially purified PfNBP35 (B) and PfCFD1 (C) through S75 column. Absorbance at 280 nm is plotted against elution volume. Inset, Molecular weight standard plot for S75. (D) Coomassie-stained SDS-PAGE of purified recombinant PfNBP35 (~40 kDa) and (E) Purified recombinant PfCFD1 (~57.5 kDa). (F) 6–10% native PAGE to detect complexation of PfNBP35 and PfCFD1. (G) 6–8% native PAGE to detect in vitro complexation of PfATM1 dimer (lane 1) with PfCFD1 (lane 2) and PfNBP35 (lane 3). The PfNBP35-PfCFD1 heterotetramer (lane 5) complexed with the PfATM1 dimer (lane 4). (H) 10–12% native PAGE to detect in vitro complexation of PfATM1-NTE and PfATM1-CTD with PfNBP35. (I) Anti-PfNBP35 serum detects a specific band of the expected size of ~51 kDa in the parasite lysate. (J) Pull-down from P. falciparum lysate using PfATM1-NTE as bait. Anti-6XHis antibodies and anti-PfNBP35 serum were used to detect PfATM1-NTE and PfNBP35, respectively in western blots. (K) EcEngA used as negative control bait for the pull-down experiment in (J). (PDF) [file ppat.1012593.s007.pdf]

A

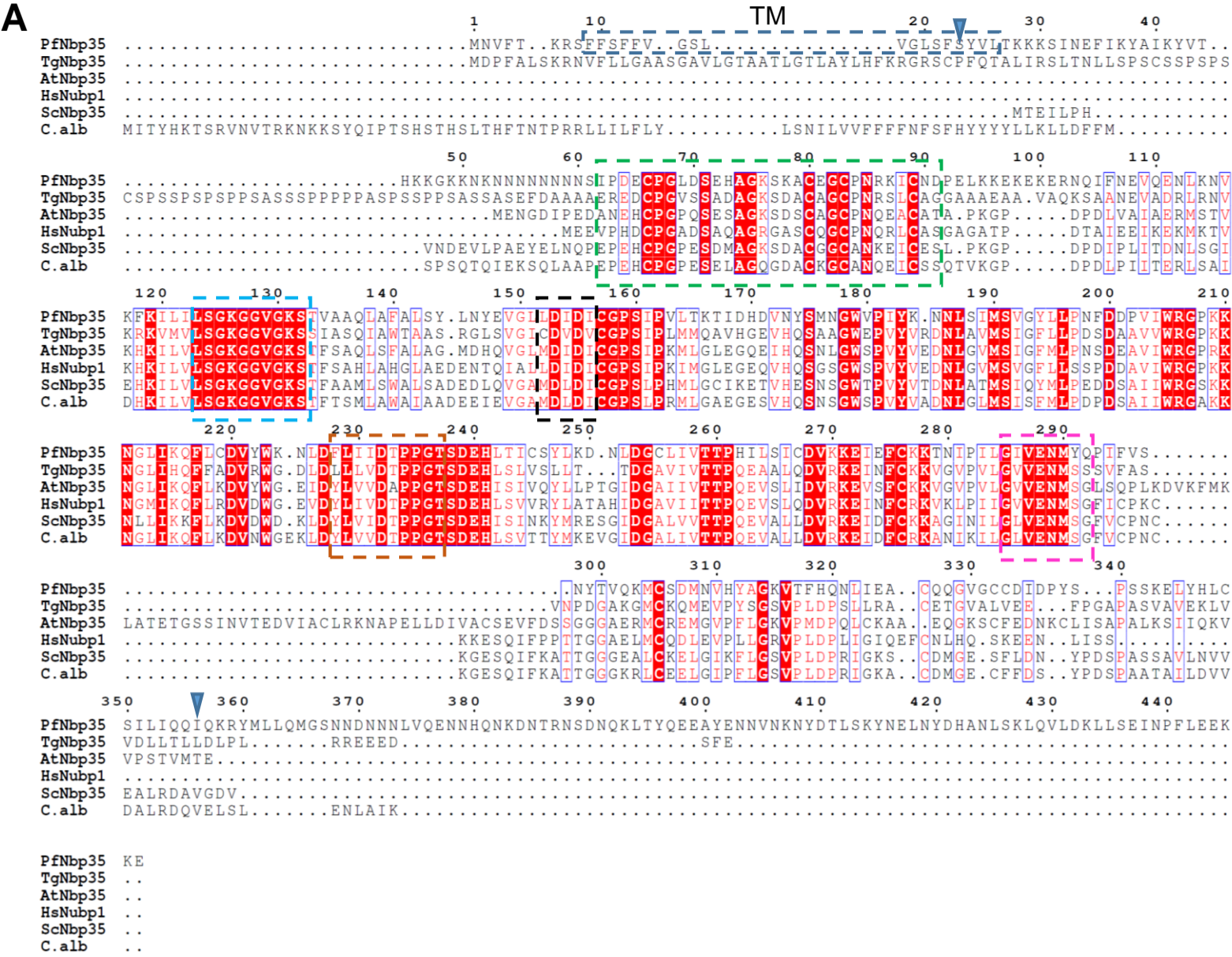

B

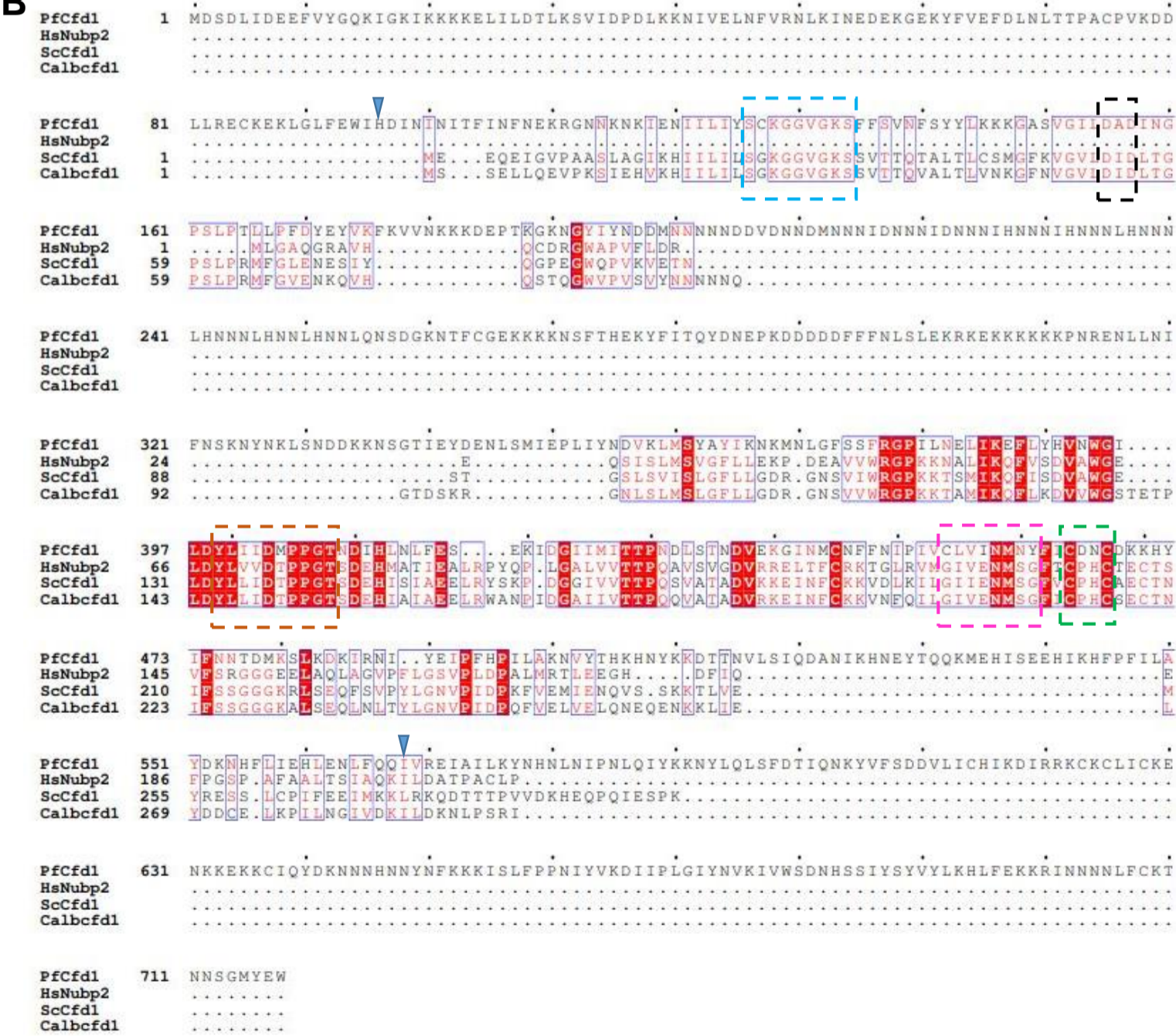

Supplement: S8 Fig — (A-B) ClustalW alignment of PfNBP35 (A) and PfCFD1 (B) with their homologs. NBP35 homologs: TgNbp35 from T. gondii, AtNbp35 from A. thaliana, HsNbp35 from H. sapiens, ScNbp35 from S. cerevisiae, and C.alb Nbp35 from Candida albicans. CFD1 homologs: HsNubp2 from H. sapiens, ScCfd1 from S. cerevisiae, and Calbcfd1 from C. albicans. Arrows mark the start and end of recombinant PfNBP35 and PfCFD1. The predicted transmembrane domain of PfNBP35 is indicated by a navy blue dashed box. (PDF) [file ppat.1012593.s008.pdf]

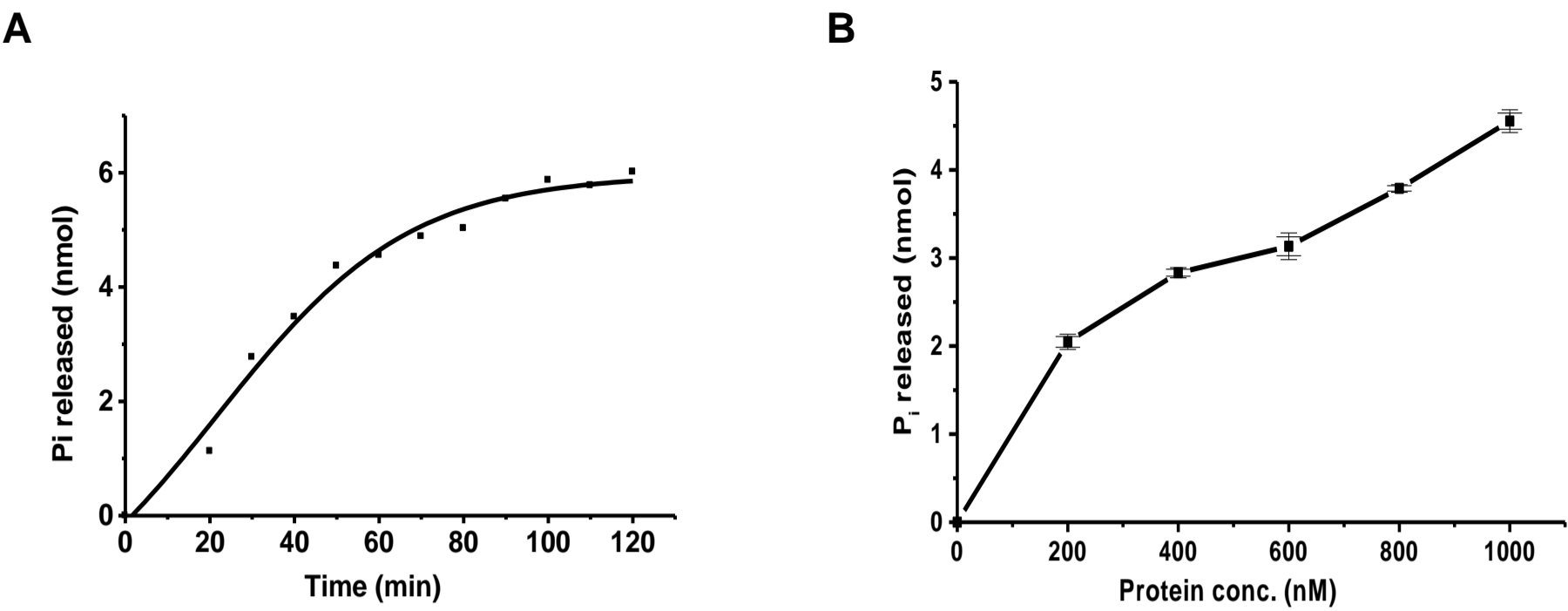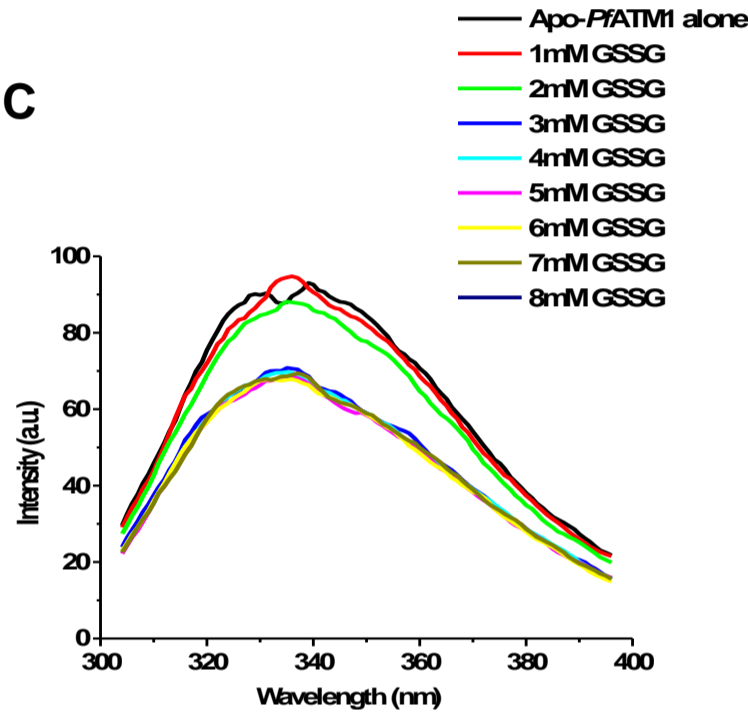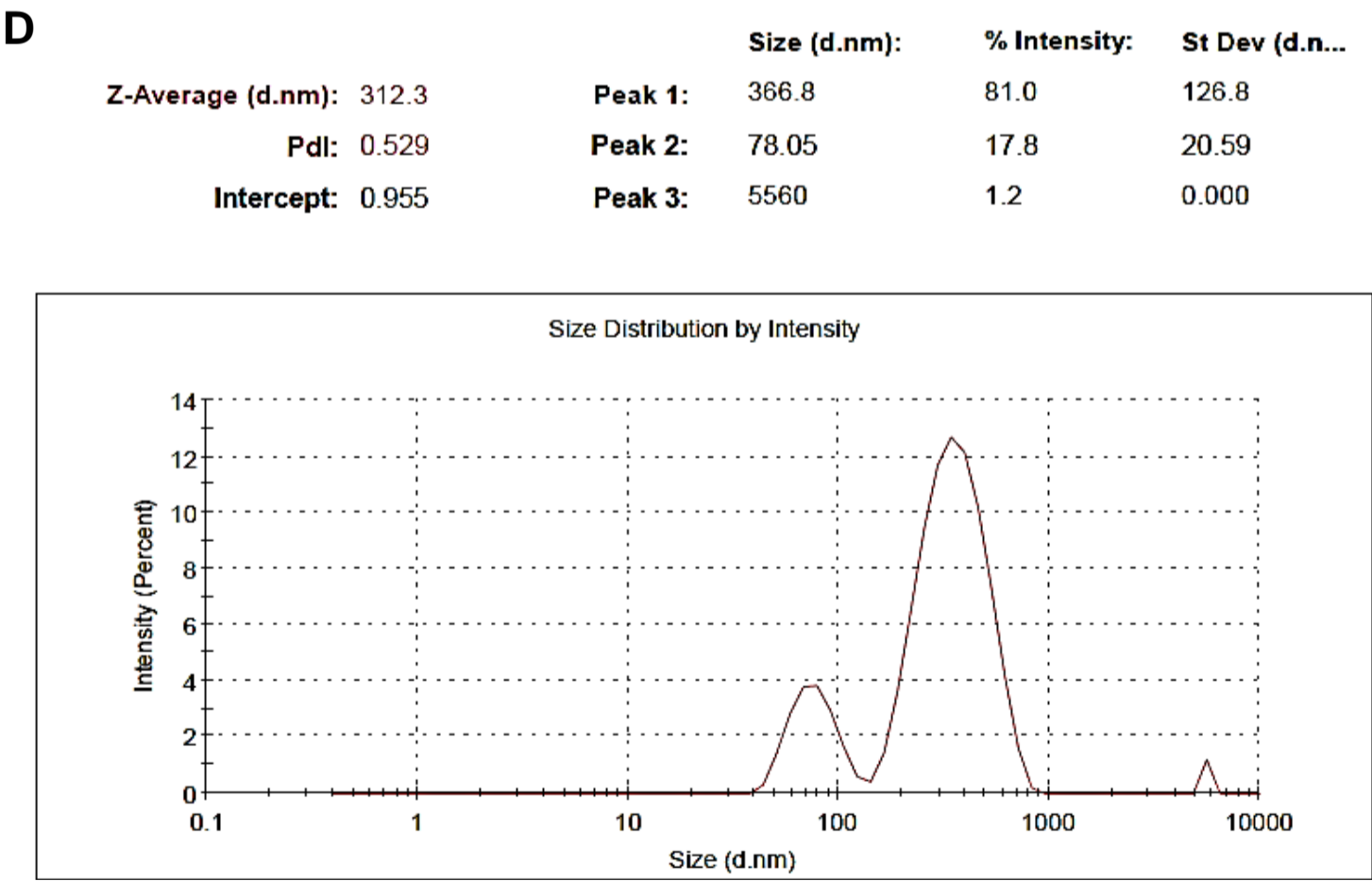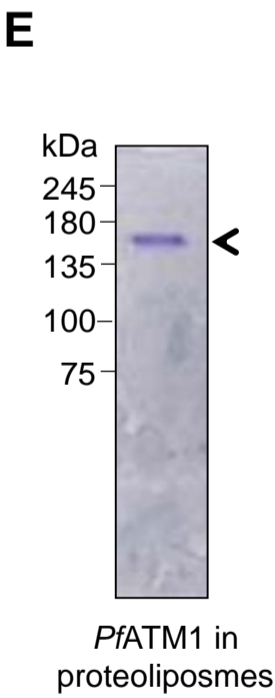

Supplement: S9 Fig — (A-B) ATPase activity of PfATM1 as a function of time (A) and protein concentration (B). (C) Change in intrinsic tryptophan fluorescence of apo-PfATM1 with increasing concentrations of GSSG. (D) Dynamic light scattering scan for determination of average size of reconstituted PfATM1-proteoliposomes. (E) The incorporation of PfATM1 confirmed by SDS-PAGE of proteoliposomes. (PDF) [file ppat.1012593.s009.pdf]
