# Supplementary material for: ATM1, an essential conserved transporter in Apicomplexa, bridges mitochondrial and cytosolic [Fe-S] biogenesis
Source: PLoS Pathog. 2024 Sep 30;20(9):e1012593. doi: 10.1371/journal.ppat.1012593 (PMC11476691; doi:10.1371/journal.ppat.1012593)
Supplement: S6 Fig — (A) Molecular structure model of PfATM1 (cyan) shown in Fig 5I superimposed on the NaATM1 template (gray). The predicted TM6 in PfATM1 (residues 636–662, gold) overlays on the NaATM1 TM6 helix (residues 312–338, salmon). (B) Molecular structure model of TgATM1 (cyan) shown in Fig 5J superimposed on the NaATM1 template (gray). (C) MD simulation of PfATM1 docked with GSSG. RMSD plot after molecular dynamics simulation of PfATM1 dimer docked with GSSG and ATP. (D) 2-D interaction plot of two GSSG molecules with the two PfATM1 chains. (E) Spectrofluorometry of buffer alone and increasing concentrations of GSSG in buffer (as control for Fig 5L) (top). a.u. denotes arbitrary units for fluorescence intensity. The absorbance spectral scan of GSSG (in buffer) in the 300–400 nm range (bottom) to rule out an inner filter effect in Fig 5L. (F) Electrostatic potential map of the PfATM1 dimer model generated by PyMOL. The positively charged central cavity is encircled. (G) MD simulation (200 nsec) showing RMS fluctuation in gatekeeper residues of CtATM1 (PDB:7PRO) and the PfATM1 model. (PDF) [file ppat.1012593.s006.pdf]

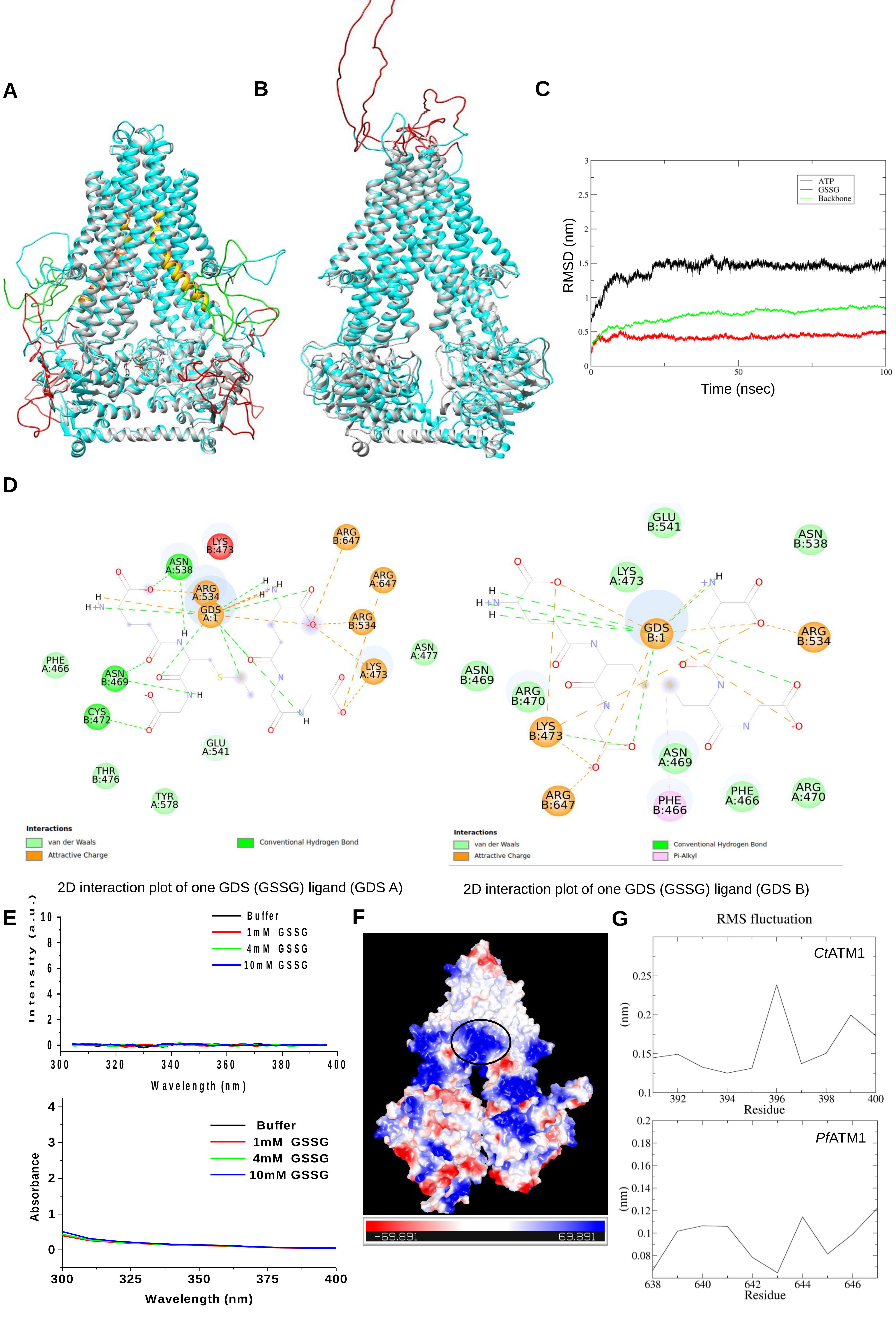
A
B
C
RMSD (nm)
Time (nsec)
ATP
GSSG
Backbone
D
LYS B:473
ASN A:538
ARG A:534
GDS A:1
ARG B:647
ARG A:647
ARG B:534
LYS A:473
ASN A:477
PHE A:466
ASN B:469
CYS B:472
GLU A:541
THR B:476
TYR A:578
Interactions
van der Waals
Attractive Charge
Conventional Hydrogen Bond
2D interaction plot of one GDS (GSSG) ligand (GDS A)
GLU B:541
ASN B:538
LYS A:473
GDS B:1
ARG B:534
ASN B:469
ARG B:470
LYS B:473
ASN A:469
ARG B:647
PHE B:466
PHE A:466
ARG A:470
Interactions
van der Waals
Attractive Charge
Conventional Hydrogen Bond
Pi-Alkyl
2D interaction plot of one GDS (GSSG) ligand (GDS B)
E
Intensity (a.u.)
Wavelength (nm)
Buffer
1mM GSSG
4mM GSSG
10mM GSSG
Absorbance
Wavelength (nm)
Buffer
1mM GSSG
4mM GSSG
10mM GSSG
F
−69.891
69.891
G
RMS fluctuation
CtATM1
(nm)
Residue
PfATM1
(nm)
Residue
